# Personalised therapeutic approaches to glioblastoma: A systematic review

**DOI:** 10.3389/fmed.2023.1166104

**Published:** 2023-04-14

**Authors:** Oliver D. Mowforth, Jamie Brannigan, Marc El Khoury, Celine Iswarya Partha Sarathi, Harry Bestwick, Faheem Bhatti, Richard Mair

**Affiliations:** ^1^Division of Neurosurgery, Department of Clinical Neurosciences, University of Cambridge, Cambridge, England, United Kingdom; ^2^Cancer Research UK Cambridge Institute, University of Cambridge, Cambridge, England, United Kingdom; ^3^School of Clinical Medicine, University of Cambridge, Cambridge, England, United Kingdom

**Keywords:** Glioblastoma, glioma, genomics, personalised therapy, survival

## Abstract

**Introduction:**

Glioblastoma is the most common and malignant primary brain tumour with median survival of 14.6 months. Personalised medicine aims to improve survival by targeting individualised patient characteristics. However, a major limitation has been application of targeted therapies in a non-personalised manner without biomarker enrichment. This has risked therapies being discounted without fair and rigorous evaluation. The objective was therefore to synthesise the current evidence on survival efficacy of personalised therapies in glioblastoma.

**Methods:**

Studies reporting a survival outcome in human adults with supratentorial glioblastoma were eligible. PRISMA guidelines were followed. MEDLINE, Embase, Scopus, Web of Science and the Cochrane Library were searched to 5th May 2022. Clinicaltrials.gov was searched to 25th May 2022. Reference lists were hand-searched. Duplicate title/abstract screening, data extraction and risk of bias assessments were conducted. A quantitative synthesis is presented.

**Results:**

A total of 102 trials were included: 16 were randomised and 41 studied newly diagnosed patients. Of 5,527 included patients, 59.4% were male and mean age was 53.7 years. More than 20 types of personalised therapy were included: targeted molecular therapies were the most studied (33.3%, 34/102), followed by autologous dendritic cell vaccines (32.4%, 33/102) and autologous tumour vaccines (10.8%, 11/102). There was no consistent evidence for survival efficacy of any personalised therapy.

**Conclusion:**

Personalised glioblastoma therapies remain of unproven survival benefit. Evidence is inconsistent with high risk of bias. Nonetheless, encouraging results in some trials provide reason for optimism. Future focus should address target-enriched trials, combination therapies, longitudinal biomarker monitoring and standardised reporting.

## Introduction

Glioblastoma is the most common and malignant primary brain tumour (1, 2). It accounts for 48% of all primary central nervous system (CNS) cancers, with estimated annual incidence and prevalence of 3.2 and 9.2 per 100,000 population in North America (3). Incidence is 1.6 times higher in males and greater than 2 times higher in Caucasians than in Black and Asian populations (2, 4).

The nomenclature of CNS tumours is rapidly evolving. The 2021 WHO classification characterised all glioblastoma as *IDH* wild type, with *IDH* mutant glioblastoma eliminated as a term and classified within *IDH* mutant astrocytoma (5). Nonetheless, aetiology remains unknown, with ionizing radiation the only proven environmental risk factor (6, 7). With the exception of hereditary tumour syndromes (8), glioblastoma appears sporadic with no proven genetic predisposition (2, 9).

Standard therapy is maximal safe surgical resection (10, 11), adjuvant radiotherapy and temozolomide (TMZ) chemotherapy, maintenance TMZ and, in some countries, electromagnetic tumour treating fields (12–14). Despite the economic burden of current therapy often amassing hundreds of thousands of US dollars (15), glioblastoma cannot be cured; the disease is rapidly and uniformly fatal with a median on-treatment overall survival of 14.6 months and a 5-year survival rate of 5% (16, 17). With a prognosis that has not improved in the past 3 decades and an average number of life years lost, that at 20.1 years, is the highest of any cancer, therapeutic advances are gravely needed (2, 18).

**Figure 1:**
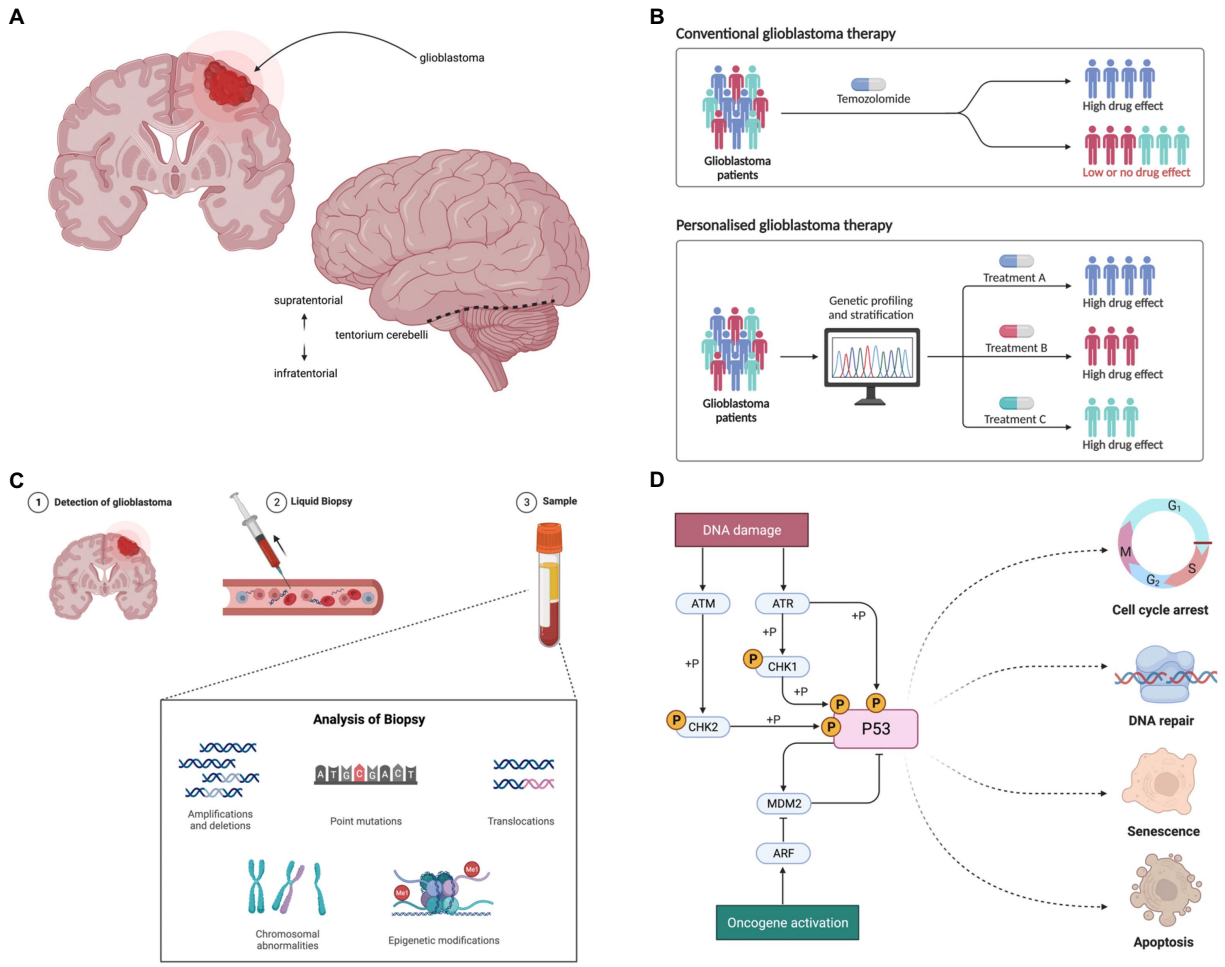
**(A)** Glioblastoma presents as a space occupying tumour within the central nervous system, most commonly occurring within the supratentorial region. **(B)** Personalised medicine aims to tailor therapeutics to individual patients to maximise efficacy: numerous genomic variants have been identified as potential targets in glioblastoma. **(C)** Molecular targets in malignant glioblastoma cells evolve over time in response to selection pressures, such as exposure to therapies. Non-invasive liquid biopsy holds significant potential as a facilitator of personalised therapy; longitudinal monitoring of tumour biomarkers may permit dynamic optimisation of targeted therapies. **(D)** The p53 pathway is one of the key cellular signalling pathways in which variants have been identified in malignant glioblastoma cells, including in *MDM2* and *TP53*. The redundancy seen within and between cellular pathways likely contributes to therapeutic failure of single agents. Figure created using BioRender.com.

A surgical cure appears unattainable due to the highly infiltrative nature of disease, spreading far beyond intraoperative and radiological margins(Figure 1A) (19, 20). Nonetheless, a promising approach lies in personalised medicine (21). This encompasses a paradigm shift from disease-centric therapeutics that overlook inter-patient variation (22), towards individualised and precise targeting of unique patient characteristics (Figure 1B).

Glioblastoma appears particularly suited to biomarker-driven personalised genomic medicine. It was the first cancer to undergo comprehensive genomic analysis in the Cancer Genome Atlas project (23) and characterisation of variants in genes and pathways has rapidly accrued (24–26). Furthermore, glioblastoma demonstrates high inter- and intra-tumour spatial and temporal genomic heterogeneity (27–29), predicting targeted combination therapies may have higher efficacy than indiscriminate single agents. For example, mutually exclusive *EGFR* and *PDGFRA* oncogene amplification in intermingled subclones of glioblastoma cells (30) requires simultaneous inhibition of both for pathway inhibition *in vitro* (31). Moreover, the dynamic nature of genomic heterogeneity appears to fuel recurrence and resistance; therapy drives clonal evolution secondary to novel mutational events and selection of resistant subclones (32). Longitudinal biomarker monitoring may facilitate dynamic adjustment of personalised therapy (Figure 1C), with current focus on non-invasive methods (33, 34).

An extensive number of targets for personalised glioblastoma medicine have been identified. For example, variants disrupting receptor tyrosine kinase (RTK) pathways have been characterised, including amplification and/or mutation in *EGFR*, *KIT*, *PDGFRA*, *FGFR1*, *FGFR3*, and *MET* (28, 35). Variants have also been described in the PI3K/Akt/mTOR and MAPK signalling pathways, including in *PTEN*, *PIK3CA*, *NF1*, and *BRAF* (36). In addition, variants in cycle control genes, such as *MDM2* and *TP53* in the p53 pathway (Figure 1D) and *CDK4*, *CDK6* and *RB1* in the Rb pathway have been identified (28, 37). Despite this extensive genomic characterisation, the functional, biological and clinical relevance of the majority of variants is currently poorly characterised (20), making target prioritisation a major challenge (38, 39).

Furthermore, several vaccine-based personalised approaches have been explored (40). Vaccines stimulate tumour-specific immune responses to injected antigens, aiming to overcome some of the unique challenges of glioblastoma, including an immunosuppressive tumour microenvironment, low mutational burden and a relatively immunologically isolated location within the CNS (41, 42). In addition, clinical trials of non-vaccine immunotherapies are also ongoing, including immune checkpoint inhibitors and chimeric-antigen receptor (CAR) T cell therapies (41).

Despite conceptual rationale and pre-clinical data, clinical trials have so far been unable to demonstrate survival efficacy for most targeted glioblastoma therapies (20). However, most trials of targeted therapies have not been personalised due to lacking biomarker enrichment. For example, whilst 20–30% of glioblastoma is estimated to express EGFRvIII (20, 35), any efficacy signal from patients expressing the variant may be lost in a trial in which most patients would not be expected to express this target. Furthermore, nearly all trials have studied single agents, overlooking the redundancy within molecular pathways. Given the substantial need for interventions that improve glioblastoma survival, personalised targeted therapies should be rigorously and fairly evaluated before being discounted.

The aim of this quantitative systematic review of effectiveness was therefore to synthesise the current evidence on survival efficacy of personalised therapeutic approaches in glioblastoma. The objective was to evaluate past approaches and identify possible future directions.

## Methods

### Study design

A systematic review was conducted with reference to the Preferred Reporting Items for Systematic Reviews and Meta-Analyses (PRISMA) 2020 checklists (Appendix F) (43, 44).

### Eligibility criteria

#### Inclusion criteria

- Adult ≥ 18 years;
- Human study;
- English language;
- Supratentorial glioblastoma population, including extraction of glioblastoma-specific data from a broader cohort;
- Any personalised therapy including genomic, transcriptomic, proteomic and vaccines;
- Clinical trial or observational study;
- Survival outcome.

#### Exclusion criteria

- Review or meta-analysis;
- Case report;
- Letter;
- Editorial;
- Opinion article;
- Correction.

### Information sources

MEDLINE, Embase, Web of Science, Scopus and the Cochrane Library were searched from inception to 5th May 2022. MEDLINE and Embase searches were performed using the Ovid platform (Ovid Technologies, New York, NY, United States). ClinicalTrials.gov was searched on 25th May 2022; references lists of included studies and related review articles were hand searched for additional studies.

### Search strategy

Scoping searches were performed to refine the review question. Final search strategies (Appendix A) were developed and piloted using an iterative process. To maximise sensitivity, no automated search limits were applied. Scottish Intercollegiate Guidelines Network (SIGN) randomised controlled trial (RCT) and observational study search filters were included in the MEDLINE and Embase searches (45). Cochrane RCT search filters were utilised in the Web of Science and Scopus searches (46); the key words from the SIGN MEDLINE observational study filter were used to capture observational studies in Web of Science and Scopus because observational study filters do not exist for these databases. Search sensitivity was evaluated using a list of 8 papers known to meet inclusion criteria: all papers were successfully captured.

### Selection process

De-duplication of search results was completed in EndNote (Version 20.3.0.17787, Clarivate, London, United Kingdom) (47). Title and abstract screening were completed using Rayyan (Rayyan Systems Inc., Cambridge, MA, United States). All records were screened in duplicate by 2 blinded reviewers (ODM and JB/MEK/CPS/HB/FB); a pilot of 100 records were screened by all reviewers to ensure concordance. On screening completion, 146 conflicts from 6,738 screening decisions were resolved by discussion. Full-text screening was completed in duplicate (ODM and JB/CPS/MEK).

### Data collection

Manual data extraction was completed in duplicate (ODM and JB/CPS/MEK) in Microsoft Excel (Version 16.63, Microsoft 365) using a piloted extraction form.

### Data items

Data were sought for any survival outcome. Survival is typically measured as progression-free survival (PFS) and overall survival (OS). PFS is defined as the length of time from randomisation or initiation of treatment to disease progression or death; OS is the length of time from diagnosis or randomisation or initiation of treatment until death (48, 49). Participant and study characteristics for each included study were also sought (Appendix C).

### Risk of bias assessment

Risk of bias of included studies was assessed in duplicate (ODM and MEK/JB/CP) using the Joanna Briggs Institute critical appraisal tools checklists (50).

### Synthesis methods

Due to the heterogeneity in inclusion criteria, baseline characteristics and interventions of included studies, meta-analysis was not possible. A qualitative synthesis without meta-analysis (SWiM) (51) was therefore conducted. To facilitate synthesis, survival in individual studies was scored on a 4-point scale: associated with survival benefit, appears beneficial, appears unbeneficial and associated with no survival benefit. Only studies reporting an appropriate statistical test comparing survival between an intervention group and a control group were scored as associated with benefit or no benefit. Where no appropriate statistical test was performed, a score of appears beneficial or appears unbeneficial was assigned with reference to established survival data for standard of care therapy (12).

### Certainty assessment

Confidence in the body of evidence was assessed using the Grading of Recommendations, Assessment, Development and Evaluations (GRADE) framework (52).

## Results

### Study selection

**Figure 2:**
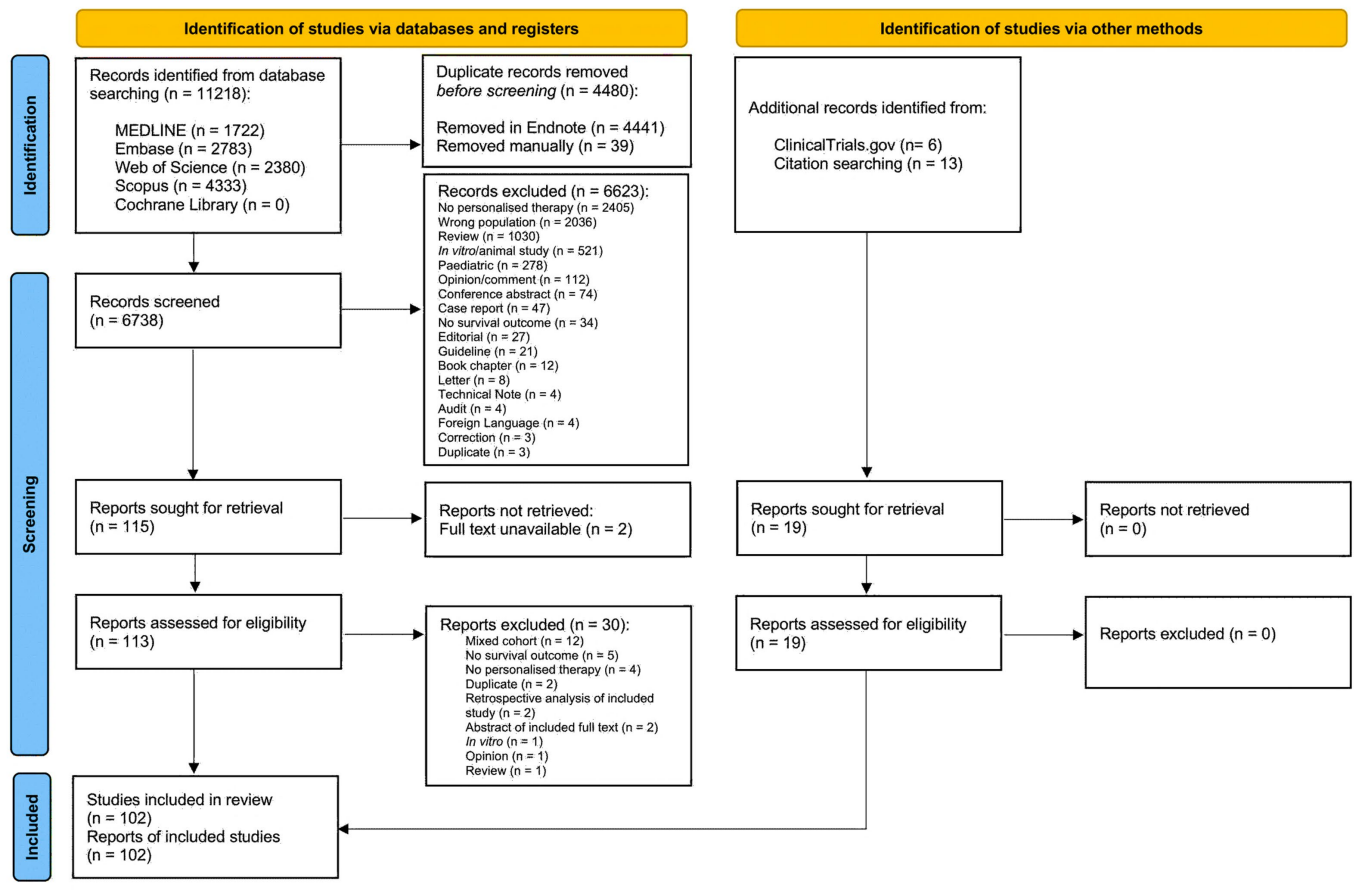
Preferred Reporting Items for Systematic Reviews and Meta-Analyses (PRISMA) flow diagram of study selection. Following the removal of duplicates, titles and abstracts of 6,738 records were screened and 115 studies were sought for retrieval. An additional 19 studies were identified from ClinicalTrials.gov and by hand searching reference lists of included studies and relevant review articles. Reports for 2 studies were not retrievable and 30 studies that initially appeared to meet inclusion criteria were excluded during full text screening (Appendix B). A total of 93 full text articles and 9 conference abstracts were included in the review.

A total of 11,218 records were identified from database searching; 102 studies were included in the review (Figure 2).

### Study characteristics

**Figure 3:**
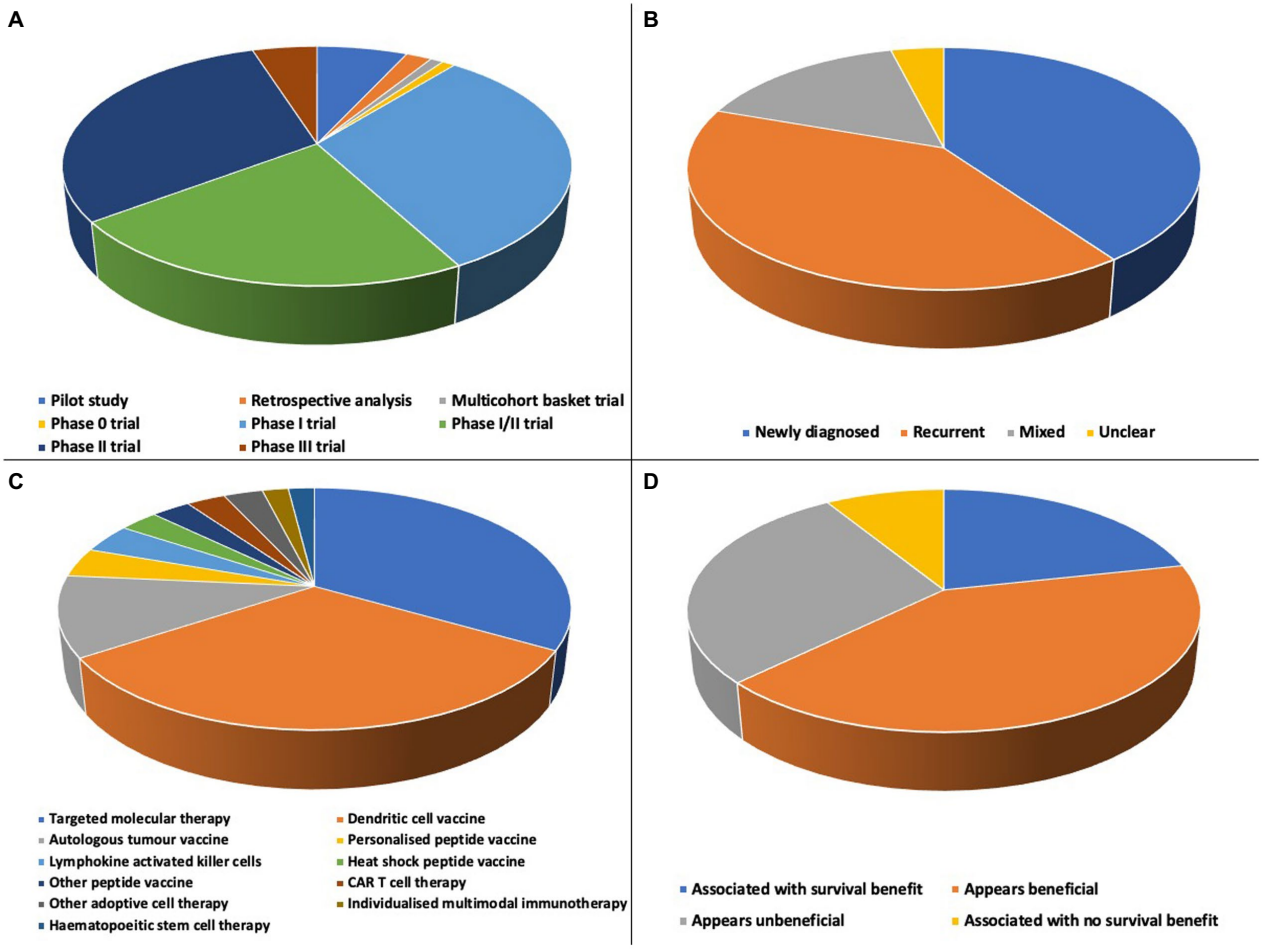
**(A)** There were a range of designs of included studies: 7 pilot studies, 2 retrospective analyses, 1 multicohort basket trial, 1 phase 0 trial, 32 phase I trials, 23 phase I/II trials, 31 phase II trials and 5 phase III trials. **(B)** Newly diagnosed and recurrent disease cohorts were each studied by 40.2% of included trials, 15.7% (16/102) included mixed cohorts and the status of patients was unclear for 3.9% (4/102) of trials. **(C)** More than 20 distinct types of personalised therapy were studied, including a range of targeted molecular therapies and dendritic cell vaccine approaches. **(D)** Overall survival efficacy of all personalised therapies; in 62.7% (64/102) of studies there was a survival benefit or personalised therapy appeared beneficial.

**Figure 4:**
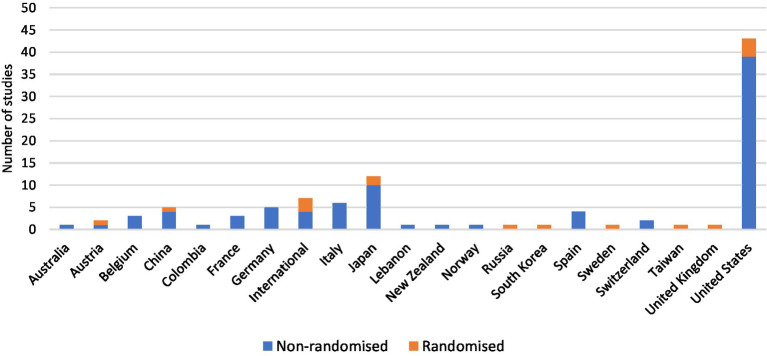
Personalised therapy trials were mostly non-randomised and were conducted in more than 20 countries worldwide. The United States conducted the most trials with a total of 42.2% (43/102), followed by Japan with 11.8% (12/102).

All included studies were interventional, of which 15.7% (16/102) were randomised trials, 84.3% (86/102) non-randomised trials and 84.3% (86/102) phase I, phase I/II or phase II trials (Figure 3A). Trials were conducted in 20 countries, with 7 international trials (Figure 4). Newly diagnosed patients and patients with recurrent disease were each studied by 40.2% (41/102) of trials (Figure 3B). Seventy-nine trials exclusively studied glioblastoma; 23 trials included glioblastoma within broader cohorts (Supplementary Table 1).

### Risk of bias

Randomisation, allocation concealment and intention to treat analysis were poorly reported for randomised trials (Appendix D). For non-randomised trials, the similarity of comparison groups was not always clear, and it was common for studies to lack a control group.

### Patient characteristics

A total of 5,527 patients were included (Supplementary Table 2). Ten trials included greater than 100 patients; the mean number of patients per trial was 54. A minority of trials (37.3%, 38/102) included a control group. The mean age of patients included in all trials was 53.7 years and mean Karnofsky Performance Status was 82%. A mean of 59.4% of patients were male. Ethnicity was reported by 9.8% of trials (10/102); a mean of 88.1% of patients were of white ethnicity. A mean of 62.2% of patients had undergone complete surgical resection before personalised therapy; 34.9% had *MGMT* promoter methylated tumours. Time from diagnosis to personalised therapy ranged from 10 days to greater than 1 year. The mean reported objective response rate was 18.9%.

### Survival following personalised therapy

More than 20 types of personalised therapy were studied (Figure 3C; Supplementary Table 3). Targeted molecular therapies were the most studied (33.3%, 34/102) followed by autologous dendritic cell vaccines (32.4%, 33/102) and autologous tumour vaccines (10.8%, 11/102). Reporting of prior and concurrent therapies was inconsistent; all patients had completed at least surgical resection and radiotherapy, with most also receiving concurrent temozolomide chemotherapy.

### Targeted molecular therapies

Targets included the epidermal growth factor receptor (EGFR), particularly the EGFRvIII variant, vascular endothelial growth factor receptor (VEGF), MET-kinase, cyclin-dependent kinases 4 and 6 (CDK4/6), phosphatidylinositol 3-kinase (PI3K), and programmed cell death protein 1 (PD1).

The EGFRvIII peptide vaccine rindopepimut was the most studied targeted molecular therapy, including 2 randomised and 4 non-randomised trials. Weller et al. (53) conducted a double-blind, placebo-controlled trial of 745 newly diagnosed patients and found no difference in PFS or OS between treated patients and controls in either ITT analysis or analysis of only patients with minimal residual disease after chemotherapy and radiotherapy. A subsequent double-blind, placebo-controlled, randomised phase II trial of rindopepimut in combination with the anti-VEGF monoclonal antibody bevacizumab in 73 patients with recurrent disease demonstrated a survival advantage in the treated group (54). In addition, rindopepimut was associated with a survival benefit (55, 56) or appeared beneficial (57, 58) in 4 non-randomised trials studying a total of 134 patients.

There were 2 randomised and 3 non-randomised trials of the EGFR monoclonal antibody-drug conjugate Depatuxizumab mafodotin. A randomised double-blind placebo-controlled phase III trial of 639 newly diagnosed patients reported no survival difference between treated and control patients (59); a randomised open-label phase II trial of 260 patients with recurrent disease found prolonged survival in the treated group in long-term follow up analysis but not in primary efficacy analysis at 15 months (60). The 3 non-randomised trials had mixed survival results (61–63).

A total of 9 of the non-rindopepimut/depatuxizumab molecular therapies were also targeted at EGFR (64–72) and a further 3 trials studied other receptor tyrosine kinase inhibitors (73–75). These trials were all non-randomised and had mixed survival results.

Personalised chemotherapy regimens informed by *in vitro* drug sensitivity testing on autologous resected tumour cells appeared beneficial in 2 non-randomised trials studying a total of 105 patients (76, 77). However, 3 non-randomised trials using genomic-profiling to guide personalised molecular therapy had mixed survival results (78–80).

### Dendritic cell vaccines

The most studied vaccine therapies (17%, 17/102) consisted of autologous dendritic cells pulsed with autologous tumour lysates (ATL/DC). There were four reports of ATL/DC randomised trials, the largest of which reported interim data on 331 patients in a double-blind, placebo-controlled cross-over trial in patients with newly diagnosed glioblastoma (81). This trial reported mOS of 23.1 months and described a subgroup consisting of 30% of the ITT population that showed an extended mOS of 40.5 months that could not be explained by known prognostic factors. Furthermore, Cho et al. (82) conducted a randomised phase II trial of 34 patients, reporting significantly prolonged OS in a group of patients receiving ten subcutaneous ATL/DC vaccinations in addition to standard of care therapy compared to a control group receiving standard of care therapy alone. However, two other randomised-controlled phase II studies on 76 and 58 patients found no survival benefit associated with ATL/DC (83, 84). A total of 13 non-randomised trials had mixed results with 11 in favour (85–95) and two unsupportive (96, 97) of a survival benefit of ATL/DC.

In addition, three non-randomised trials studying vaccination with autologous tumour peptide pulsed DCs in a total of 198 patients reported significantly improved survival in treated patients (98, 99) or survival that appeared to be beneficial (100). Two trials, including a randomised trial by Mitchell et al. (101, 102), studied vaccination with cytomegalovirus phosphoprotein 65 RNA pulsed DCs, both reporting a significant association between therapy and prolonged survival. Two non-randomised trials studied vaccines consisting of DCs pulsed with mRNA from tumour stem cells in a total of 105 patients; median PFS was 2.9 times longer in vaccinated newly diagnosed patients compared to controls (103), however there was no survival benefit demonstrated in patients with recurrent disease (104).

Furthermore, a randomised double-blind placebo-controlled phase II trial of DCs pulsed with synthetic peptide epitopes targeting glioblastoma tumour and stem-cell associated antigens (ICT-107) included 124 patients and found prolonged PFS but not OS in treated patients compared to controls (105). An earlier non-randomised trial of ICT-107 in 16 patients reported an association between expression of target antigens and significantly prolonged PFS and OS in newly diagnosed patients (106).

Other DC vaccine approaches included pulsing autologous DCs with antigens including glioma stem cell like antigens (107), personalised mRNA tumour associated antigens (108) and allogeneic cells from glioma cell lines (109). Efficacy ranged from being associated with a survival benefit (107), to appearing beneficial (108, 110, 111), to appearing unbeneficial (109, 112, 113).

### Other vaccines

The overall effect on survival was unclear across 2 randomised and 9 non-randomised trials of autologous tumour vaccines. Neither randomised trial was able to demonstrate a difference between treated patient and controls, including a double-blind placebo-controlled phase IIb/III trial of 60 newly diagnosed patients (114). However, a non-randomised phase I/II trial of 110 newly diagnosed patients in which tumour cells were infected with Newcastle Disease Virus before vaccination reported significantly longer PFS and OS in vaccinated patients compared to controls (115).

In single non-randomised vaccine trials, Wilms tumour 1 peptide (116), survivin peptide (117) and 9 HLA-A2 restricted peptides eluted from the surface of glioblastoma samples combined with 2 HLA-class II-binding peptides (118) all appeared beneficial for survival. However, four trials of personalised peptide vaccines (119–122), 1 of which was randomised, and 3 non-randomised trials of autologous heat shock protein peptide complex-96 (123–125) had overall mixed survival results.

### Other immunotherapies

Overall, there were conflicting survival results for adoptive cell therapies. A randomised open-label phase III trial of intravenous autologous cytokine-induced killer cells in 180 patients reported prolonged PFS but not OS in treated patients (126); a randomised phase II trial of autologous lymphoid effector cells specific against tumour (ALECSAT) in 62 patients found no significant survival difference between treated and control patients (127). A further non-randomised phase I study conducted by Smith et al. (128) reported favourable survival in 25 patients treated with autologous cytokine-specific T cells; patients treated before recurrence had significantly improved OS than patients who had progressed. In addition, 3 non-randomised trials of chimeric antigen receptor (CAR) T cell therapy, two of which were directed towards EGFRvIII, appeared unbeneficial for survival (129–131).

Two non-randomised trials of haematopoietic stem cell therapies studying a total of 10 patients reported results that appeared beneficial (132, 133), whilst 4 non-randomised trials of lymphokine activated killer cells, 3 of which studied patients with recurrent disease, had mixed survival results (134–137). Finally, individualised multimodal immunotherapy (IMI) appeared beneficial when studied in 164 patients across 2 non-randomised trials conducted by van Gool et al. One trial studied newly diagnosed patients and the other a mixed population (138, 139). The trial in newly diagnosed patients reported significantly prolonged survival in patients treated with IMI and concurrent temozolomide compared to IMI alone (139).

### Reporting bias

A total of 93.1% (95/102) of studies reported OS, 71.6% (73/102) reported PFS and 58.8% (60/102) reported at least 1 additional survival outcome. Additional survival outcomes were reported at time points that differed between studies. Moreover, it was not uncommon for a survival outcome specified amongst *a priori* endpoints not to be reported.

### Certainty of evidence

Certainty of the body of evidence was low for all therapies (Table 1). There was a high risk of bias due to most trials being small and non-randomised. Associations between therapies and survival were frequently conflicting between trials, including between the highest quality randomised trials.

**Table 1:** Summary and certainty of evidence.

Therapy	Number of trials	Number randomised	Number non-randomised	Survival efficacy	Overall efficacy	Certainty of evidence (GRADE)
Non-rindopepimut/depatuxizumab targeted molecular therapy	23	0	23	Associated with benefit 1NR Appears beneficial 8NR Appears unbeneficial 13NR Associated with no benefit 1NR	Unclear	⊕ ⊕ ⊝⊝ Low Due to risk of bias and inconsistency
Vaccine of autologous DCs pulsed with autologous tumour lysate	17	4	13	Associated with benefit: 1R 4NR Appears beneficial: 1R 7NR Appears unbeneficial: 2NR Associated with no benefit: 2R	Likely beneficial	⊕ ⊕ ⊝⊝ Low Due to risk of bias and inconsistency
Autologous tumour vaccine	11	2	9	Associated with benefit 2NR Appears beneficial 5NR Appears unbeneficial 1R 1NR Associated with no benefit 1R 1NR	Unclear	⊕ ⊕ ⊝⊝ Low Due to risk of bias and inconsistency
Rindopepimut (EGFRvIII peptide vaccine)	6	2	4	Associated with benefit 1R 2NR Appears beneficial 2NR Associated with no benefit 1R	Unclear	⊕ ⊕ ⊝⊝ Low Due to risk of bias and inconsistency
Depatuxizumab mafodotin (EGFR mAb-drug conjugate)	5	2	3	Appears beneficial 1R 2NR Appears unbeneficial 1NR Associated with no benefit 1R	Unclear	⊕ ⊕ ⊝⊝ Low Due to risk of bias and inconsistency
Personalised peptide vaccine	4	1	3	Appears beneficial 2NR Appears unbeneficial 1NR Associated with no benefit 1R	Unclear	⊕ ⊕ ⊝⊝ Low Due to risk of bias and inconsistency
Lymphokine activated killer cells	4	0	4	Associated with benefit 1NR Appears beneficial 1NR Appears unbeneficial 2NR	Unclear	⊕ ⊕ ⊝⊝ Low Due to risk of bias and inconsistency
Vaccine of autologous DCs pulsed with autologous tumour peptides	3	0	3	Associated with benefit 2 NR Appears beneficial 1 NR	Beneficial	⊕ ⊕ ⊝⊝ Low Due to risk of bias
Other peptide vaccine	3	0	3	Appears beneficial 3NR	Beneficial	⊕ ⊕ ⊝⊝ Low Due to risk of bias
HSPPC-96 vaccine	3	0	3	Appears beneficial 2NR Appears unbeneficial 1NR	Unclear	⊕ ⊕ ⊝⊝ Low Due to risk of bias and inconsistency
CAR T cell therapy	3	0	3	Appears unbeneficial 3NR	No benefit	⊕ ⊕ ⊝⊝ Low Due to risk of bias
Other adoptive cell therapy	3	2	1	Associated with benefit 1R Appears beneficial 1NR Associated with no benefit 1R	Unclear	⊕ ⊕ ⊝⊝ Low Due to risk of bias and inconsistency
Vaccine of autologous DCs pulsed with cytomegalovirus pp65 RNA	2	1	1	Associated with benefit 1R 1NR	Beneficial	⊕ ⊕ ⊝⊝ Low Due to risk of bias
Vaccine of autologous DCs pulsed with peptides targeting tumour/stem cell-associated antigens (ICT-107)	2	1	1	Associated with benefit 1R Appears beneficial 1NR	Beneficial	⊕ ⊕ ⊝⊝ Low Due to risk of bias
Vaccine of autologous DCs pulsed with tumour stem cell RNA	2	0	2	Associated with benefit 1NR Appears unbeneficial 1NR	Unclear	⊕ ⊕ ⊝⊝ Low Due to risk of bias and inconsistency
Individualised multimodal immunotherapy	2	0	2	Associated with benefit 1NR Appears beneficial 1NR	Beneficial	⊕ ⊕ ⊝⊝ Low Due to risk of bias
Haematopoietic stem cell therapy	2	0	2	Associated with benefit 1NR Appears beneficial 1NR	Beneficial	⊕ ⊕ ⊝⊝ Low Due to risk of bias

## Discussion

### Summary of main findings

The objective of this systematic review was to synthesise evidence on the survival efficacy of personalised therapies in glioblastoma. Most studies reported either a survival benefit or survival that appeared beneficial following a personalised therapy (Figure 3D), however there was no consistent high-quality evidence of efficacy for any individual personalised therapy.

### Context of findings

Central nervous system tumours have historically been characterised using histological features such hypercellularity, nuclear atypia and necrosis (140). Nomenclature has evolved with each iteration of the WHO classification, driven by increasingly precise molecular characterisation (5). This is revealing increasing heterogeneity of tumour types in historical trials. Molecularly distinct tumours may require distinct therapeutic approaches. Therefore, the utility of past trials may diminish with time, with heterogeneity likely contributing to lack of efficacy in non-molecularly defined populations, specifically that associated with *IDH* mutation and the inclusion of patients classified, currently, as astrocytoma grade 4. This is to some extent circumvented in this review, which studied only molecularly selected patient cohorts and excluded therapies applied in a non-personalised manner.

Personalised therapy should target unique features of each patient’s disease. Included trials mostly considered single agents, which is problematic given a single molecular difference is highly unlikely to define tumour identity. Furthermore, variants do not occur in isolation but affect cellular pathways and networks of molecules, in which there is redundancy and adaptation (20). Therefore, it is unsurprising that single agent efficacy is underwhelming. As profiling of tumours becomes increasingly precise, more detailed characterisation of differences between tumours will follow, including how variants synergise and should be prioritised therapeutically. Only a small number of currently known variants have been targeted, with EGFR a major focus. Combination approaches may offer superior efficacy, with promising pre-clinical and early clinical examples (53, 54, 141, 142). However, more therapeutic options are needed to address already identified targets. Due to its low mutational burden, glioblastoma is an ideal cancer candidate in which to pursue this study.

In addition, a number of trials reported associations of therapy with prolonged PFS but not OS (103, 105, 126). PFS and OS are strongly correlated outcomes, with PFS offering earlier assessment and higher statistical power at time of analysis (143). However, progression is technically challenging to decipher from pseudoprogression, a not infrequent response following chemoradiotherapy or immunotherapy in which a tumour initially increases in size or new lesions appear (144). PFS is therefore less reproducible, reflected in many trials mandating centralised assessment of progression (145).

### Limitations of included studies

#### Heterogenous measurement and reporting of survival

Overall, survival measurements were poorly described, limiting confidence in comparisons. A total of 49% of trials did not specify how survival was measured and those that did reported various start points including diagnosis, recurrence and commencement of therapy. The majority reported median survival, whilst others reported mean values; rates of OS and PFS were quoted at unstandardised time intervals. Furthermore, whilst PFS and TTP are strictly distinct measures (146), this terminology appeared to be used interchangeably by some included trials (64, 77).

#### Quality of evidence

The overall quality of evidence is low. Many trials were small, non-randomised and single arm. Whilst early phase trials and proof of principle studies have value in assessing safety and feasibility, they are not powered for survival analysis, selection criteria often limit generalisability and patients are exposed to unstandardised schedules and doses of therapy. Survival reported by these trials should therefore be interpreted cautiously. Furthermore, trials included many uncontrolled variables including surgical factors, past chemotherapy drugs and doses and radiotherapy regime before and after personalised therapy. In addition, many trials used historical control groups, which provide a convenient comparison but with higher risk of confounding.

#### Methodological challenges

The focus of trials was on short-term survival rates, in the context of a disease that has very low rates of long-term survival. However, it is becoming clear that survival efficacy of several immunotherapies may lie in a small subgroup of long-term survivors (81). Greater focus on this subgroup may yield insight into factors driving survival efficacy to improve future therapy personalisation.

Furthermore, it was not uncommon for patients to be excluded from trials before commencing therapy due to progression (112, 147, 148). This was particularly true for vaccine therapies, which typically took up to 8 weeks to manufacture.

#### Standardisation needed to aid synthesis and promote reproducibility

Personalised glioblastoma trials would benefit from definition of core data elements, measurement and outcome sets, as significant heterogeneity exists which limits comparability and synthesis (149). For example, performance status was inconsistently reported using KPS, WHO and ECOG scales and for many datapoints there was inconsistency in whether mean, median or modal values were reported. The terms newly diagnosed and recurrent were utilised in this review to address inconsistent use of the word “primary,” referring to either newly diagnosed or arising *de novo* without evolution from a lower grade glioma (128, 137, 150, 151). In addition, there was little consensus on the definition of complete resection, varying between 90, 95 and 99% (55, 77, 113, 152).

Furthermore, there was inconsistency in inclusion criteria, with some trials stating a focus on glioblastoma but also including anaplastic astrocytoma patients (153) and other trials studying broader cohorts of glioma (64, 123, 154). In addition, most DC vaccine trials had highly precise administration schedules, for which the rationale was often unclear. Others had limited descriptions of the schedule, hindering replicability and reproducibility. Time from diagnosis to commencement of personalised therapy was often not specified or highly variable within and between studies. Furthermore, there was inconsistency in response rate calculations; standardisation in objective response rate definition outlined by the Revised Assessment in Neuro-Oncology (RANO) criteria is therefore welcomed (155).

#### Generalisability

The generalisability of many included trials is limited due to included patients being highly and heterogeneously pre-treated with other therapies. Failure of a personalised therapy in a trial of last line therapy in highly treated patients with recurrent disease should not be extrapolated beyond this context.

#### Implications and future directions

Targeted and personalised therapy are not synonymous: most trials of targeted therapies have not used target-enrichment criteria and were consequently excluded. Comprehensive molecular characterisation is needed to identify personalised spectra of targetable molecular alterations, with next generational sequencing panels of utility (156, 157). Future efficacy trials should only be studied in a population proven to express the target of interest and employ combination approaches to overcome redundancy. Furthermore, glioblastoma is a moving target; 60% of tumours expressing EGFRvIII at primary resection lose expression by recurrence (54). Genomic instability and immune evasion appear to be driven by complex interactions encompassing epigenetic changes, metabolic reprogramming and oxidative stress responses within the tumour microenvironment (158). Therefore, methods to monitor genomic heterogeneity will be essential to tailor therapy, with non-invasive strategies most favourable (Figure 1C) (34). In addition, methods to improve delivery across the blood brain barrier, such as convection enhanced delivery (159) and nanoparticle drug delivery systems (160), may catalyse the success of personalised therapies.

Finally, differences in allele frequencies between populations are well-described, as is differing glioblastoma prevalence. Unfortunately, only 10% of included studies considered ethnicity; future work should do so as differing genomic profiles between populations may prove another important consideration in personalisation of therapy.

### Conclusion

Personalised therapy remains of unproven benefit to survival for patients with glioblastoma, with no therapy currently ready for routine clinical application. Nonetheless, encouraging results in some trials provide reason for optimism. Future advances in this field may depend on target-enriched trials, dynamic combination therapies with longitudinal monitoring of genomic biomarkers and standardised reporting in clinical trials.

## Data availability statement

The original contributions presented in the study are included in the article/Supplementary material, further inquiries can be directed to the corresponding author.

## Author contributions

OM and RM: conceptualisation and design, search strategy, and data interpretation. OM, JB, MEK, CS, HB, and FB: screening and data extraction. OM: manuscript drafting. OM, JB, MEK, CS, HB, FB, and RM: manuscript revision. RM: supervision. All authors contributed to the article and approved the submitted version.

## Funding

Research in RM’s laboratory is jointly funded by Cancer Research UK and the University of Cambridge. OM is supported by an NIHR Academic Clinical Fellowship at the University of Cambridge.

## Conflict of interest

The authors declare that the research was conducted in the absence of any commercial or financial relationships that could be construed as a potential conflict of interest.

## Publisher’s note

All claims expressed in this article are solely those of the authors and do not necessarily represent those of their affiliated organizations, or those of the publisher, the editors and the reviewers. Any product that may be evaluated in this article, or claim that may be made by its manufacturer, is not guaranteed or endorsed by the publisher.
