# Supplementary material for: Personalised therapeutic approaches to glioblastoma: A systematic review
Source: Front Med (Lausanne). 2023 Apr 14;10:1166104. doi: 10.3389/fmed.2023.1166104 (PMC10140534; doi:10.3389/fmed.2023.1166104)
Supplement: Supplementary file 1 [file Data_Sheet_1.docx]

**Supplementary Materials**

Supplementary Table 1: Study Characteristics

Supplementary Table 2: Patient Characteristics

Supplementary Table 3: Survival

Appendix A: Search Strategies

Appendix B: Full Text Screening Exclusions

Appendix C: Patient and Study Characteristics Extracted

Appendix D: Risk of Bias Assessments

Appendix E: Study Funders

Appendix F: PRISMA Checklists

Appendix G: References used in Supplementary Materials

**Supplementary Table 1.** Summary of characteristics of included studies. R = randomised, NR = non-randomised, FT = full text, A= abstract, GB = glioblastoma, AA = anaplastic astrocytoma, RT = radiotherapy, CT = chemotherapy, KPS = Karnfosky Performance Status, GTR = gross total resection, CST = corticosteroid, OS = overall survival, PFS = progression free survival, TTP = time to progression, ORR = objective response rate, DCR = disease control rate, QoL = quality of life, PD = pharmacodynamic, PK = pharmacokinetic.

| Study | Country | Trial Design | Report Format | Newly Diagnosed or Recurrent | Key Inclusion Criteria | Endpoint | Start of Survival Measurement |
| --- | --- | --- | --- | --- | --- | --- | --- |
| Bloom 1973(74) | UK | R phase II | FT | - | Grade III or IV supratentorial astrocytoma Age < 70 Suitable for craniotomy | - | - |
| Merchant 1988(75) | US | NR phase I | FT | R | Supratentorial GB ≥ 1 craniotomy + RT +/- CT No antitumour therapy for ≥ 1 month | - | - |
| Lillehei 1991(76) | US | NR phase I/II | FT | R | Primary brain tumour Age ≥ 3 years Prior irradiation dose ≥ 5000 cGy No CST within 4 weeks KPS ≥ 50 | - | - |
| Hayes 1995(77) | US | NR phase I/II | FT | R | Supratentorial malignant glioma Age ≥ 18 years Candidate for re-operation Postoperative ≥ KPS 60 Anticipated survival > 4 months | OS | First or repeat surgery |
| Plautz 1998(78) | US | NR phase I | FT | Mixed | Grade III or IV malignant astrocytoma ECOG 0 to 1 Received RT +/- CT Stable without CST above physiological replacement | - | - |
| Schneider 2001(79) | Germany | NR phase I/II | FT | ND | GB Completed surgery + RT | P: peripheral immune response S: OS | Surgery |
| Yu 2001(80) | US | NR phase I | FT | ND | GB or AA KPS ≥ 60 Lowest possible maintenance dose of CST  Off CST at vaccination Completed RT after surgery | Toxicity, survival, intracranial T-cell infiltration | - |
| Iwadate 2003(81) | Japan | NR phase II | FT | ND | Supratentorial GB Age ≥ 15 years KPS ≥ 50 | P: OS | - |
| Yamanaka 2003(82) | Japan | NR phase I/II | FT | R | GB, AA or other malignant glioma Undergone surgery + RT No CT/RT for 4 weeks | Toxicity, immune response, clinical response | Disease onset |
| Steiner 2004(83) | Germany | NR phase I/II | FT | ND | GB KPS ≥ 60 Age ≥ 18 years | Feasibility, side effects, vaccine-induced immune effects, PFS, OS | - |
| Wheeler 2004(84) | US | 3 x NR phase I/II | FT | Mixed | GB KPS ≥ 60 Age ≥ 18 years | - | Diagnosis |
| Yu 2004(85) | US | NR phase I | FT | R | Malignant glioma KPS ≥ 60 Age ≥ 18 years | P: feasibility, safety, bioactivity | - |
| Liau 2005(86) | US | NR phase I | FT | Mixed | Malignant glioma Amenable to surgical resection KPS ≥ 60 No RT for ≥ 2 weeks, nitrosurea for ≥ 6 weeks, other CT for ≥ 4 weeks | P: safety, feasibility, dose-limiting toxicity S: immune response + correlation with intracranial T cell infiltration + clinical survival | - |
| Yajima 2005(87) | Japan | NR phase I | FT | R | GB or AA HLA-A24+ or HLA-A2+ | - | First surgery |
| Yamanaka 2005(88) | Japan | NR phase I/II | FT | R | Grade III or IV glioma Undergone surgery + RT + nitrosurea-based CT | Toxicity, immune response, clinical response | Diagnosis |
| Fakhrai 2006(89) | US | NR phase I | FT | R | Grade IV astrocytoma Completed surgery + RT Age ≥ 18 years KPS ≥ 60 | P: safety S: tumour progression, humoral + cellular immunity, nature of immune infiltrate in pre- + post-treatment biopsies | - |
| Ishikawa 2007(90) | Japan | NR phase I/II | FT | Mixed | GB Age 17-70 years | P: safety, feasibility S: OS, effect on remaining tumour | Diagnosis and therapy |
| Okada 2007(91) | US | NR phase I | FT | ND | Supratentorial GB or AA Age ≥ 18 years KPS ≥ 60 Tumour suitable for subtotal resection Off CST for 2 weeks prior to vaccination | P: safety | - |
| Izumoto 2008(92) | Japan | NR phase II | FT | Mixed | GB resistant to conventional CT + RT Age 16-80 years Expression of WT1 protein in glioma cells  HLA-A*2402 positivity  Expected survival > 3 months ECOG PS 0-2 | P: PFS S: OS | Initial vaccination |
| Wheeler 2008(93) | US | NR phase II | FT | Mixed | Malignant brain tumour KPS ≥ 60 Age ≥ 18 years CST free during blood collections + vaccinations | P: toxicity, OS, TTP | Surgery |
| Dillman 2009(94) | US | NR phase II | FT | ND | Completed primary therapy for GB without disease progression Age ≥ 16 years Life expectancy > 2 months KPS ≥ 60 Off CT/RT ≥ 4 weeks before treatment Fit for surgery | - | Diagnosis and therapy |
| Neyns 2009(95) | Belgium | NR phase II | FT | R | Supratentorial grade III or IV glioma EGFR gene copy number of glioma cells determined by FISH Progressive disease following surgery + RT + CT KPS ≥ 60 No RT or CT within past 4 weeks Stable CST dose for 2 weeks | P: ORR S: TTP, PFS, OS, safety | - |
| Sampson 2009(96) | US | NR phase I | FT | ND | GB Age ≥ 18 years KPS ≥ 80 | Feasibility, safety | Diagnosis and therapy |
| Ardon 2010(97) | Belgium | Pilot study | FT | ND | GB Age 18-70 years Total or subtotal resection confirmed by MRI within 72 hours post-operatively Tumour tissue available Perioperative CST tapered within 7 days Life expectancy > 3 months KPS > 70 | - | - |
| Clavreul 2010(98) | France | NR phase I | FT | R | Recurrent grade III or IV malignant glioma Fit for surgery Age 18-68 years KPS > 60 Off CST + CT 1 month before vaccination | Safety, feasibility | Second surgery |
| Iwadate 2010(99) | Japan | NR phase I/II | FT | ND | GB | P: OS S: PFS, safety | Surgery |
| Sampson 2010(100) | US | NR phase II | FT | ND | EGFRvIII-expressing GB GTR (≥ 95%) KPS ≥ 80 No radiological progression after RT + CT | P: PFS | Diagnosis |
| Fadul 2011(101) | Lebanon | NR phase I/II | FT | ND | GB Age ≥ 18 years Sufficient resected tumour Fit for surgery Completed 6 weeks RT + TMZ KPS ≥ 60 No CST within 2 weeks of leukapheresis | OS | Diagnosis |
| Muragaki 2011(102) | Japan | NR phase I/IIa | FT | ND | GB  Age 16–75 years Radiologically complete or subtotal resection Availability of at least 1.5 g of neoplastic tissue for AFTV preparation  Completed cumulative 60 Gy of RT KPS ≥ 60 | P: OS S: PFS, safety | Surgery |
| Prins 2011(103) | US | NR phase I | FT | Mixed | GB Amenable to surgical resection KPS ≥ 60 No CST within 10 days of vaccination | - | Diagnosis |
| Sampson 2011(104) | US | NR phase II | FT | ND | GB Adult KPS ≥ 80 Expression of EGFRvIII in tumour cells by IHC No radiological progression after RT | P: immune response S: PFS, OS | Diagnosis and therapy |
| Adair 2012(105) | US | Pilot study | FT | - | GB ≥ 50% initial surgical resection Unmethylated MGMT promoter | - | - |
| Ardon 2012(106) | Belgium | NR phase I/II | FT | ND | GB Age 18-70 years Total or subtotal resection confirmed by postoperative MRI within 72 hours Availability of > 3 cm^3^ tumour tissue CST tapered within 10 days postoperatively Life expectancy > 3 months KPS ≥ 70 | P: feasibility, PFS6 S: OS, immune profiling | Study entry |
| Cho 2012(107) | Taiwan | R phase II | FT | ND | GB Age 14-70 years KPS >70 ≥ subtotal resection | OS, PFS, QoL | - |
| Valle 2012(108) | Spain | Pilot study | FT | ND | GB | - | - |
| Crane 2013(109) | US | NR phase I/II | FT | R | Resectable grade III or IV glioma KPS ≥ 60 Life expectancy > 8 weeks Adequate vaccine production | P: safety, feasibility S: OS, immune response | - |
| D'Alessandris 2013(110) | Italy | NR phase I/II | FT | R | GB Normal PTEN status VEGF over-expression EGFRvIII expressing-tumour to receive erlotinib Age ≥ 18 years ≥ 4 weeks from surgery + CT/RT KPS ≥ 70 | P: ORR, PFS6, safety S: PFS, OS | Therapy |
| Pellegatta 2013(111) | Italy | NR phase I | FT | R | GB Life expectancy ≥ 3 months | - | Last surgery |
| Phuphanich 2013(112) | US | NR phase I | FT | ND | GB GTR ≥ 95% HLA-A1 or HLA-A2 positive Tumour positive for ≥ 1 vaccine antigen KPS ≥ 60 CST ≤ 4mg/day | P: immunogenicity S: safety, efficacy | - |
| Reardon 2013(113) | US | NR phase II | FT | R | Grade IV malignant glioma First or second recurrence  > 18 years ECOG 0 or 1 | P: PFS6, safety S: PFS, pharmacokinetics | - |
| Vik-Mo 2013(114) | Norway | NR phase I/II | FT | ND | GB Age 18-70 years Accessible volume + quality of tumour tissue ECOG PS 0 or 1 ≥ 4 weeks off CST before vaccination | P: adverse events S: PFS, OS, immune response | - |
| Adair 2014(115) | US | NR phase I/II | FT | ND | GB MGMT promoter unmethylated | - | - |
| Bloch 2014(116) | US | NR phase II | FT | R | GB Age ≥ 18 years KPS ≥ 70 Life expectancy ≥ 8 weeks >90% resection of contrast enhancing tumour | P: OS6 S: OS, PFS, safety, immune profiling | Surgery |
| Gallego 2014(117) | Spain | NR phase II | FT | R | GB  EGFR, EGFRvIII + PTEN positive immunohistochemistry Age >18 years Life expectancy > 8 weeks KPS ≥ 60 MRI within 14 days before treatment Stable CST dose for ≥ 5 days | P: ORR, PFS6 S: OS, toxicity | - |
| Hassler 2014(118) | Austria | NR phase I/II | FT | R | GB Immunohistochemical expression of ≥ 1 imatinib target: PDGFR-alpha, - beta, c-fms, c-kit, c-abl and arg kinase No neurosurgical or radiotherapeutic option Age ≥ 18 years WHO PS ≤ 2 Stable or decreasing CST dose for ≥ 1 week before therapy | P: OS S: PFS, PFS6, safety | Therapy |
| Ishikawa 2014(119) | Japan | NR phase I/IIa | FT | ND | GB Age 16–75 years KPS ≥ 60% Maximum possible resection Availability of at least 1.5 g of neoplastic tissue for AFTV preparation Cumulative dose of 60 Gy postoperative RT Lymphocyte count ≥ 1000 per mm3 before initiation of RT | P: OS S: PFS | Surgery |
| Olin 2014(120) | US | NR phase I | FT | R | GB, ependymoma or medulloblastoma Failed SOC therapy KPS ≥ 60 | P: safety S: immune response, TTP, OS | Therapy |
| Brown 2015(121) | US | NR phase I/II | FT | R | Grade III or IV glioma Adult Tumour not communicating with ventricles/CSF Expected survival > 3 months  KPS ≥ 70 Steroid independent Completed primary therapy  No restriction on IL13R-alpha-2 status | P: feasibility, safety | Recurrence |
| Hunn 2015(122) | New Zealand | NR phase I | FT | R | GB Relapsing tumour on MRI Previous TMZ + RT Age ≥ 18 ECOG PS ≤ 2 ≥ 3 cycles of adjuvant TMZ | P: feasibility, safety S: radiological response, PFS6, immune response, tolerability | Diagnosis |
| Lassman 2015(123) | US | NR phase II | FT | R | GB Age ≥ 18 years KPS ≥ 60 Prior treatment exclusively with surgery + RT + TMZ Activation or overexpression of ≥ 2 putative dasatinib targets in GB (ie, SRC, c-KIT, EPHA2, PDGFR) | P: PFS6, ORR | - |
| Mitchell 2015(124) | US | R phase I/II | FT | ND | GB  Adult GTR + residual contrast enhancement on MRI ≤ 1cm in diameter KPS ≥ 80 | - | - |
| Schijns 2015(125) | International | Pilot study | FT | R | Grade IV malignant glioma SOC treatment failure Operable tumour mass KPS > 60 | P: toxicity S: median OS, radiological response | Recurrence |
| Schuster 2015(126) | US | NR phase II | FT | ND | EGFRvIII-expressing GB Age ≥ 18 years Prior GTR + CT/RT | P: PFS S: OS, safety, immune response | Study entry |
| Akasaki 2016(127) | Japan | NR phase I/II | FT | Mixed | GB KPS ≥ 50 Age 20-75 years CST independent | P: OS S: PFS | Surgery |
| Batich 2017(128) | US | NR phase I | FT | ND | GB Adult GTR of > 90% with residual contrast enhancement of <1cm^2^ KPS ≥ 80 Dexamethasone ≤ 2mg/day | P: safety, feasibility | Diagnosis |
| Dunn-Pirio 2017(129) | US | NR phase I | A | R | Supratentorial GB First recurrence ≥ 8 weeks after RT | - | Therapy |
| Inoges 2017(130) | Spain | NR phase II | FT | ND | GB Complete or near complete resection Residual tumour volume <1cc on post-op imaging | P: PFS | - |
| Kong 2017(131) | South Korea | R open-label phase III | FT | ND | GB Age 18-70 years KPS ≥ 60 | P: PFS S: OS, ORR, DCR, QoL, KPS, adverse effects | Randomisation |
| Muragaki 2017(132) | Japan | R double-blind placebo-controlled phase IIb/III | A | ND | Supratentorial GB Adult | P: OS S: PFS, ORR, QOL, safety | - |
| O'Rourke 2017(133) | US | NR phase I | FT | R | EGFRvIII expressing GB Age ≥ 18 years ≤ 4mg dexamethasone/day ECOG PS 0 or 1 | P: safety, feasibility S: ORR, OS | Therapy |
| Sepúlveda-Sánchez 2017(134) | Spain | NR phase II | FT | R | GB EGFR amplification determined by FISH First recurrence Age > 18 years Progressive disease on MRI Completed Stupp protocol KPS ≥ 70 | P: PFS6 S: safety, tolerability, OS, antitumour response | - |
| van den Bent 2017(135) | International | NR phase I | FT | R | EGFR-amplified GB Adults Bevacizumab naïve | P: ORR S: PFS6, PFS, OS, safety, tolerability | - |
| Weller 2017(136) | International | R double-blind placebo-controlled phase III | FT | ND | GB expressing EGFRvIII Age ≥ 18 years Maximal surgical resection + RT + CT ECOG PS <3 CST ≤ 2mg dexamethasone daily | P: OS in patients with MRD | - |
| Buchroithner 2018(137) | Austria | R open-label phase II | FT | ND | Primary GB Age 18-70 years Resection of ≥ 70% of tumour | P: PFS12 S: OS, safety, toxicity | - |
| Hu 2018(138) | China | NR phase I | FT | R | MET-altered chemo-resistant secondary GB (MET-exon-14-skipping or PTPRZ1-MET fusion) Age ≥ 18 years Life expectancy ≥ 3 months KPS ≥ 50 Stable or decreasing CST dose | P: MTD S: PK/PD profile, ORR | - |
| Ji 2018(139) | China | NR phase I | FT | ND | Supratentorial GB Age 18-75 years ≥ 80% tumour resection KPS ≥ 70% Life expectancy to complete Stupp protocol | P: adverse events, PFS6 S: OS, PFS, immune response | - |
| Liau 2018(140) | US | R double-blind placebo-controlled phase III | FT | ND | GB Age 18-70 years KPS ≥ 70 Life expectancy ≥ 8 weeks Sufficient resected tumour to produce vaccine | P: PFS S: OS | Surgery |
| Pellegatta 2018(141) | Italy | NR phase II | FT | ND | GB No IDH1-2 mutations Age 18-70 years No multifocal or subependymal tumour diffusion <10ml residual tumour after surgery Dexamethasone ≤ 4mg/day KPS ≥ 70 | PFS12 | Surgery |
| Taylor 2018(142) | US | NR phase II | FT | R | GB Age ≥ 18 years ≤ 3 relapses Tumour RB1 positive by immunohistochemistry KPS ≥ 60 | P: PFS6 S: toxicity, OS, ORR | - |
| Yao 2018(143) | China | R double-blind placebo-controlled phase II | FT | Mixed | GB ≥ 95% tumour resection Age 17-70 years KPS ≥ 60 | P: PFS S: OS | - |
| D'Alessandris 2019(144) | Italy | Pilot study | A | R | GB | - | - |
| Du 2019(145) | China | NR phase II | FT | ND | Supratentorial GB EGFR positive Age 18-75 years >50% of gross tumour volume removed at surgery KPS ≥ 60 | P: OS, PFS S: ORR, toxicity | - |
| Goff 2019(146) | US | NR phase I | FT | Mixed | EGFRvIII positive GB Adult Radiological recurrence after initial surgical resection or CT/RT KPS ≥ 60 Stable CST dose | P: safety, PFS S: CAR persistence, radiological response | - |
| Hilf 2019(147) | International | NR phase I | FT | ND | GB Age ≥ 18 years KPS ≥ 70 Life expectancy > 6 months No prior therapy except surgery Sufficient tumour tissue + option to undergo leukapheresis Eligible for standard RT/CT Positive for HLA-A*02:01 or HLA- A*24:02 | P: safety, feasibility, biological activity | Diagnosis |
| Keskin 2019(148) | US | NR phase Ib | FT | ND | Supratentorial GB Age ≥ 18 years MGMT unmethylated KPS ≥ 70 Enhancing tumour ≤ 4cm in maximal diameter on post-operative imaging | P: safety, feasibility | Diagnosis |
| Lassman 2019(149) | International | R double-blind placebo-controlled phase III | A | ND | EGFR-amplified GB KPS ≥ 70 | P: OS | - |
| Lassman 2019(150) | International | NR phase I | FT | R | Adult with GB EGFR-amplification Bevacizumab, nitrosourea + EGFR-directed therapy naïve KPS ≥ 70 | - | Therapy |
| Migliorini 2019(151) | Switzerland | NR phase I/II | FT | ND | GB >18 years old HLA-A2 positive ≤ 4mg dexamethasone/day WHO PS <2 | P: safety, immunogenicity S: OS, PFS6, PFS9, vaccine specific peripheral CD4 and CD8 T cell responses | Diagnosis and study entry |
| Narita 2019(152) | Japan | R double-blind placebo-controlled phase III | FT | R | Supratentorial GB Refractory after SOC TMZ and RT HLA-A24 positive Age 18 -74 years Positive IgG responses to ≥ 2 of the 12 warehouse peptides in pre-vaccination plasma ECOG PS 0, 1 or 2 | P: OS S: 1 year survival rate, antitumour response, PFS, PFS6, peptide-specific IgG responses, cytotoxic T lymphocyte activity | Randomisation |
| Tien 2019(153) | US | NR phase 0 | FT | R | GB Intact RB expression CDKN2A deletion or CDK4/6 amplification | P: PD/PK profile S: PFS, OS | Repeat surgery |
| van Gool 2019(154) | Germany | Pilot study | A | Mixed | Primary GB | - | - |
| Wen 2019(155) | US | R double-blind placebo-controlled phase II | FT | ND | GB Age ≥ 18 years GTR or STR with <1cm^3^ of residual tumour KPS ≥ 70 HLA-A1+ or HLA-A2+ Completed 6 weeks RT + ≥ 50% TMZ protocol | P: OS S: PFS | Randomisation |
| Wen 2019(156) | US | NR phase II | FT | R | GB PI3K pathway activation ≥ 18 years Not responded to prior RT ≥ 12 weeks from RT completion Tumour progression confirmed on imaging KPS ≥ 60 No prior treatment with bevacizumab, VEGFi, PI3K, AKT or mTOR inhibitors | P: PI3K pathway inhibition in tumour tissue + PK profile in cohort 1 + PFS6 in cohort 2 | - |
| Chi 2020(157) | US | NR phase II | FT | R | GB Age ≥ 18 years Prescence of EGFR gene amplification by FISH Progressive disease by MacDonald criteria KPS ≥ 70 ≥ 12 weeks from RT + ≥ 2 weeks from resection | P: PFS6 S: safety, tolerability, PFS, OS, radiological response rate | - |
| Frenel 2020(158) | France | NR phase I | A | ND | GB Non-methylated MGMT gene promoter | P: tolerability, PD profile, efficacy | - |
| Kessler 2020(159) | Germany | Pilot study | FT | Mixed | Molecular analysis of tumour tissue consisting of at least methylation array + copy number variations ± additional gene panel sequencing Age ≥ 18 years IDH wildtype GB Neuropathological report of molecular analysis available for treating physicians Further treatment in the neuro-oncological department after report of molecular analysis | - | - |
| Lombardi 2020(160) | Italy | NR phase I | FT | R | High grade glioma Partial or complete loss of mismatch repair protein expression Age ≥ 18 years Relapse according to RANO criteria Failure of RT + TMZ No prior immunotherapy ECOG PS 0-2 Dexamethasone ≤ 4 mg/day | P: DCR S: PFS, OS, response duration, safety | Therapy |
| Mishinov 2020(161) | Russia | R phase II | FT | - | GB Age 18-70 years Maximum safe resection KPS ≥ 60 | - | Diagnosis and therapy |
| Reardon 2020(162) | US | R double-blind placebo-controlled phase II | FT | R | EGFRvIII-positive GB Bevacizumab + other VEGF/VEGF receptor-targeting agent naïve  Age ≥ 18 years First or second relapse Maximum feasible resection/biopsy + RT + TMZ KPS ≥ 70% Dexamethasone ≤ 4mg OD | P: PFS6 S: ORR, PFS, OS, EGFRvIII-specific humoral response, safety | - |
| Rudnick 2020(163) | US | NR phase I | FT | Mixed | Malignant glioma Age ≥ 18 years KPS ≥ 60 2 weeks off steroids before vaccine Normal haematological parameters | P: safety, immunogenicity, OS, PFS | Therapy |
| Sampson 2020(164) | US | NR phase IIb | A | R | De novo IL4R expressing GB No IDH1/2 mutation 1st or 2nd recurrence  Not eligible for resection tumour ≤ 4 cm KPS ≥ 70 | P: mOS S: impact of IL4R status on mOS | - |
| Smith 2020(165) | Australia | NR phase I | FT | Mixed | Primary GB Age ≥ 18 years ECOG performance status of 0, 1, or 2 at screening Life expectancy ≥ 6 months Histological diagnosis of primary GBM (WHO grade IV) CMV-positive serology or positive staining for CMV in tumour tissue | P: QoL, safety, feasibility | Diagnosis |
| van den Bent 2020(166) | International | R open-label phase II | FT | R | GB EGFR-amplified First recurrence after CT/RT with TMZ Age ≥ 18 years Relapsing > 3 months after RT No prior treatment with nitrosureas, bevacizumab or EGFR-targeting agents | P: OS in ITT population S: OS in EGFRvIII mutation subgroup, PFS, ORR | - |
| Wang 2020(167) | China | NR phase I | FT | Mixed | GB or lung cancer | P: safety  S: CD8+ and CD4+ T cell responses | Diagnosis |
| Bonneville-Levard 2021(168) | France | Prospective multicohort basket trial | FT | - | Metastatic solid or haematological tumour Pre-treated with ≥ 1 line of anti-cancer treatment | P: incidence of molecular alterations detected in tumour sample S: description of patients with actionable alterations with MBRT initiated + responses to MBRT | - |
| Cardona 2021(169) | Colombia | Retrospective analysis | FT | R | GB EGFR amplification + EGFRvIII mutation | PFS6, ORR, OS, safety | Therapy |
| Padovan 2021(170) | Italy | NR phase I/II | FT | R | GB EGFR-amplified First or subsequent disease progression after SOC One or more prior systemic therapies ECOG PS ≤ 2 or KPS ≥ 70 Age ≥ 18 years Evidence of disease progression on MRI | P: OS, safety S: PFS, DCR, ORR | Therapy |
| Werlenius 2021(171) | Sweden | R open-label phase II | FT | ND | GB Age 18-70 Fit for RT + TMZ WHO PS 0-2 No prior treatment Partial or complete resection | P: PFS S: survival, safety | Randomisation |
| Ciesielski 2022(172) | US | NR phase IIa | A | ND | GB  Age ≥ 18 KPS ≥70  IHC confirmation of survivin expression Expression of HLA-A*02, A*03, A*11 or A*24 MHC-I alleles Residual contrast enhancement of ≤1 cm^3^ by MRI within 72h post-resection | P: 70% PFS6 S: mPFS, mOS, safety, tolerability, immune response | - |
| Hu 2022(173) | US | NR phase I | FT | Mixed | GB  Age ≥18 years Cohort B were eligible up to third recurrence Prior RT and CT for cohort B <1 cm^2^ residual enhancing tumour after resection KPS ≥ 70 ≤ 4 mg dexamethasone daily dose | P: safety, tolerability S: PFS, OS, immune response | - |
| Kasenda 2022(174) | Switzerland | NR phase I | FT | R | GB EGFR amplified ECOG PS 0-2 Measurable disease by RANO criteria Recently obtained tumour specimen | P: concentration of anti-EGFR ILs-dox in plasma, CSF + glioblastoma tissue S: adverse events, tumour response, PFS, OS | Study registration |
| van Gool 2022(175) | Germany | Retrospective analysis | FT | ND | GB IDH1 wild-type  MGMT promoter-unmethylated | - | - |

**Supplementary Table 2.** Summary of patient characteristics for included studies. The percentage of studies reporting each data point is in brackets below the column headings. KPS = Karnofsky Performance Status, ORR = objective response rate, RPA = recursive partitioning analysis, MGMT = methylguanine methyltransferase, RT = radiotherapy, TMZ = temozolomide, IG = intervention group, CG = control group.

| Study | Number of Patients  (100%) | Subgroups  (37%) | Average Age  (98%) | Age range  (88%) | Average Baseline KPS (%)  (65%) | % Male  (89%) | % White Ethnicity  (10%) | % Complete Resection  (54%) | % MGMT Promoter Methylation  (44%) | % ORR  (37%) | Average time from diagnosis to therapy  (56%) | % RPA Class  (16%) |
| --- | --- | --- | --- | --- | --- | --- | --- | --- | --- | --- | --- | --- |
| Bloom 1973(74) | 13 | IG: 7 CG: 6 | Mode 51-60 | - | - | IG: 71 CG: 83 | - | - | - | - | ASAP | - |
| Merchant 1988(75) | 13 | - | All: 46 IG1: 45 IG2:49 | All: 16-66 IG1: 16-66 IG2: 38-62 | IG1: 68 IG2: 53 | 46 | - | - | - | - | - | - |
| Lillehei 1991(76) | 9 | - | 46 | 15-61 | 64 | 22 | - | - | - | - | - | - |
| Hayes 1995(77) | 33 | IG: 15 CG: 18 | 46 | 24-66 | 78 | 53 | - | - | - | 20 | Leukapheresis 8 days - 6 weeks after re-operation | - |
| Plautz 1998(78) | 9 | - | 46 | 16-71 | - | 56 | - | - | - | 22 | 7 weeks - 6 months | - |
| Schneider 2001(79) | 22 | IG: 11 CG: 11 | IG: 50 CG: 57 | IG: 30-67 CG: 47-66 | IG: 85 CG: 85 | 55 | - | IG: 45 CG: 45 | - | - | 10 weeks after surgery | - |
| Yu 2001(80) | 49 | IG: 7 CG: 42 | IG: 54 CG: 56 | 42-77 | - | IG: 43 CG: 50 | - | IG: 71 CG: 58 | - | - | - | - |
| Iwadate 2003(81) | 31 | - | 51 | 18-70 | 76 | 60 | - | 15 | - | 26 | Within 2 weeks of surgery | - |
| Yamanaka 2003(82) | 7 | - | 49 | 20-69 | 59 | 43 | - | - | - | 29 | Vaccination started when recurrence detected on imaging | - |
| Steiner 2004(83) | 110 | IG: 23 CG: 87 | IG: 50 CG: 55 | - | IG: 84 | IG: 70 CG: 64 | - | IG: 74 CG: 56 | - | - | 3-6 weeks after RT | - |
| Wheeler 2004(84) | 38 | IG1: 12 IG2: 13 CG: 13 | IG1: 53 IG2: 54 CG: 56 | - | - | IG1: 50 IG2: 77 CG: 38 | - | IG1: 100 IG2: 100 CG: < 100 | - | - | 15 weeks | - |
| Yu 2004(85) | 34 | IG: 8  CG: 26 | IG: 44  CG: 53 | - | - | - | - | IG: 50 CG: 58 | - | - | - | - |
| Liau 2005(86) | 111 | IG: 12 CG: 99 | 40 | 20-65 | 87 | 42 | - | - | - | 8 | 18 weeks | - |
| Yajima 2005(87) | 17 | - | 55 | 20-76 | - | 59 | - | - | - | 24 | - | - |
| Yamanaka 2005(88) | 45 | IG: 18 CG: 27 | IG: 50 CG: 56 | 20-80 | IG: 66 | IG: 66 | - | IG: 24 CG: 26 | - | 22 | At recurrence | - |
| Fakhrai 2006(89) | 6 | - | IG: 50 | 37-63 | - | IG: 66 | - | - | - | 33 | - | - |
| Ishikawa 2007(90) | 12 | - | 51 | 35-63 | 71 | IG: 66 | - | 0 | - | 33 | - | - |
| Okada 2007(91) | 5 | - | 53 | 45-61 | - | IG: 80 | - | 100 | - | - | 7-8 weeks | - |
| Izumoto 2008(92) | 21 | - | 51 | 20-76 | 77 | 67 | - | - | - | 10 | - | - |
| Wheeler 2008(93) | 34 | - | 52 | 22-74 | All: 82 R: 81 NR: 84 | 71 | - | 78 | - | - | 15 weeks after surgery | - |
| Dillman 2009(94) | 33 | - | 57 | 25-78 | ≥ 80 in 85% | 58 | - | 51 | - | - | 5.3 months | - |
| Neyns 2009(95) | 55 | IG1: 28 IG2: 27 | All: 53 IG1: 57 IG2: 50 | - | Mode 60-70 IG1 mode 80-90 IG2 mode 60-70 | All: 69 IG1: 64 IG2: 74 | All: 96 IG1: 96 IG2: 96 | All: 44 IG1: 54 IG2: 33 | - | All: 6 IG1: 7 IG2: 4 | - | - |
| Sampson 2009(96) | 12 | - | 44 | 34-58 | 96 | 67 | - | 100 | - | - | - | - |
| Ardon 2010(97) | 8 | - | 50 | 31-62 | 85 | 63 | - | 25 | - | - | - | III: 13  IV: 87 |
| Clavreul 2010(98) | 4 | - | 56 | 40-65 | 68 | - | - | - | - | 0 | - | - |
| Iwadate 2010(99) | 74 | - | 52 | 15-77 | ≥ 70 in 57% | 66 | - | 62 | 46 | - | - | - |
| Sampson 2010(100) | 35 | IG:18 CG: 17 | IG: 52 CG: 59 | IG: 29-67 CG: 37-71 | IG: 92 | IG: 72 CG: 47 | - | IG: 100 CG: 100 | IG: 39 | - | - | - |
| Fadul 2011(101) | 10 | - | 61 | 48-78 | 80 | 50 | - | 10 | - | - | Leukapheresis 3-7 weeks after RT | - |
| Muragaki 2011(102) | 22 | - | 58 | 18-70 | 90 | 68 | - | 73 | - | - | AFTV commenced once RT reached 32-36 Gy | III: 32 IV: 36 V: 32 |
| Prins 2011(103) | 23 | - | 51 | 26-74 | 83 | 70 | - | - | - | - | 7-30 weeks after surgery | - |
| Sampson 2011(104) | 22 | IG1: 12 IG2: 10 | 57 | 41-83 | 96 | - | - | 100 | 32 | - | - | III 18  IV 82 |
| Adair 2012(105) | 3 | - | 53 | 51-56 | 97 | - | - | 67 | 0 | - | - | - |
| Ardon 2012(106) | 77 | - | 57 | 26-70 | - | 62 | - | 66 | 38 | - | Leukapheresis within 2-5 weeks of diagnosis | III: 17 IV: 65 V: 18 |
| Cho 2012(107) | 34 | IG: 18 CG: 16 | IG: 58 CG: 59 | IG: 32-68 CG: 36-69 | IG: 86 CG: 88 | IG: 44 CG: 50 | - | IG: 78 CG: 69 | IG: 56 CG: 56 | - | 1-2 months after surgery | III: IG 17 CG 13  IV: IG 55 CG 75 V: IG 28 CG 13 |
| Valle 2012(108) | 5 | - | 66 | 50-73 | 70 | 40 | - | 60 | 80 | - | Leukapheresis within 2 weeks of surgery | IV: 20 V: 80 |
| Crane 2013(109) | 12 | - | 52 | 36-73 | - | - | - | - | - | - | - | - |
| D'Alessandris 2013(110) | 10 | IG1: 6 IG2: 4 | 53 | 30-77 | - | All: 90 IG1: 100 IG2: 75 | - | - | 40 | All: 70 IG1: 50 IG2: 100 | - | - |
| Pellegatta 2013(111) | 15 | - | 47 | 17-64 | 70 | 40 | - | 7 | - | - | Leukapheresis within 4 weeks of surgery | - |
| Phuphanich 2013(112) | 16 | - | 55 | 34-79 | 87 | 75 | - | 75 | 38 | - | 4.5 months | - |
| Reardon 2013(113) | 37 | P: 19 N: 18 | P: 56 N: 50 | P: 42-73 N: 20-76 | - | P: 74 N: 77 | - | - | - | P: ORR 5 N: ORR 5 | - | - |
| Vik-Mo 2013(114) | 84 | IG: 7 CG: 77 | IG: 56 CG: 56 | IG: 46-63 | - | IG: 57 | - | - | - | - | - | III: 29 IV 71 |
| Adair 2014(115) | 7 | - | 55 | 41-64 | 96 | 57 | - | 86 | 0 | 14 | - | III: 14 IV: 71 V: 14 |
| Bloch 2014(116) | 41 | - | 55 | 21-75 | 82 | 73 | 95 | 100 | - | - | 31 days after surgery | - |
| Gallego 2014(117) | 13 | - | 53 | - | 80 | 46 | - | - | - | 8 | - | - |
| Hassler 2014(118) | 24 | - | 53 | 18-72 | - | 55 | - | 42 | - | 17 | 10.5 months | - |
| Ishikawa 2014(119) | 24 | - | 48 | 26-66 | 80 | 71 | - | 67 | - | - | 4 weeks after RT | III 33 IV 42 V 25 |
| Olin 2014(120) | 5 | - | 53 | 17-71 | 84 | 80 | - | - | - | 0 | - | - |
| Brown 2015(121) | 3 | - | 50 | 36-57 | 90 | 33 | - | 100 | - | - | - | - |
| Hunn 2015(122) | 9 | - | 44 | 30-65 | - | 44 | - | 22 | - | 22 | 3 weeks after surgery | III: 100 |
| Lassman 2015(123) | 50 | - | IG1: 51 IG2: 54 | IG1: 33-81 IG2: 26-75 | 60-80: IG1 71% IG2 38% 90-100: IG1 29% IG2 62% | IG1: 48 IG2: 59 | IG1: 95 IG2: 90 | IG1: 43 IG2: 62 | - | 0 | - | - |
| Mitchell 2015(124) | 12 | IG1: 6 IG2: 6 | IG1: 65 IG2: 59 | IG1: 30-75 IG2: 28-66 | IG1: 90 IG2: 90 | IG1: 50 IG2: 33 | 83 | 100 | IG1: 50 IG2: 33 | - | - | IG1: median IV IG2: median IV |
| Schijns 2015(125) | 9 | - | 48 | 27-65 | 80 | 44 | - | - | - | 22 | 10 days after surgery | - |
| Schuster 2015(126) | 65 | - | 56 | 30-83 | 90 | 51 | - | 100 | 38 | - | 3 months after study entry | - |
| Akasaki 2016(127) | 32 | - | All 55 IG1: 50 IG2: 57 | All: 30-74 IG1: 35-65 IG2: 30-74 | - | All: 56 IG1: 50 IG2: 59 | - | - | - | - | When general condition permitted | - |
| Batich 2017(128) | 11 | - | 55 | 47-67 | 96 | 73 | 91 | 100 | 45 | - | 4 weeks following RT/TMZ | III 9 IV 91 |
| Dunn-Pirio 2017(129) | 21 | - | - | - | - | - | - | - | - | - | - | - |
| Inoges 2017(130) | 31 | - | 59 | 42-70 | 79 | 52 | - | 81 | 45 | - | 23 days | III 10 IV 42 V 48 |
| Kong 2017(131) | 180 | IG: 91 CG: 89 | IG: 53 CG: 53 | IG: 19-69 CG: 23-68 | IG: 84 CG: 86 | IG: 56 CG: 57 | - | IG: 48 CG: 54 | - | IG: 27 CG: 16 | IG: 21 days CG: 22 days | - |
| Muragaki 2017(132) | 60 | - | 61 | - | - | 55 | - | - | - | - | - | - |
| O'Rourke 2017(133) | 10 | - | 60 | 45-76 | Mode 80-90 | 50 | - | - | 0 | - | 358 days | - |
| Sepúlveda-Sánchez 2017(134) | 49 | IG1: 30 IG2: 19 | All: 59 IG1: 63 IG2: 52 | All: 39-81 IG1: 41-81 IG2: 39-72 | - | All 65 IG1: 67 IG2: 63 | - | - | All: 20 IG1: 30 IG2: 5 | All: 6 IG1: 7 IG2: 5 | At least 12 weeks after RT | - |
| van den Bent 2017(135) | 66 | - | 58 | 35-80 | 86 | 59 | - | - | 8 | 7 | - | - |
| Weller 2017(136) | 745 | All: 745 (405 MRD) IG: 371 (195 MRD) CG: 374 (210 MRD) | IG: 59 CG: 57 | IG: 51-64 CG: 51-64 | - | IG: 68 CG: 58 | IG: 90 CG: 92 | - | IG: 35 CG: 35 | - | 2.8 months from diagnosis to randomisation | III: IG 13 CG 13 IV: IG 71 CG 75 V: IG 16 CG 12 |
| Buchroithner 2018(137) | 76 | IG: 34 CG: 42 | All: 54 IG: 55 CG: 54 | 19-70 | - | All: 67 IG: 65 CG: 69 | - | IG: 71 CG: 83 | IG: 35 CG: 35 | - | 7 weeks | - |
| Hu 2018(138) | 6 | - | 39 | 31-53 | 77 | 67 | - | 50 | 67 | 33 | - | - |
| Ji 2018(139) | 20 | - | 52 | 40-70 | 80 | 50 | - | 100 | 10 | - | 98 days | - |
| Liau 2018(140) | 331 | IG: 232 CG: 99 | 55 | 19-73 | 90 | 61 | 89 | 63 | 40 | - | - | - |
| Pellegatta 2018(141) | 24 | - | 54 | 23-70 | 84 | 67 | - | - | 25 | - | 9 weeks after surgery | - |
| Taylor 2018(142) | 22 | IG1: 6 IG2: 16 | Median All: 48 IG1: 50 IG2: 48 | All: 23-78 IG1: 31-66 IG2: 23-78 | All: 90 IG1: 85 IG2: 90 | All: 55 IG1: 50 IG2: 56 | All: 91 IG1: 83 IG2: 88 | All: 9 IG1: 0 IG2: 13 | - | - | IG1: palbociclib for 7 days before surgery and resumed at least 2 weeks post-operatively | - |
| Yao 2018(143) | 43 | IG: 22 CG: 21 | IG: 48 CG: 50 | IG: 25-71 CG: 22-71 | IG: 83 CG: 79 | IG: 59 CG: 52 | - | 100 | IG: 41 CG: 57 | - | 4 weeks | - |
| D'Alessandris 2019(144) | 34 | IG1: 16 IG2: 14 IG3: 4 | - | - | - | - | - | - | - | All: 50 IG1: 38 IG2: 57 IG3: 75 | - | - |
| Du 2019(145) | 36 | - | 49 | 18-69 | 90-100: 69% 60-80: 31% | 53 | - | 42 | 44 | 72 | - | - |
| Goff 2019(146) | 18 | - | 54 | 43-66 | - | 83 | - | - | 22 | 0 | 11.1 months | - |
| Hilf 2019(147) | 15 | - | 53 | 25-70 | 90 | 56 | - | 100 | 29 | 27 | 52 days | - |
| Keskin 2019(148) | 8 | - | 65 | 45-73 | 86 | 25 | - | 37 | 0 | - | 20 weeks from surgery | III 13 IV 25 V 63 |
| Lassman 2019(149) | 639 | - | 60 | 22-84 | - | 62 | - | - | - | - | - | - |
| Lassman 2019(150) | 60 | - | 56 | 20-79 | 86 | 58 | - | - | 18 | 14 | - | - |
| Migliorini 2019(151) | 16 | - | 58 | 41-73 | - | 81 | - | 38 | 25 | 19 | 8 weeks from RT/TMZ initiation | - |
| Narita 2019(152) | 88 | IG: 58 CG: 30 | IG: 53 CG: 59 | IG: 20-74 CG: 32-74 | IG: ≤ 80 in 74% CG: ≤ 80 in 77% | IG: 64 CG: 63 | - | - | - | - | IG: 12 months CG: 13 months | - |
| Tien 2019(153) | 6 | - | 49 | 31-66 | - | 67 | - | 67 | - | - | - | - |
| van Gool 2019(154) | 132 | IG1: 71 IG2: 61 | IG1: 55 IG2: 53 | - | IG1: 90 IG2: 80 | - | - | - | - | - | - | - |
| Wen 2019(155) | 124 | IG: 81 CG: 43 | All: 57 IG: 57 CG: 58 | 23-82 | 90 | All: 60 IG: 54 CG: 72 | - | All: 73 IG: 72 CG: 74 | All: 37 IG: 34 CG: 42 | - | 1 week after completion of RT/CT | - |
| Wen 2019(156) | 65 | IG1: 15 IG2: 50 | IG1: 55 IG2: 56 | IG1: 39-68 IG2: 29-80 | IG1: 89 IG2: 88 | IG1: 73 IG2: 74 | - | - | - | IG1: 0 IG2: 0 | - | - |
| Chi 2020(157) | 56 | IG1: 10 IG2: 30 IG3: 16 | All: 60 IG1: 58 IG2: 61 IG3: 60 | All: 35-81 IG1: 47-74 IG2: 42-77 IG3: 35-81 | All: 85 IG1: 90 IG2: 90 IG3: 70 | All: 57 IG1: 50 IG2: 50 IG3: 75 | All: 91 IG1: 90 IG2: 97 IG3: 81 | - | IG1: 1 IG2: 33 IG3: 31 | IG1: 0 IG2: 3 IG3: 0 | All: 11 months IG1: 11.5 months IG2: 11 months IG3: 12 months | - |
| Frenel 2020(158) | 23 | - | 56 | 42-73 | - | - | - | - | 0 | - | - | - |
| Kessler 2020(159) | 253 | Molecularly guided therapy:97 Therapy not molecularly guided:156 | 61 | 21-85 | 80 | 57 | - | - | 46 | - | 30 days after diagnosis in first-line therapy and 600 days in recurrent disease | - |
| Lombardi 2020(160) | 8 | - | 47 | 21-65 | - | 63 | - | - | 50 | 0 | - | - |
| Mishinov 2020(161) | 58 | IG1: 18 IG2: 9 CG: 31 | IG1: 53 IG2: 52 CG: 56 | 18-70 | IG1: 60 IG2: 70 CG: 60 | All: 52 IG1: 56 IG2: 22 CG: 58 | - | - | - | - | - | - |
| Reardon 2020(162) | 73 | IG: 36 CG: 37 | IG: 59 CG: 55 | IG: 44-79 CG: 30-75 | IG: 83 CG: 84 | IG: 53 CG: 59 | - | IG: 39 CG: 16 | - | IG: 30 CG: 18 | IG: 10.8 months CG: 11.6 months | - |
| Rudnick 2020(163) | 23 | IG1: 8 IG2: 15 | All: 55 IG1: 61 IG2: 52 | All: 30-72 IG1: 42-72 IG2: 30-66 | All: 88 IG1: 88 IG2: 88 | All: 39 IG1: 0 IG2: 60 | - | All: 52 IG1: 88 IG2: 33 | - | All: 25 IG2: 33 | 5 weeks after surgery | - |
| Sampson 2020(164) | 44 | - | 56 | 35-77 | 50% ≤ 80 | - | - | - | 48 | - | - | - |
| Smith 2020(165) | 25 | - | 59 | 32-74 | - | 64 | - | - | 39 | - | 162 days after diagnosis and 56 days after trial recruitment | - |
| van den Bent 2020(166) | 260 | IG1: 88 IG2: 86 IG3: 86 | All: 59 IG1: 59 IG2: 58 IG3: 59 | All: 35-82 IG1: 40-75 IG2: 36-79 IG3: 35-82 | - | All: 64 IG1: 67 IG2: 58 IG3: 67 | - | - | All: 48 IG1: 49 IG2: 48 IG3: 49 | - | All: 6 weeks IG1: 6 weeks IG2: 5.8 weeks IG3: 6.2 weeks | - |
| Wang 2020(167) | 5 | - | IG: 37 CG: 49 | IG: 18-55 CG: 22-67 | - | IG: 80 CG: 75 | - | - | - | - | Within 7 days of surgery | - |
| Bonneville-Levard 2021(168) | 6 | - | 56 | 45-73 | - | - | - | - | - | - | 27 months | - |
| Cardona 2021(169) | 15 | - | 56 | 38-70 | All >70 | 67 | - | 53 | 47 | 13 |  | V 93  VI 7 |
| Padovan 2021(170) | 36 | - | 57 | 38-73 | - | 69 | - | - | 64 | 14 | 81% > 16 weeks after last TMZ cycle | - |
| Werlenius 2021(171) | 62 | IG: 40 CG: 22 | All: 57 IG: 56 CG: 62 | 38-69 | - | All: 59 IG: 60 CG: 57 | - | All: 44 IG: 43 CG: 48 | All: 43 IG: 67 CG: 30 | - | All: 32 days IG: 31 days CG: 35 days | - |
| Ciesielski 2022(172) | 63 | - | 60 | - | - | 60 | - | - | 52 | - | - | - |
| Hu 2022(173) | 36 | IG1: 11 IG2: 25 | IG1: 56 IG2: 52 | - | IG1: 85 IG2: 80 | IG1: 73 IG2: 56 | IG1: 91 IG2: 31 | IG1: 100 IG2: 92 | IG1: 100 IG2: 31 | - | IG1: 1 week after completion of RT/CT IG2: As soon as vaccine manufactured, typically within 3 weeks of apheresis | - |
| Kasenda 2022(174) | 9 | - | 59 | 44-64 | - | 78 | - | - | 44 | - | - | - |
| van Gool 2022(175) | 32 | IG1: 7 IG2: 25 | All: 47 IG1: 49 IG2: 46 | 18-69 | All: 85 IG1: 70 IG2: 90 | All: 63 IG1: 29 IG2: 72 | - | 34 | 0 | - | - | - |

**Supplementary Table 3.** Personalised therapies and survival reported in included studies. Median values are in regular font; mean values are in italics. FT = from therapy, FD = from diagnosis, IG = intervention group, (H)CG = (historical) control group, RT = radiotherapy, CT = chemotherapy, TMZ = temozolomide, PPV = personalised peptide vaccine, SC = subcutaneous, IV = intravenous, ATL = autologous tumour lysate, DC = dendritic cell, LAK = lymphokine activated killer, TTP = time to progression, OS = overall survival, PFS = progression free survival.

| Study | Personalised Therapy | Therapy Details | Median PFS | Median OS | Additional Survival Outcomes | Statistical Analysis | Interpretation |
| --- | --- | --- | --- | --- | --- | --- | --- |
| Bloom 1973(74) | Autologous tumour cell vaccine | IG: surgery + RT + up to 3 SC vaccinations CG: surgery + RT | - | IG: 7 months CG: 7 months | - | No significant survival difference between IG and CG | Associated with no survival benefit |
| Merchant 1988(75) | Autologous LAK cells + rIL-2 | IG1: surgery + 1 perilesional injection + intracavitary injections via Ommaya reservoir IG2: surgery + intracavitary injections At least 2 cycles of injections over 4-6 weeks Prior therapy: surgery + RT +/- CT | - | *All: 30 weeks IG1: 30 weeks IG2: 33 weeks* | - | - | Appears unbeneficial |
| Lillehei 1991(76) | Autologous LAK cells + rIL2-activated autologous lymphocytes | Surgery + intracavitary autologous LAK cells + autologous stimulated lymphocytes + rIL2 plasma clot  After 1 month, intracavitary autologous stimulated lymphocytes + rIL2 via Rickman reservoir No CG | *43 weeks TTP* | *20 weeks FT 63 weeks FD* | - | - | Appears unbeneficial |
| Hayes 1995(77) | Autologous LAK cells + rIL2 | Intracavitary autologous LAK cells + rIL2 via Ommaya reservoir. LAK cells + rIL2 on day 1 + rIL2 alone 5 times during 2-week cycle. Cycle repeated at 2 weeks. Each 2-cycle course repeated at 3-month intervals if response or stable disease HCG: surgery + CT | - | IG: 53 weeks CG: 26 weeks | - | Significantly longer survival in IG than CG (*p* = .004, non-paired t test) | Associated with survival benefit |
| Plautz 1998(78) | Autologous tumour cell vaccine + T cell adoptive immunotherapy | Intradermal vaccine + GM-CSF adjuvant. Cells from draining LNs resected 7 days after vaccination and stimulated *in vitro*. Activated CD4+ and CD8+ T cells subsequently administered via IV infusion. No CG | - | *11 months* | - | - | Appears unbeneficial |
| Schneider 2001(79) | Autologous tumour cell vaccine | IG: surgery + RT + vaccination 4 times within 2-week interval and at 3 months CG: surgery + RT + CT | - | IG: 46 weeks CG: 48 weeks | - | No significant survival difference between IG and CG (*p* = .17, log-rank test) | Associated with no survival benefit |
| Yu 2001(80) | Vaccination with autologous DCs pulsed with peptides eluted from the surface of autologous tumour cells | 3 biweekly intradermal vaccinations in deltoid region | - | IG: 455 days CG: 257 days | - | - | Appears beneficial |
| Iwadate 2003(81) | Personalised chemotherapy | Drug sensitivity testing of resected tumour cells to 30 agents by flow cytometric detection of apoptosis. Best *in vitro* regimen given ≥ every 3 months for 1 year + RT  No CG | - | 20.5 months | OS36: 10% | - | Appears beneficial |
| Yamanaka 2003(82) | ATL/DC vaccine | Mean 4 intradermal doses close to cervical lymph nodes + 3 intratumoural doses via Ommaya reservoir | - | *174 weeks* | - | - | Appears beneficial |
| Steiner 2004(83) | Autologous tumour cell vaccine | IG: surgery + RT + vaccine of autologous tumour cells infected with Newcastle Disease Virus. Up to 8 intradermal vaccinations at 3–4-week intervals CG: surgery + RT | IG: 40 weeks CG: 26 weeks | IG: 100 weeks CG: 49 weeks | OS12: IG 91% CG 45% OS24: IG 39% CG 11% OS36+: IG 4% CG 0% | Significantly longer PFS (*p* = .024, log-rank test) and OS (*p* < .001, log-rank test) in IG than CG | Associated with survival benefit |
| Wheeler 2004(11) | Vaccination with autologous DCs pulsed with HLA-eluted peptides from cultured tumour cells or ATL | IG1: vaccine IG2: vaccine + CT CG: CT 3 vaccines given 2 weeks apart | - | *IG1: 17.9 months IG2: 26 months CG: 15.9 months* | OS24: IG1 8% IG2 42% CG 8% OS36: IG1 0% IG2 18% CG 0% | Significantly longer survival in vaccinated patients receiving CT compared to patients receiving isolated vaccination or CT (*p* = .047, log-rank test) | Associated with survival benefit |
| Yu 2004(12) | ATL/DC vaccine | IG: surgery + 3 vaccinations at 2-week intervals CG: surgery | - | IG: 133 weeks CG: 30 weeks | - | Significantly longer survival in IG than CG (*p* = .0013, log-rank test) | Associated with survival benefit |
| Liau 2005(13) | Vaccination with autologous DCs pulsed with autologous tumour peptides | IG: surgery + RT + 3 biweekly intradermal vaccinations CG: surgery + RT | 15.5 months TTP | 23.4 months | OS6: 100% OS12: 75% OS24: 50% OS48+: 17% | Significant longer OS (*p* = .006) and TTP (*p* = .028) in IG than CG | Associated with survival benefit |
| Yajima 2005(14) | Personalised peptide vaccine | Surgery + RT + CT + PPV Biweekly administration of 3mg peptide to a maximum of 4 peptides or weekly administration of 2mg peptide to a maximum of 4 peptides | 93 days TTP biweekly protocol 133 days TTP weekly protocol | 622 days | OS12: 82% OS24: 41% | - | Appears beneficial |
| Yamanaka 2005(15) | ATL/DC vaccine | IG: surgery + RT + CT + intradermal +/- intratumoural vaccine every 3 weeks CG: surgery + RT + CT | - | IG: 480 days CG: 400 days | OS24: IG 24% CG 4% | Significant longer OS in IG than in CG (*p* = .010, log-rank test)  Significantly longer survival when DCs matured with OK-432 (*p* = .027), following intratumoral + intradermal administration (*p* = .042), with delayed-type hypersensitivity response (*p* = .003) + with tumour-lysate reactive CD8+ T cells after vaccination (*p* = .015) | Associated with survival benefit |
| Fakhrai 2006(16) | Autologous tumour cell vaccine | Surgery + RT + 4 SC injections of irradiated TGF-beta2 antisense gene-modified autologous tumour cells at monthly intervals. Further injections at escalating dose in responding patients No CG | - | IG: 68 weeks | - | - | Appears beneficial |
| Ishikawa 2007(17) | Autologous formalin-fixed tumour vaccine | Surgery + RT + CT + three five-site intradermal vaccinations into upper arm at weekly intervals No CG | - | 10.7 months FT 24.0 months FD | mOS FT: R 20.3 months NR 5.0 months | Significantly longer OS in responders than non-responders (*p* = .004, log-rank test) | Appears beneficial |
| Okada 2007(18) | Vaccination with autologous fibroblasts transfected with TGF-IL-4 neo-TK and admixed with ATL/DCs | Surgery + RT + 2 intradermal vaccinations No CG | 6 months TTP | - | - | - | Appears unbeneficial |
| Izumoto 2008(19) | Wilms tumour 1 peptide vaccination | Surgery + RT + CT + weekly intradermal vaccinations for 12 consecutive weeks then continued weekly in responders until progression No CG | 20 weeks | 36.7 weeks | PFS6: 33% | Longer PFS in group with greater WT1 protein expression level (*p* = .002, log-rank test) | Appears beneficial |
| Wheeler 2008(20) | ATL/DC vaccine | Surgery + RT +/-CT + 4 vaccinations No CG | Responders: 308 days TTP Non-responders: 167 days TTP | *Responders: 642 days Non-responders 430 days* | OS12: R 82% NR 67% OS24: R 41% NR 7% | Significantly longer TTP (*p* = .015, log-rank test) and OS (*p* = .041, log-rank test) in responders than non-responders | Appears beneficial |
| Dillman 2009(21) | Autologous LAK cells | Surgery + RT +/- CT + single instillation of intracavitary LAK cells No CG | - | 14.5 months FT 20.5 months FD | OS12: 75% OS24: 35% | Longer survival in patients who received higher number of T-LAK cells (*p* = .036, log-rank test), which was associated with not taking CST in the months preceding leukapheresis | Appears beneficial |
| Neyns 2009(22) | Cetuximab (EGFRi mAb) | Surgery + weekly IV cetuximab + RT + CT IG1: EGFR gene amplified (A)  IG2: EGFR gene non-amplified (NA) | All: 1.9 months IG1: 1.9 months  IG2: 1.8 months | All: 5.1 months IG1: 5.0 months IG2: 4.8 months | PFS6: All 7% IG1 7% IG2 11% OS6: All 38% IG1 43% IG2 33% | No significant difference in PFS or OS between IG1 and IG2 No significant correlation between response, survival and EGFR amplification | Associated with no survival benefit |
| Sampson 2009(23) | Autologous DC vaccine pulsed with rindopepimut (peptide with homology to EGFRvIII) | Surgery + RT + 3 intradermal vaccinations No CG | 6.8 months TTP FT 10.2 months TTP FD | 18.7 months FT 22.8 months FD | - | - | Appears beneficial |
| Ardon 2010(24) | ATL/DC vaccine | Surgery + RT + TMZ + 4 vaccinations at weekly intervals + then on day 8 of 1st, 2nd, 3rd and 6th adjuvant TMZ cycles | 18 months | 24 months | PFS6: 75% | - | Appears beneficial |
| Clavreul 2010(25) | Autologous tumour cell vaccine | Surgery + RT +/- CT + 4 vaccinations at 7-day intervals + continuous GM-CSF infusion for 28 days or 4 vaccinations at 21-day intervals with GM-CSF infusion for 3 days before and 14 days after each vaccination | - | *50 weeks* | - | - | Appears beneficial |
| Iwadate 2010(26) | Personalised chemotherapy | Surgery + RT + individualised CT using *in vitro* testing of 25 drugs on cell suspensions from surgical resections. Most effective drug + additional 1 or 2 drugs selected for combination according to effectiveness and mechanism of action.  No CG | 9.2 months PFS &TTP | 19.4 months | OS24: 37% | Patients with negative MGMT immunostaining had significantly longer survival than those with positive MGMT immunostaining (mOS 22.3 months vs 15.1 months, *p* = .0188, log-rank test) | Appears beneficial |
| Sampson 2010(27) | Rindopepimut (EGFRvIII peptide vaccine) | IG: surgery + RT + TMZ + intradermal injections, initially 3 at 2 weekly intervals, then monthly until toxicity or progression CG: surgery + RT + TMZ | IG: 14.2 months CG: 6.3 months | IG: 26 months CG: 15 months | PFS6: FT 67% FD 94% | OS (HR 5.3, *p* = .0013) and PFS (HR 2.2, *p* = .041) greater than CG matched for eligibility criteria, prognostic factors and temozolomide treatment after adjustment for age and KPS. Groups with specific antibody (*p* = .025) or delayed-type hypersensitivity (*p* = .03) responses to EGFRvIII had significantly longer OS. | Associated with survival benefit |
| Fadul 2011(28) | ATL/DC vaccine | IG: surgery + RT + TMZ + 3 vaccinations at 2 weekly intervals bilaterally into cervical lymph nodes No CG | 9.5 months | 28 months | PFS6: 90% | OS significantly longer in the 50% with the strongest immune responses to vaccination (*p* = .002, log-rank test) | Associated with survival benefit |
| Muragaki 2011(29) | Autologous formalin-fixed tumour vaccine | Surgery + RT + 3 courses of 5 intradermal vaccinations at weekly intervals once RT dose reached 32-36 Gy | 7.6 months | 19.8 months | OS24: 40% | PFS significantly longer in patients with a delayed-type hypersensitivity response of ≥ 12 mm (13.9 months vs 4.3 months, *p* < .001). PFS associated with p53 staining index (*p* < .05) | Appears beneficial |
| Prins 2011(30) | ATL/DC vaccine + imiquimod (TLR agonist) | IG: surgery + RT + CT + 3 biweekly intradermal ATL/DC vaccinations followed by booster vaccinations with either imiquimod or poly-ICLC adjuvant every 3 months until progression | 15.9 months TTP | All: 31.4 months ND: 35.9 months R: 17.9 months | OS12: All 91% ND 93% OS24: All 55% ND 77% OS36: All 47% ND 58% | OS was significantly longer for those who received DC vaccination at initial diagnosis than at recurrence (*p* = .03)  Patients with the proneural gene expression signatures had indistinguishable OS from 60 controls (*p* = .664). Patients with mesenchymal gene expression signatures had a significantly extended survival compared with 82 controls (*p* = .0046) | Appears beneficial |
| Sampson 2011(31) | Rindopepimut (EGFRvIII peptide vaccine) | IG1: surgery + RT + TMZ + vaccination + standard dose TMZ IG2: surgery + RT + TMZ + vaccination + dose intensified TMZ 3 vaccinations at biweekly interval within 6 weeks of RT completion. Subsequent vaccines on day 21 of 28-day cycle until progression or death | 15.2 months FD 11.8 months FT | 23.6 months FD 19.3 months FT | PFS6: IG1 75% IG2 90% OS12: IG1 83% IG2 90%  mOS FD: IG1 21 months IG2 >18.9 months mOS FT: IG1 17.4 months IG2 >15.7 months  mPFS FD: IG1 15.9 months IG2 14.9 months mPFS FT: IG1 12.1 months IG2 11.6 months | Median PFS (15.2 months vs 6.3 months, HR 0.35, *p* = .024) and OS (23.6 months vs 15 months, HR 0.23, *p* =.019) exceeded historical controls matched for entry criteria and adjusted for prognostic factors | Associated with survival benefit |
| Adair 2012(32) | Infusion of autologous P140K-modified haematopoietic stem and progenitor cells | Surgery + RT + CT + HSC infusion No CG | - | - | OS12: 100% | - | Associated with survival benefit |
| Ardon 2012(33) | ATL/DC vaccine | Surgery + RT + TMZ + 4 intradermal induction vaccinations at weekly intervals and 4 boost vaccines given during maintenance CT | 10.4 months | 18.3 months | PFS6: 70% | Patients with a methylated MGMT promoter had significantly better PFS *(p* = .0027) and OS *(p* = .0082). | Appears beneficial |
| Cho 2012(34) | ATL/DC vaccine | IG: surgery + RT + CT + 10 subaxillary SC vaccinations over 6 months CG: surgery + RT + CT | IG: 8.5 months CG: 8.0 months | IG: 31.9 months CG: 15.0 months | OS12: IG 89% CG 75% OS24: IG 44% CG 19% OS36: IG 17% CG 0% | Significantly longer OS in IG than CG (*p* < .002, Wilcoxon rank sum test) | Associated with survival benefit |
| Valle 2012(35) | ATL/DC vaccine | Fluorescence-guided surgery + RT + CT + vaccination at reducing frequency until end of all available doses. During TMZ treatment, vaccine given on day 21 of cycle | 16.1 months | 27 months | - | OS significantly longer than in HCG (median 31.2 vs 11.5 months, *p* = .02, log-rank test) Expected mOS in a group with these prognostic factors calculated to be 12.3 months. OS exceeded this by 14.7 months. | Associated with survival benefit |
| Crane 2013(36) | Autologous HSPPC-96 vaccine | Vaccine every 1-2 weeks for first 4 vaccinations then 2 weekly Prior therapy: surgery + RT + CT | - | 47 weeks in 11 immune-responders 16 weeks in 1 non-responder | - | - | Appears unbeneficial |
| D'Alessandris 2013(37) | Bevacizumab (VEGFi) + erlotinib (EGFRi) | IG1: bevacizumab  IG2: bevacizumab + erlotinib Prior therapy: surgery + RT + TMZ | All: 8.0 months IG1: 5.5 months IG2: 10.5 months | All: 9.5 months IG1: 6.8 months IG2: 17.0 months | PFS6: All 70% IG1 5% IG2 100% | - | Appears beneficial |
| Pellegatta 2013(38) | ATL/DC vaccine | RT + CT + 3-7 intradermal vaccinations of variable timing | 4.4 months | 8 months | - | Patients with small tumours at first vaccination (< 20 cm^3^) had significantly longer PFS and OS (6 vs. 3 months, *p* = .01; 16.5 vs. 7 months, *p* = .003, log-rank test). Longer median PFS in patients with high KPS (≥ 70) (5 months vs. 2.5 months, *p* = .005) | Appears unbeneficial |
| Phuphanich 2013(39) | Vaccination with autologous DCs pulsed with peptides from tumour/stem cell-associated antigens (ICT-107) | Surgery + RT + intradermal vaccination in axilla every 2 weeks for 3 doses + TMZ 6 synthetic peptide epitopes targeting GB tumour/stem cell-associated antigens MAGE-1, HER-2, AIM-2, TRP-2, gp100 and IL13Ralpha2 | 16.9 months | 38.4 months | PFS24: 44% OS36: 56% | - | Appears beneficial |
| Reardon 2013(40) | Pazopanib (antiangiogenic) + Lapatinib (ErbBi) | Comparison of PTEN/EGFRvIII positive (P) and PTEN/EGFRvIII negative (N) cohorts Prior therapy: surgery + RT + CT +/- biologics +/- immunotherapy | P: 62 days N: 56 days | - | PFS6: P 0% N 15% | - | Appears unbeneficial |
| Vik-Mo 2013(41) | Vaccination with autologous DCs transfected with mRNA from tumour stem cells | IG: surgery + RT + CT + vaccination twice weekly for 1 week, weekly for 3 weeks then biweekly  CG: surgery + RT + CT | IG: 694 days CG: 236 days | IG: 759 days CG: 585 days | - | Median PFS survival 2.9 times longer in vaccinated patients compared to CG (*p* = .0018, log-rank test). No significant difference in OS between IG and CG (*p* = .11, log-rank test) | Associated with survival benefit |
| Adair 2014(42) | Chemoprotective gene therapy | IG: Autologous p140K gene modified CD34+ HSCs, conferring O6BG resistance. Thereafter, O6BG + TMZ given in 28-day cycles. HCG: O6BG + TMZ. Prior therapy: surgery + RT | 9 months | 20 months | - | Gene therapy associated with a significant increase in the mean number of tolerated O6BG/TMZ cycles compared to HCs (4.4 vs 1.7 cycles, *p* < .05) | Appears beneficial |
| Bloch 2014(43) | Autologous HSPPC-96 vaccine | Surgery + median 6 vaccinations Prior therapy: surgery + RT + CT | 19.1 weeks 35 weeks TTP | 42.6 weeks | OS6: 90% OS12: 29%  PFS6: 29% | Patients with lymphocyte counts below the cohort median had shorter overall survival (HR 4.0, *p* = .012) | Appears beneficial |
| Gallego 2014(44) | Erlotinib (EGFRi) | Erlotinib 150mg/day on a continuous daily basis Prior therapy: surgery + CT + RT | 3.9 months | 7 months | PFS6: 20% | - | Appears unbeneficial |
| Hassler 2014(45) | Imatinib (RTKi) | 400mg oral imatinib OD for a median duration of 3 months Prior therapy: no restriction - surgery + RT + CT | 3 months | 6.2 months | - | - | Appears beneficial |
| Ishikawa 2014(46) | Autologous formalin-fixed tumour vaccine | RT + TMZ + vaccine + TMZ maintenance 3 courses of 5 intradermal injections at weekly intervals. | 8.2 months | 22.2 months | OS24: 47% OS36: 38% PFS24: 33% | Significant association between PFS and age, KPS score, RPA class, extent of tumour removal, IDH1 positivity, and DTH-2 response Significant association between OS and KPS score, RPA class (III/IV vs V), and extent of tumour removal | Associated with survival benefit |
| Olin 2014(47) | Vaccination with autologous DCs pulsed with cells from allogeneic brain tumour cell line GBM6-AD | 5 SC injections 2 weekly then monthly thereafter. Average of 2-10 vaccinations. Prior therapy not specified | *19.1 weeks TTP* | *51 weeks* | - | - | Appears unbeneficial |
| Brown 2015(48) | CAR T cells | CAR–engineered, autologous CD8+ CTLs expressing an IL13 (E13Y)-zetakine CAR targeting IL13R-alpha-2  Up to 12 intracranial infusions into tumour bed via catheter/reservoir system Prior therapy: surgery + RT + CT | - | *11 months* | - | - | Appears unbeneficial |
| Hunn 2015(49) | Autologous DC vaccine | Vaccination with DCs pulsed with autologous tumour cells or pools of long peptides covering 5 glioma associated antigens +/- autologous tumour lysate 3 rounds of priming DC vaccination at 2-week intervals followed by up to 6 cycles of TMZ with booster vaccination after each cycle Prior treatment: surgery + RT + TMZ | *7.8 months TTP* | 23 months | PFS6: 22% | - | Appears unbeneficial |
| Lassman 2015(50) | Dasatinib (RTKi) | IG1: 100mg BD IG2: Dose escalation by 50mg daily every 28 days as tolerated Continuous treatment until intolerance or progression | All: 1.7 months IG1: 1.7 months IG2: 1.8 months | All: 7.9 months IG1: 6.5 months IG2: 8.9 months | PFS6: All 6% IG1 5% IG2 7% | - | Appears unbeneficial |
| Mitchell 2015(51) | Vaccination with cytomegalovirus pp65 RNA pulsed autologous DCs | Bilateral intradermal inguinal vaccination IG1: pre-conditioning of vaccination site with tetanus/diphtheria toxoid IG2: pre-conditioning of vaccination site with mature DCs | IG1: not estimable as 3 patients did not progress and were alive at time of survival analysis  IG2: 4.4 months from randomisation, 10.8 months from diagnosis | IG1: not estimable as 3 patients did not progress and were alive at time of survival analysis  IG2: 11.6 months from randomisation 18.5 months from diagnosis | - | Td-preconditioned patients had a significant increase in both PFS and OS compared to DC-treated patients (p =.013, log-rank test) | Associated with survival benefit |
| Schijns 2015(52) | Gliovac (ERC 1671) | 6 cycles of 5 intradermal injections of autologous antigens from resected tumour + allogeneic antigens from glioma tissue resected from other glioblastoma patients + GM-CSF preceded by low-dose cyclophosphamide Prior therapy: surgery + RT + TMZ +/- bevacizumab | - | - | OS6: IG 100% HCG 33% OS10: IG 77% HCG 10% | - | Appears beneficial |
| Schuster 2015(53) | Rindopepimut (EGFRvIII peptide vaccine) | IG: rindopepimut every 2 weeks for 3 doses then monthly for median of 7.4 months with concurrent standard adjuvant TMZ for ≥ 6 cycles Prior therapy: surgery + CT + RT | 9.2 months | 21.8 months | PFS5.5: 66% OS36: 26% | - | Appears beneficial |
| Akasaki 2016(54) | Fusion cells of autologous DCs and autologous glioma cells | Intradermal vaccination in cervical region 2 weeks after first maintenance TMZ and repeated at least 3 times in each 28-day cycle. After the third dose, vaccination repeated every 6-12 months. IG1: recurrent IG2: newly diagnosed Prior therapy: surgery + RT + CT | IG1: 10.3 months IG2: 18.3 months | IG1: 18 months IG2: 30.5 months | - | - | Appears beneficial |
| Batich 2017(55) | Vaccination with cytomegalovirus pp65 mRNA-pulsed DCs admixed with GM-CSF | DI TMZ + ≥ 3 bilateral intradermal groin vaccinations with monthly cycles continued for up to 10 vaccines if patients had not progressed Prior therapy: surgery + RT + CT | IG: 25.3 months  HCG: 8 months | IG: 41.1 months  HCG: 19.2 months | - | Significantly longer PFS (*p* = .0001, log-rank test) and OS (*p* = .0001, log-rank test) in IG than HCs. All patients exceeded expected median survival, with a median gain in survival of 30 months compared with RPA class predicted median survival | Associated with survival benefit |
| Dunn-Pirio 2017(56) | Vaccination with autologous DCs pulsed with either tumour stem cell mRNA or total tumour RNA | IG: surgery +/- bevacizumab + vaccination weekly for 3 doses then monthly until progression | 3.2 months | 11 months | - | - | Appears unbeneficial |
| Inoges 2017(57) | ATL/DC vaccine | Surgery + RT + TMZ + mean of 8 intradermal vaccinations. First vaccination before RT, second 3 weeks after, then monthly, bimonthly, and quarterly until end of available doses. | 12.7 months | 23.4 months | - | - | Appears beneficial |
| Kong 2017(58) | Adoptive cell therapy | IG: TMZ + IV autologous cytokine-induced killer cells 4 times weekly, then 4 times biweekly and then 6 times at 4 weekly intervals) CG: TMZ | IG: 8.1 months CG: 5.4 months | IG: 22.5 months CG: 16.9 months | PFS12: IG 28% CG 23% PFS18: IG 26% CG 21% PFS24: IG 18% CG 13% OS12: IG 78% CG 75% OS18: IG 57% CG 45% OS24: IG 38% CG 39% | PFS significantly longer in IG than CG (*p* = .0401, log-rank test) No significant difference in OS between IG and CG (*p* =.512, log-rank test) No significant difference in ORR between IG and CG (*p* = .0783, chi-square test) Significantly greater DCR in IG than CG (*p* = .0058, chi square test) | Associated with survival benefit |
| Muragaki 2017(59) | Autologous formalin-fixed tumour vaccine | IG: surgery + AFTV + RT + TMZ CG: surgery + placebo + RT + TMZ | IG: 14.2 months CG: 14.2 months | IG: 21.4 months CG: 21.4 months | PFS6: IG 75% CG 75% OS12: IG 86% CG 86% | - | Appears unbeneficial |
| O'Rourke 2017(60) | EGFRvIII-directed CAR T cells | Single dose of peripherally infused CAR T cells | Not evaluable due to confounding factor of subsequent neurosurgical intervention | 251 days | - | - | Appears unbeneficial |
| Sepúlveda-Sánchez 2017(61) | Dacomitinib (EGFRi) | Oral dacomitinib (45 mg/day) until progression or toxicity IG1: EGFR gene amplification without EGFRvIII mutation. IG2: EGFR gene amplification and EGFRvIII mutation. | All: 2.7 months IG1: 2.7 months IG2: 2.6 months | All: 7.4 months IG1: 7.8 months IG2: 6.7 months | PFS6: All 11% IG1 13% IG2 6% | - | Appears unbeneficial |
| van den Bent 2017(62) | Depatuxizumab mafodotin (EGFR mAb-drug conjugate) | Depatux-m monotherapy every 2 weeks until intolerance or disease progression Prior therapy: RT + TMZ | 1.7 months | 9.3 months | PFS6: 29% OS6: 73%  EGFRvIII positive:  PFS6 17% mPFS 1.6 months | - | Appears beneficial |
| Weller 2017(63) | Rindopepimut (EGFRvIII peptide vaccine) | IG: rindopepimut + TMZ CG: control vaccine + TMZ Monthly intradermal vaccinations until progression or intolerance Prior therapy: surgery + RT + TMZ | IG: 8 months CG: 7.4 months | IG: 20.1 months CG: 20.0 months | mOS IG 17.4 months CG 17.4 (ITT population) | No significant difference in OS between IG and CG for patients with MRD (HR 1.01, *p* = .91) or for ITT analysis (HR 0.89, *p* = .22) No significant difference in PFS between IG and CG for patients with MRD (HR 1.01, *p* = .93) or for ITT analysis (HR 0.94, *p* = .51) | Associated with no survival benefit |
| Buchroithner 2018(64) | ATL/DC vaccine (Audencel) | IG: SOC + weekly vaccine into intra-inguinal lymph node during weeks 7-10 then monthly for an average of 7 months CG: SOC | - | IG: 564 days CG: 568 days | PFS12: IG 28% CG 25% | No significant difference in PFS12 between IG and CG (*p* = .9975, log-rank test)  No significant difference in OS between IG and CG (HR = 0.99, *p* = .89, log-rank test) MGMT M associated with significantly increased OS in CG (*p* = .01, log-rank test) but not IG (*p* = .05, log-rank test) | Associated with no survival benefit |
| Hu 2018(65) | PLB-1001 (MET kinase inhibitor) | Surgery + RT + CT + 50-300mg oral PLB-1001 twice daily until progression or intolerance | *82 days* | - | - | - | Appears unbeneficial |
| Ji 2018(66) | Autologous HSPPC-96 vaccine | Surgery + RT + CT + weekly SC vaccination for 6 weeks | 11.0 months | 31.4 months | PFS6: 90% | mOS for patients with high tumour specific immune response (TSIR) after vaccination was > 40.5 months vs 14.6 months for patients with low TSIR after vaccination (HR 0.25, *p* = .034)  mPFS for patients with high TSIR after vaccination was 12.3 months vs 9 months for patients with low TSIR after vaccination (HR 0.32, *p* = .038) | Appears beneficial |
| Liau 2018(67) | ATL/DC vaccine (DCVax-L) | IG: surgery + CT/RT + TMZ + DCVax-L intradermal vaccine CG: surgery + CT/RT + TMZ + placebo intradermal vaccine Following recurrence, all patients were allowed to receive DCVax-L without unblinding | - | 23.1 months | OS24: 46% OS36: 25% | 30% of ITT population (100/331) had extended survival not fully explained by known prognostic factors, with mOS estimate of 40.5 months | Appears beneficial |
| Pellegatta 2018(68) | ATL/DC vaccine | Surgery + RT + TMZ + up to 7 intradermal vaccinations | 10.5 months | 20.1 months | PFS6: 79% PFS12: 41% OS12: 75% OS24: 37% | Methylation of MGMT promoter associated with longer PFS (median 17.2 months vs 10.2 months, *p* = .02, log-rank test) and OS (median 32.8 months vs 17.8 months, *p* = .03, log-rank test). | Appears beneficial |
| Taylor 2018(69) | Palbociclib (CDK4/6i) | IG1: palbociclib for 7 days prior to resection followed by adjuvant palbociclib IG2: palbociclib without resection 28-day cycles of 21 days oral palbociclib 125mg OD + 7 days break continued until progression or intolerance Prior therapy: surgery + RT + CT | 5.1 weeks | 15.4 weeks | - | - | Appears unbeneficial |
| Yao 2018(70) | Autologous glioblastoma stem cell-like antigen pulsed DC vaccine | Intradermal injections into shoulder  IG: DC vaccine CG: saline placebo vaccine Prior therapy: surgery +/- RT +/- CT | IG: 7.7 months CG: 6.9 months | IG: 13.7 months CG: 10.7 months | - | Significantly longer OS (*p* = .05, log-rank test) but not PFS (*p* = .75, log-rank test) in IG compared to CG.  Significantly longer OS (*p* = .02) in IG than CG after adjusting for IDH1 and TERT promoter mutation status, immune co-inhibitory molecule B7-H4 expression and primary vs recurrent GB. Subgroup analysis among IDH1 wild type and TERT promoter mutation (*p* < .01), and low B7-H4 expression (*p* = .02) patients had significantly prolonged OS in the IG | Associated with survival benefit |
| D'Alessandris 2019(71) | Personalised chemotherapy | Prospective administration of targeted therapy on basis of molecular pattern of VEGF, PTEN and EGFRvIII.  IG1: VEGF over-expression treated with bevacizumab IG2: EGFRvIII expression and normal PTEN treated with bevacizumab + erlotinib IG3: Loss of PTEN irrespective of EGFRvIII status treated with bevacizumab + sirolimus | - | - | PFS6: All 56% IG1 50% IG2 64% IG3 50% PFS12: All 21% IG2 29% | - | Appears beneficial |
| Du 2019(72) | Nimotuzumab (EGFRi mAb) | Surgery + IV nimotuzumab once weekly for 6 weeks + RT + TMZ | 11.9 months | 24.5 months | OS12: 83% PFS12: 49% OS24: 51% PFS24: 29% | No significant correlation between treatment outcomes and MGMT status | Appears beneficial |
| Goff 2019(73) | EGFRvIII-directed CAR T cells | Autologous peripheral blood lymphocytes retrovirally transduced with an EGFRvIII-targeting chimeric antigen receptor. Escalating IV infusions.  Prior therapy: surgery + RT + CT | 1.3 months | *6.9 months* | - | - | Appears unbeneficial |
| Hilf 2019(74) | Actively personalised peptide vaccine | Surgery + RT/CT + intradermal vaccinations preceded by intradermal GM-CSF followed by SC poly-ICLC close to vaccine site. Median 12 APVAC1 and 10 APVAC2 vaccines administered APVAC1: 7 non-mutated HLA class I peptides selected and formulated from a pre-manufactured warehouse of GB-associated peptides, 1 HLA class I viral marker peptide and 2 pan-DR binding HLA class II restricted tumour associated peptides. Schedule: 11 within 21 weeks, commencing day 15 of 1st TMZ cycle APVAC2: composed of mutation-containing peptides synthesised de novo for an individual patient following identification of the mutanome and corresponding mutated peptides in the HLA ligandome (or non-mutated HLA class 1 peptides not part of the warehouse). Schedule: 8 within 9 weeks, commencing day 15 of 4th TMZ cycle | 14.2 months | 29.0 months | - | - | Appears beneficial |
| Keskin 2019(75) | Personalised neoantigen targeting vaccine | Vaccinations of up to 20 long peptides divided into pools of 3-5 peptides admixed with poly-ICLC. 5 priming doses over 4 weeks followed by 2 booster doses 8 and 16 weeks later.  Prior therapy: surgery + RT | 7.6 months | 16.8 months | - | - | Appears unbeneficial |
| Lassman 2019(76) | Depatuxizumab mafodotin (EGFR mAb-drug conjugate) | IG: RT + TMZ + depatux-m 2.0 mg/kg during RT, 1.25 mg/kg thereafter, every 14 days CG: RT + TMZ + placebo | IG: 8.0 months CG: 6.3 months | IG: 18.9 months CG: 18.7 months | - | No difference in OS between IG and CG (HR 1.01, *p* = .63, one-sided log-rank test) | Associated with no survival benefit |
| Lassman 2019(77) | Depatuxizumab mafodotin (EGFR mAb-drug conjugate) | Depatux-m (0.5–1.5 mg/kg) via IV infusion over 30–40 minutes on days 1 and 15, and 150–200 mg/m2 TMZ on days 1–5 of a 28-day cycle continued until progression or toxicity prior therapy: surgery + RT + CT | 2.1 months 3.7 months TTP | 7.4 months | PFS6: 25% OS6: 69% | No correlation between EGFRvIII mutation and PFS or OS | Appears unbeneficial |
| Migliorini 2019(78) | IMA950/poly-ICLC multipeptide vaccine | Surgery + RT + CT + weekly intradermal, SC or IM injections of IMA950 multipeptide vaccine adjuvanted with poly-ICLC. Median 9 vaccinations. | 9.5 months FD 9 months FSE | 19 months FD 17 months FSE | PFS6: 81% FD 69% FT PFS9: 63% FD 56% FT | - | Appears beneficial |
| Narita 2019(79) | Personalised peptide vaccine | IG: vaccine containing 4 of 12 warehouse peptides selected using a patient's pre-existing peptide-specific IgG CG: placebo vaccine Weekly SC vaccinations for 12 weeks followed by biweekly vaccinations until progression | No significant difference between IG and CG. Data not presented. | IG: 8.6 months CG: 8.0 months |  | No significant difference in OS between IG and CG (HR = 1.13, *p* = .621, log-rank test) | Associated with no survival benefit |
| Tien 2019(80) | Ribociclib (CDK4/6i) | Ribociclib 900mg OD for 5 days before tumour resection. Patients with positive PK and PD effects were enrolled in the expansion cohort treated in cycles of 600mg OD for 21 days with 7 days off until progression | 2.1 months | 7.8 months | PFS6: 17% | - | Appears unbeneficial |
| van Gool 2019(81) | Individualised multimodal immunotherapy | Newcastle Disease Virus + modulated electrohyperthermia + IO-VAC (DCs loaded with autologous tumour antigens and matured with a cytokine cocktail and Newcastle Disease Virus) + standard therapy IG1: First-line treatment IG2: Treatment at relapse | - | IG1: 20 months IG2: 7 months | OS24: IG1 40% OS18: IG2 16% | - | Appears beneficial |
| Wen 2019(82) | Vaccination with autologous DCs pulsed with peptides from tumour/stem cell-associated antigens (ICT-107) | IG: ICT-107 vaccine + TMZ CG: Unpulsed DC vaccine + TMZ 4 intradermal axillary vaccinations at weekly intervals then at 1, 3, 6 months and 6 months. 6 synthetic peptide epitopes targeting GB tumour/stem cell-associated antigens MAGE-1, HER-2, AIM-2, TRP-2, gp100 and IL13Ralpha2 Prior therapy: surgery + RT + CT | IG: 11.2 months CG: 9 months | IG: 17 months CG: 15 months | - | No significance difference in OS between IG and CG (HR = 0.87, *p* = .58, log-rank test) PFS significantly longer in IG than CG (HR = 0.57, *p* = .011) | Associated with survival benefit |
| Wen 2019(83) | Buparlisib (pan-PI3Ki) | IG1: patients scheduled for re-operation after progression received buparlisib for 7 to 13 days before surgery to evaluate brain penetration and modulation of the PI3K pathway in resected tumour tissue then buparlisib from post-operative day 14-35 until progression or toxicity IG2: patients not eligible for re-operation received buparlisib until progression or toxicity Buparlisib 100mg OD administered on a continuous 28-day schedule. | IG1: 1.8 months IG2: 1.7 months | IG1: 17.9 months IG2: 9.8 months | PFS6: IG1 27% IG2 8% | - | Appears unbeneficial |
| Chi 2020(84) | Dacomitinib (EGFRi) | IG1: evaluation of tumour penetration in patients with first recurrent EGFR-amplified GB eligible for surgery IG2: phase II trial of 30 patients with EGFR-amplified GB at first recurrence - primary efficacy arm IG3: exploration of dacomitinib in patients with recurrent EGFR-amplified GB after bevacizumab treatment | IG1: 18 weeks IG2: 8.9 weeks IG3: 7.8 weeks | IG1: 39 weeks IG2: 43 weeks IG3: 17 weeks | PFS6: IG2 17% | - | Appears unbeneficial |
| Frenel 2020(85) | Folic acid | Folic acid + TMZ + RT Folic acid dose escalation from 5 to 60mg OD, given 30 mins before TMZ. 60mg given in expansion phase. | 7.9 months | 17.1 months | OS6: 100% OS12: 80% OS24: 28% | - | Appears beneficial |
| Kessler 2020(86) | Molecular profiling-based targeted therapy | Genetic information from MGMT (n = 68), EGFR (n = 7), CDKN2A/B (n = 8), alterations of the PI3K–AKT–mTOR pathway (n = 5), and BRAF (n = 3) most frequently used for therapeutic decision making | - | *416 days in patients with molecular-guided treatment 414 days in patients without molecular-guided treatment* | - | - | Appears unbeneficial |
| Lombardi 2020(87) | Pembrolizumab (anti-PD1 immune checkpoint inhibitor) | Pembrolizumab 200mg once every 3 weeks until disease progression. Median 3 cycles. | *2.6 months* | *7 months* | - | - | Appears unbeneficial |
| Mishinov 2020(88) | ATL/DC vaccine | IG1: surgery + RT + CT + autologous DCs pulsed with autologous tumour lysates + standard treatment IG2: surgery + RT + CT + autologous DCs pulsed with pooled tumour lysates + standard treatment CG: surgery + RT + CT 4-6 SC interscapular vaccinations once every 2 weeks followed by 4-6 injections once per month for 6 months. SC rIL-2 adjuvant. | - | IG1: 16 months FD 12 months FT IG2: 15 months FD 10 months FT CG: 14.5 months FD 12 months FT | - | No significant difference in survival between the 3 groups | Associated with no survival benefit |
| Reardon 2020(89) | Rindopepimut (EGFRvIII peptide vaccine) | IG: rindopepimut + bevacizumab CG: control injection of keyhole limpet hemocyanin + bevacizumab  Mean number of vaccinations IG 9, CG 6 | IG: 3.7 months CG: 3.7 months | - | PFS6: IG 28% CG 16% OS24: IG 20% CG 3% | Statistically significant survival advantage for IG (HR 0.53, *p* = .01, two-sided log-rank test) No significant difference in PFS6 (*p* = .12, one-sided log-rank test) No significant difference in ORR (*p* = .38) | Associated with survival benefit |
| Rudnick 2020(90) | ATL/DC vaccine | Maximal surgical resection with Gliadel (carmustine) wafer placement in the resection cavity followed by intradermal vaccinations 3 times at 2 weekly intervals.  IG1: newly diagnosed  IG2: recurrent disease | All: 3.6 months IG1: 4.8 months IG2: 1.9 months | All: 16.9 months IG1: 27.7 months IG2: 10.9 months | PFS12: All 21% IG1 25% IG2 13% OS12: All 61% IG1 88% IG2 40% | Survival difference between vaccine immune responders and non-vaccine immune responders not statistically significant | Appears unbeneficial |
| Sampson 2020(91) | MDNA55 (engineered IL-4 fused to pseudomonas exotoxin A) | Convection-enhanced intratumoural delivery of MDNA55 as a single treatment via ≤ 4 catheters Median dose 177mg. | - | 11.6 months | OS12: IL4R high 57% IL4R low 33% | Significantly longer OS in MGMT UM IG than MGMT UM CG (*p* = .0268) Significantly longer OS in IG than synthetic control arm (*p* = .0077) No difference in OS between IL4R high and IL4R low groups (*p* = .2175) No difference in OS between IL4R high IG and IL4R high CG (*p* = .0626) | Associated with survival benefit |
| Smith 2020(92) | Adoptive cell therapy | Up to 6 infusions of autologous cytomegalovirus-specific T cells Prior therapy: surgery + RT + CT | 10 months | 21 months | - | Patients treated with ACT before recurrence had significantly improved OS compared with those who progressed before ACT (23 months vs. 14 months, *p* =.018, log-rank test) | Appears beneficial |
| van den Bent 2020(93) | Depatuxizumab mafodotin (EGFR mAb-drug conjugate) | IG1: Depatux-M + TMZ IG2: Depatux-M IG3: lomustine or temozolomide Depatux-M 1.25 mg/kg IV every 2 weeks Median duration of treatment was 16 weeks for IG1 and 9.0 weeks for IG2 Prior therapy: surgery + CT + RT | IG1: 2.7 months IG2: 1.9 months IG3: 1.9 months | IG1: 9.6 months IG2: 7.9 months IG3: 8.2 months | OS12: IG1 40% IG2 27% IG3: 28% OS24: IG1 20% IG2 10% IG3 5% | In the long-term follow-up analysis, OS was significantly longer in Depatux-M + TMZ compared with the control arm (HR 0.66, *p* = .017, log-rank test).  In the primary efficacy analysis at 15 months, the null OS hypothesis was not rejected (HR 0.71, *p* = .06, log-rank test) | Appears beneficial |
| Wang 2020(94) | Autologous DC vaccine transfected with mRNAs encoding personalised tumour-associated antigens | IG: Intradermal injections and IV infusions of TAA-specific DCs at 1:5 ratio + immune adjuvants (low dose cyclophosphamide +poly I:C +imiquimod + anti-PD-1 antibody).  Concurrent treatment: surgery +/- RT +/- CT HCG of 28 patients received SOC treatment by the same physician during the preceding 3 years | - | IG: 19 months CG: 11 months | - | - | Appears beneficial |
| Bonneville-Levard 2021(95) | Personalised molecularly based therapy | Genomic profiling guided personalised CT using NGS on predefined gene panel 3 patients received everolimus, 1 patient received erlotinib, 1 patient received ruxolitinib, 1 patient received sorafenib Prior therapy: surgery + RT + CT | - | *3 months* | - | - | Appears unbeneficial |
| Cardona 2021(96) | Osimertinib (EGFRi) + bevacizumab (VEGFi) | Second line therapy with osimertinib + bevacizumab following recurrence. Fixed dose of osimertinib (80 mg/day) and bevacizumab (15 mg/kg, every 3 weeks). Median 7 bevacizumab cycles. Prior therapy: surgery + RT + TMZ | 5.1 months | 9 months | PFS6: 47% | - | Appears beneficial |
| Padovan 2021(97) | Depatuxizumab mafodotin (EGFR mAb-drug conjugate) | Depatux-M 1.25 mg/kg IV infusion every 2 weeks combined with temozolomide. Median of 4 infusions. Prior therapy: surgery + RT + CT | 2.1 months | 8.0 months | PFS6: 38% OS12: 37% | - | Appears beneficial |
| Werlenius 2021(98) | Adoptive cell therapy | IG: RT + TMZ + IV ALECSAT (Autologous Lymphoid Effector Cells Specific Against Tumour) every 4 weeks for 3 doses CG: RT + TMZ | IG: 7.8 months CG: 7.9 months | IG: 19.2 months CG: 18.3 months | OS12: IG 66% CG 60%  OS24: IG 30% CG 35% | No significant difference in OS (HR 1.16, *p* = .67, log-rank test) or PFS (HR 1.28, *p* = .42, log-rank test) between IG and CG. | Associated with no survival benefit |
| Ciesielski 2022(99) | SurVaxM (survivin peptide vaccine) | MZ CT/RT + 4 priming vaccinations of SurVaxM-Montanide + sargramostim every 2 weeks. Maintenance vaccinations every 12 weeks. Adjuvant TMZ for at least 6 cycles. | 11.4 months | 25.9 months | PFS12: 48%  PFS24:27% PFS36: 23% OS12: 87% OS24: 51% OS36: 41% | Positive correlation between PFS and OS (r = 0.79; 95% CI (0.66,0.87)) | Appears beneficial |
| Hu 2022(100) | Vaccination with autologous DCs pulsed with allogeneic lysate from a glioblastoma stem-like cell line | IG1: newly diagnosed IG2: recurrent Induction phase with vaccine weekly for 4 weeks followed by a maintenance phase with vaccine every 8 weeks until depletion of supply or progression. Newly diagnosed also received RT + TMZ. | IG1: 8.75 months IG2: 3.22 months | IG1: 20.3 months IG2: 12.0 months | PFS6: IG1 73% IG2 24% OS6: IG1 100% IG2 92% | No significant association between vaccine response and PFS or OS. No significant relationships between PFS or OS and MGMT promoter methylation status, age or ethnicity. | Appears beneficial |
| Kasenda 2022(101) | Anti-EGFR immunoliposomes loaded with doxorubicin (anti-EGFR ILs-dox) | Anti-EGFR ILs-dox administered IV at a dose of 50 mg/m2 for a maximum of four cycles. Each treatment cycle was 28 days. Prior therapy: surgery + RT + CT | 1.5 months | 8 months | - | - | Appears unbeneficial |
| van Gool 2022(102) | Individualised multimodal immunotherapy | IG1: surgery + RT/CT + subsequent IMI IG2: surgery + RT/CT + maintenance TMZ + IMI during and after TMZ Median 2 IO-Vac vaccines both groups IMI = (1) Newcastle Disease Virus injections + local modulated electrohyperthermia + (2) DC vaccination (IO-Vac) + (3) personalised modulatory immunotherapy + (4) complementary medicines | - | IG1: 11 months IG2: 22 months | OS24: IG1 0% IG2 36% | Significantly longer survival in IG2 than IG1 (*p* = .0001, log-rank test) | Associated with survival benefit |

**Appendix A: Search Strategies**

**MEDLINE**

1. exp Glioblastoma/ or glioblastoma*.mp.
2. gbm.mp.
3. high grade glioma.mp.
4. hgg.mp.
5. (glioma adj2 grade IV).mp.
6. (astrocytoma adj grade IV).mp.
7. 1 or 2 or 3 or 4 or 5 or 6
8. personal*.mp.
9. stratif*.mp.
10. individual*.mp.
11. precision.mp. or exp Precision Medicine/
12. targeted.mp. or exp Molecular Targeted Therapy/
13. DNA repair.mp. or exp DNA Repair/ or exp DNA Repair Enzymes/
14. autologous tumo?r cell.mp.
15. dendritic cell.mp. or Dendritic Cells/
16. 8 or 9 or 10 or 11 or 12 or 13 or 14 or 15
17. medic*.mp.
18. therap*.mp. or exp Drug Therapy/
19. exp Randomized Controlled Trial/ or exp Clinical Trial/ or trial*.mp.
20. drug*.mp.
21. treat*.mp.
22. genom*.mp.
23. gene*.mp. or exp Genetic Therapy/
24. vaccin*.mp. or exp Cancer Vaccines/
25. chemo*.mp.
26. exp Immunotherapy/ or immunotherap*.mp.
27. 17 or 18 or 19 or 20 or 21 or 22 or 23 or 24 or 25 or 26
28. 16 and 27
29. surviv*.mp. or exp Treatment Outcome/ or Survival Analysis/ or Survival Rate/
30. disease free.mp.
31. progress* free.mp.
32. death.mp. or Death/
33. Mortality/ or mortalit*.mp.
34. Life Expectancy/ or life expectancy.mp.
35. exp Remission Induction/ or remission.mp.
36. recur*.mp.
37. regress*.mp.
38. 29 or 30 or 31 or 32 or 33 or 34 or 35 or 36 or 37
39. 7 and 28 and 38
40. Randomized Controlled Trials as Topic/
41. Randomized Controlled Trial/
42. Random Allocation/
43. Double-Blind Method/
44. Single-Blind Method/
45. Clinical Trial/
46. clinical trial, phase i.pt.
47. clinical trial, phase ii.pt.
48. clinical trial, phase iii.pt.
49. clinical trial, phase iv.pt.
50. controlled clinical trial.pt.
51. randomized controlled trial.pt.
52. multicenter study.pt.
53. clinical trial.pt.
54. exp Clinical Trial/
55. (clinical adj trial$).tw.
56. ((singl$ or doubl$ or treb$ or tripl$) adj (blind$3 or mask$3)).tw.
57. Placebos/
58. placebo$.tw.
59. randomly allocated.tw.
60. (allocated adj2 random$).tw.
61. 40 or 41 or 42 or 43 or 44 or 45 or 46 or 47 or 48 or 49 or 50 or 51 or 52 or 53 or 54
62. 55 or 56 or 57 or 58 or 59 or 60
63. 61 or 62
64. case report.tw.
65. Letter/
66. Historical Article/
67. 64 or 65 or 66
68. 63 not 67
69. Epidemiologic Studies/
70. exp Case-Control Studies/
71. exp Cohort Studies/
72. Case control.tw.
73. (cohort adj (study or studies)).tw.
74. Cohort analy$.tw.
75. (Follow up adj (study or studies)).tw.
76. (observational adj (study or studies)).tw.
77. Longitudinal.tw.
78. Retrospective.tw.
79. Cross sectional.tw.
80. Cross-Sectional Studies/
81. 69 or 70 or 71 or 72 or 73 or 74 or 75 or 76 or 77 or 78 or 79 or 80
82. 68 or 81
83. 39 and 82

**Embase**

1. glioblastoma.mp. or exp glioblastoma/
2. gbm.mp.
3. (high grade adj2 glioma).mp.
4. hgg.mp.
5. (grade IV adj2 glioma).mp.
6. (grade IV adj2 astrocytoma).mp.
7. 1 or 2 or 3 or 4 or 5 or 6
8. exp personalized medicine/ or personal*.mp.
9. stratif*.mp.
10. individual*.mp. or exp individualization/
11. precision.mp.
12. exp molecularly targeted therapy/ or targeted.mp.
13. exp DNA repair/ or (DNA adj2 repair).mp
14. exp tumor cell vaccine/ or exp cancer vaccine/ or exp cancer immunotherapy/ or autologous tumo?r cell.mp. or exp tumor vaccine/ or exp dendritic cell vaccine/
15. exp DNA vaccine/ or dna vaccin*.mp.
16. 8 or 9 or 10 or 11 or 12 or 13 or 14 or 15
17. medic*.mp.
18. therap*.mp.
19. exp phase 4 clinical trial/ or exp phase 2 clinical trial/ or exp controlled clinical trial/ or exp clinical trial/ or exp superiority trial/ or exp pragmatic trial/ or exp "phase 1 clinical trial (topic)"/ or exp "controlled clinical trial (topic)"/ or exp phase 3 clinical trial/ or exp "phase 4 clinical trial(topic)"/ or exp "randomized controlled trial (topic)"/ or exp randomized controlled trial/ or exp "phase 2 clinical trial (topic)"/ or exp non-inferiority trial/ or exp "phase 3 clinical trial (topic)"/ or exp phase 1 clinical trial/
20. drug*.mp.
21. treat*.mp.
22. antineoplastic agent.mp. or exp antineoplastic agent/
23. genom*.mp.
24. gene*.mp.
25. vaccination/ or vaccin*.mp. or vaccine/
26. exp chemoradiotherapy/ or chemo*.mp. or exp cancer chemotherapy/
27. exp immunotherapy/ or immunotherap*.mp.
28. 17 or 18 or 19 or 20 or 21 or 22 or 23 or 24 or 25 or 26 or 27
29. 16 and 28
30. exp mean survival time/ or exp survival bias/ or exp distant recurrence free survival/ or exp survival time/ or exp cancer specific survival/ or exp recurrence free survival/ or exp cancer free survival/ or exp short term survival/ or exp metastasis free survival/ or exp progression free survival/ or exp survival rate/ or exp disease free survival/ or surviv*.mp. or exp cancer survival/ or exp median survival time/ or exp survival analysis/ or exp overall survival/ or exp event free survival/ or exp survival prediction/ or exp long term survival/ or exp survival/
31. disease free.mp.
32. progress* free.mp.
33. death/ or death.mp.
34. mortality/ or mortalit*.mp.
35. life expectancy.mp. or exp life expectancy/
36. exp remission/ or remission.mp.
37. recur*.mp. or exp recurrent disease/ or exp cancer recurrence/
38. regress*.mp.
39. 30 or 31 or 32 or 33 or 34 or 35 or 36 or 37 or 38
40. 7 and 29 and 39
41. clinical trial/
42. randomized controlled trial/
43. controlled clinical trial/
44. multicenter study/
45. phase 3 clinical trial/
46. phase 4 clinical trial/
47. exp randomization/
48. single blind procedure/
49. double blind procedure/
50. crossover procedure/
51. placebo/
52. randomi?ed controlled trial$.tw.
53. rct.tw.
54. (random$ adj2 allocat$).tw.
55. single blind$.tw.
56. double blind$.tw.
57. ((treble or triple) adj blind$).tw.
58. placebo$.tw.
59. prospective study/
60. 41 or 42 or 43 or 44 or 45 or 46 or 47 or 48 or 49 or 50 or 51 or 52 or 53 or 54 or 55 or 56 or 57 or 58 or 59
61. case study/
62. case report.tw.
63. abstract report/
64. letter/
65. Conference proceeding.pt.
66. Conference abstract.pt.
67. Editorial.pt.
68. Letter.pt.
69. Note.pt.
70. 61 or 62 or 63 or 64 or 65 or 66 or 67 or 68 or 69
71. 60 not 70
72. clinical study/
73. case control study/
74. family study/
75. longitudinal study/
76. retrospective study/
77. prospective study/
78. "randomized controlled trial (topic)"/
79. 77 not 78
80. cohort analysis/
81. (Cohort adj (study or studies)).mp.
82. (Case control adj (study or studies)).tw.
83. (follow up adj (study or studies)).tw.
84. (observational adj (study or studies)).tw.
85. (epidemiologic$ adj (study or studies)).tw.
86. (cross sectional adj (study or studies)).tw.
87. 72 or 73 or 74 or 75 or 76 or 79 or 80 or 81 or 82 or 83 or 84 or 85 or 86
88. 71 or 87
89. 40 and 88

**Web of Science**

1. TS=(*glioblastoma* OR *gbm* OR "high grade" NEAR/5 glioma OR *hgg* OR "grade IV" NEAR/5 glioma OR "high grade" NEAR/5 astrocytoma OR "grade IV" NEAR/5 astrocytoma)
2. TS=(personal* OR stratif* OR individual* OR precision OR targeted OR autologous tumo?r cell OR DNA NEAR/5 repair OR dendritic cell)
3. TS=(medic* OR therap* OR trial* OR drug* OR treat* OR antineoplastic agent* OR genom* OR gene* OR vaccin* OR chemo* OR immunotherap*)
4. TS=(surviv* OR disease free OR progress* free OR death OR mortalit*OR life expectancy OR remission OR recur* OR regress*)
5. TS=(randomised OR randomized OR randomisation OR randomization OR placebo* OR (random* AND (allocat* OR assign*)) OR (blind* AND (single OR double OR treble OR triple)))
6. TS=(epidemiologic* OR case control OR cohort OR follow up OR observational OR longitudinal OR retrospective OR prospective OR cross sectional)
7. #2 AND #3
8. #1 AND #7 AND #4
9. #5 OR #6
10. #8 AND #9

**Scopus**

( ( TITLE-ABS-KEY ( ( glioblastoma* ) OR ( gbm ) OR ( "high grade" W/5 glioma ) OR ( hgg ) OR ( "grade iv" W/5 glioma ) OR ( "high grade" W/5 astrocytoma ) OR ( "grade iv" W/5 astrocytoma ) ) ) AND ( ( TITLE-ABS-KEY ( ( personal* ) OR ( stratif* ) OR ( individual* ) OR ( precision ) OR ( targeted ) OR ( "autologous tumo?r cell" ) OR ( dna W/5 repair ) OR ( "dendritic cell" ) ) ) AND ( TITLE-ABS-KEY ( ( medic* ) OR ( therap* ) OR ( trial* ) OR ( drug* ) OR ( treat* ) OR ( "antineoplastic agent*" ) OR ( genom* ) OR ( gene* ) OR ( vaccin* ) OR ( chemo* ) OR ( immunotherap* ) ) ) ) AND ( TITLE-ABS-KEY ( ( surviv* ) OR ( "disease free" ) OR ( "progress* free" ) OR ( death ) OR ( mortalit* ) OR ( "life expectancy" ) OR ( remission ) OR ( recur* ) OR ( regress* ) ) ) ) AND ( ( TITLE-ABS-KEY ( {Clinical-trial} OR {controlled-trial} OR randomi* OR randomly OR ( random W/4 ( allocat* OR distribut* OR assign* ) ) OR {placebo} OR {trial} OR {groups} OR {subgroups} ) OR TITLE ( rct ) ) OR ( TITLE-ABS-KEY ( ( epidemiologic* ) OR ( "case control" ) OR ( cohort ) OR ( "follow up" ) OR ( observational ) OR ( longitudinal ) OR ( retrospective ) OR ( prospective ) OR ( "cross sectional" ) ) ) )

**Appendix B: Full Text Screening Exclusions**

**Table 5.** Studies excluded at full text screening with reasons.

| Study | Exclusion Reason |
| --- | --- |
| Quattrocchi 1999(103) | No survival outcome reported |
| De Vleeschouwer 2008(104) | Mixed cohort including paediatric patients |
| Sampson 2008(105) | Review article |
| Chang 2011(106) | Mixed cohort including oligodendroglioma. Limited glioblastoma specific data. |
| Jie 2012(107) | Mixed cohort including infratentorial tumours |
| Lv 2012(108) | Mixed cohort including 3 patients with grade II/III glioma. No glioblastoma-specific data. |
| Mitsuya 2012(109) | No survival outcome reported |
| Prins 2013(110) | Mixed cohort of grade III and grade IV glioma. Limited glioblastoma-specific data. |
| Muller 2015(111) | Mixed cohort of grade III and grade IV glioma. No glioblastoma-specific data. |
| Reardon 2015(112) | Conference abstract for an included full text paper |
| Fenstermaker 2016(113) | Mixed cohort of grade III and grade IV glioma |
| Phuphanich 2016(114) | Conference abstract for an included full text paper |
| Furuta 2017(115) | Therapy not personalised |
| Ahluwalia 2018(116) | More recent abstract on same trial included |
| Antonios 2018(117) | Mixed cohort of grade III and grade IV glioma |
| Byron 2018(118) | No extractable survival outcome - individual PFS presented in graphical form, OS not reported. |
| Jan 2018(119) | Retrospective analysis of data from another included study |
| Ranjan 2018(120) | No survival outcome reported |
| Boydell 2019(121) | Mixed cohort of grade III and grade IV glioma |
| Caccese 2019(122) | Mixed cohort of grade III and grade IV glioma |
| Garrett 2020(123) | *In vitro* study |
| Hoogstrate 2020(124) | Post hoc analyses on clinical trial data from another included study |
| Ranjan 2020(125) | Mixed cohort of grade III and grade IV glioma |
| van den Bent 2020(126) | No survival outcome reported |
| Batich 2021(127) | Commentary/Opinion |
| Ishikawa 2021(128) | No single specific personalised therapy studied - data on various immunotherapies pooled. |
| Lim-Fat 2021(129) | Therapy not personalised. Compares survival in patients enrolled in clinical trials vs patients not enrolled in clinical trials in a cohort who had undergone somatic tumour sequencing, however only 130 of the 395 enrolled in trials were enrolled based on sequencing results |
| Sim 2021(130) | Therapy not personalised. PARP inhibitor given to MGMT-UM cohort but parallel trial underway on MGMT-M cohort. Has preclinical activity in both M and UM. |
| Baldini 2022(131) | Mixed cohort of grade II, III and IV glioma |
| Van Gool 2022(102) | Duplicate |

**Appendix C: Patient and Study Characteristics Extracted**

- name of first author
- year of publication
- title
- journal
- full text or abstract format
- design
- endpoints
- personalised therapy type
- comparison group
- inclusion criteria
- number of patients
- country of study
- study sponsor/funder
- mean age
- age range
- biological sex
- ethnicity
- mean Karnofsky Performance Status
- newly diagnosed or recurrent disease
- percentage with complete surgical resection
- *MGMT* promoter methylation status
- median time from diagnosis to therapy
- objective response rate
- median PFS
- median OS
- additional survival measures
- relevant statistical analyses performed

**Appendix D: Risk of Bias Assessments using the Joanna Briggs Institute Checklists**

**Key:** Yes (Y), No (N), Unclear (U), Not applicable (N/A), Include (I)

**Randomised trials**

|  | Was true randomization used for assignment of participants to treatment groups? | Was allocation to treatment groups concealed? | Were treatment groups similar at the baseline? | Were participants blind to treatment assignment? | Were those delivering treatment blind to treatment assignment? | Were outcomes assessors blind to treatment assignment? | Were treatment groups treated identically other than the intervention of interest? | Was follow up complete and if not, were differences between groups in terms of their follow up adequately described and analysed? | Were participants analysed in the groups to which they were randomized? | Were outcomes measured in the same way for treatment groups? | Were outcomes measured in a reliable way? | Was appropriate statistical analysis used? | Was the trial design appropriate, and any deviations from the standard RCT design (individual randomization, parallel groups) accounted for in the conduct and analysis of the trial? | Overall appraisal | Comments |
| --- | --- | --- | --- | --- | --- | --- | --- | --- | --- | --- | --- | --- | --- | --- | --- |
| Bloom 1973(1) | U | U | U | U | U | U | U | Y | U | Y | Y | Y | Y | I |  |
| Cho 2012(34) | Y | U | Y | N | N | N | Y | Y | U | Y | Y | Y | Y | I |  |
| Mitchell 2015(51) | U | U | Y | U | U | U | Y | Y | U | Y | Y | Y | Y | I |  |
| Kong 2017(58) | Y | Y | Y | N | N | N | Y | Y | Y | Y | Y | Y | Y | I |  |
| Muragaki 2017(59) | U | U | Y | Y | Y | Y | Y | Y | U | Y | Y | Y | Y | I | Abstract |
| Weller 2017(63) | Y | Y | Y | Y | Y | Y | Y | Y | Y | Y | Y | Y | Y | I |  |
| Buchroithner 2018(64) | Y | Y | Y | N | N | N | Y | Y | Y | Y | Y | Y | Y | I |  |
| Liau 2018(67) | Y | Y | U | Y | Y | Y | Y | Y | Y | Y | Y | Y | Y | I | Interim analysis on total ITT population |
| Yao 2018(70) | Y | U | Y | Y | Y | Y | Y | Y | U | Y | Y | Y | Y | I |  |
| Lassman 2019(76) | U | U | U | Y | Y | Y | Y | Y | U | Y | Y | Y | Y | I | Abstract |
| Narita 2019(79) | Y | U | Y | Y | Y | Y | Y | Y | U | Y | Y | Y | Y | I |  |
| Wen 2019(82) | U | U | Y | Y | Y | Y | Y | U | Y | Y | Y | Y | Y | I |  |
| Mishinov 2020(88) | Y | U | Y | U | U | U | Y | Y | U | Y | Y | Y | Y | I |  |
| Reardon 2020(89) | Y | Y | Y | Y | Y | Y | Y | Y | Y | Y | Y | Y | Y | I |  |
| Van Den Bent 2020(93) | Y | U | Y | N | N | N | Y | Y | Y | Y | Y | Y | Y | I |  |
| Werlenius 2021(98) | U | U | Y | N | N | N | Y | Y | U | Y | Y | Y | Y | I |  |

**Non-randomised trials**

|  | Is it clear in the study what is the cause and what is the effect (i.e. there is no confusion about which variable comes first)? | Were the participants included in any comparisons similar? | Were the participants included in any comparisons receiving similar treatment/care, other than the exposure or intervention of interest? | Was there a control group? | Were there multiple measurements of the outcome both pre and post the intervention/exposure? | Was follow up complete and if not, were differences between groups in terms of their follow up adequately described and analysed? | Were the outcomes of participants included in any comparisons measured in the same way | Were outcomes measured in a reliable way? | Was appropriate statistical analysis used? | Overall appraisal | Comments |
| --- | --- | --- | --- | --- | --- | --- | --- | --- | --- | --- | --- |
| Merchant 1988(2) | Y | N | Y | N | N/A | Y | Y | Y | Y | I |  |
| Lillehei 1991(3) | Y | N/A | N/A | N | N/A | Y | N/A | Y | Y | I |  |
| Hayes 1995(4) | Y | Y | Y | Y | N/A | Y | Y | Y | Y | I | Historical control group |
| Plautz 1998(5) | Y | N/A | N/A | N | N/A | Y | N/A | Y | Y | I |  |
| Schneider 2001(6) | Y | Y | Y | Y | N/A | Y | Y | Y | Y | I |  |
| Yu 2001(7) | Y | Y | Y | Y | N/A | Y | Y | Y | Y | I |  |
| Iwadate 2003(8) | Y | N/A | N/A | N | N/A | Y | N/A | Y | Y | I |  |
| Yamanaka 2003(9) | Y | N/A | N/A | N | N/A | Y | N/A | Y | Y | I |  |
| Steiner 2004(10) | Y | Y | Y | Y | N/A | Y | Y | Y | Y | I |  |
| Wheeler 2004(11) | Y | Y | Y | Y | N/A | Y | Y | Y | Y | I |  |
| Yu 2004(12) | Y | Y | Y | Y | N/A | Y | Y | Y | Y | I |  |
| Liau 2005(13) | Y | Y | Y | Y | N/A | Y | Y | Y | Y | I |  |
| Yajima 2005(14) | Y | N/A | N/A | N | N/A | Y | N/A | Y | Y | I |  |
| Yamanaka 2005(15) | Y | Y | Y | Y | N/A | Y | Y | Y | Y | I |  |
| Fakhrai 2006(16) | Y | N/A | N/A | N | N/A | Y | N/A | Y | Y | I |  |
| Ishikawa 2007(17) | Y | N/A | N/A | N | N/A | Y | N/A | Y | Y | I |  |
| Okada 2007(18) | Y | N/A | N/A | N | N/A | Y | N/A | Y | Y | I |  |
| Izumoto 2008(19) | Y | N/A | N/A | N | N/A | Y | N/A | Y | Y | I |  |
| Wheeler 2008(20) | Y | N/A | N/A | N | N/A | Y | N/A | Y | Y | I |  |
| Dillman 2009(21) | Y | N/A | N/A | N | N/A | Y | N/A | Y | Y | I |  |
| Neyns 2009(22) | Y | Y | Y | Y | N/A | Y | Y | Y | Y | I |  |
| Sampson 2009(23) | Y | N/A | N/A | N | N/A | Y | N/A | Y | Y | I |  |
| Ardon 2010(24) | Y | N/A | N/A | N | N/A | Y | N/A | Y | Y | I |  |
| Clavreul 2010(25) | Y | N/A | N/A | N | N/A | Y | N/A | Y | Y | I |  |
| Iwadate 2010(26) | Y | Y | Y | N | N/A | Y | Y | Y | Y | I |  |
| Sampson 2010(27) | Y | Y | Y | Y | N/A | Y | Y | Y | Y | I |  |
| Fadul 2011(28) | Y | N/A | N/A | N | N/A | Y | N/A | Y | Y | I |  |
| Muragaki 2011(29) | Y | U | Y | N | N/A | Y | Y | Y | Y | I |  |
| Prins 2011(30) | Y | Y | Y | Y | N/A | Y | Y | Y | Y | I |  |
| Sampson 2011(31) | Y | Y | Y | Y | N/A | Y | Y | Y | Y | I | Historical control group |
| Adair 2012(32) | Y | N/A | N/A | N | N/A | Y | N/A | Y | Y | I |  |
| Ardon 2012(33) | Y | Y | Y | N | N/A | Y | Y | Y | Y | I |  |
| Valle 2012(35) | Y | U | U | Y | N/A | Y | Y | Y | Y | I | Historical control group |
| Crane 2013(36) | Y | Y | Y | N | N/A | Y | Y | Y | Y | I |  |
| D'Alessandris 2013(37) | Y | Y | Y | N | N/A | Y | Y | Y | Y | I |  |
| Pellegatta 2013(38) | Y | Y | Y | N | N/A | Y | Y | Y | Y | I |  |
| Phuphanich 2013(39) | Y | Y | Y | N | N/A | Y | Y | Y | Y | I |  |
| Reardon 2013(40) | Y | Y | Y | N | N/A | Y | Y | Y | Y | I |  |
| Vik-Mo 2013(41) | Y | Y | Y | Y | N/A | Y | Y | Y | Y | I | Historical control group |
| Adair 2014(42) | Y | Y | Y | Y | N/A | Y | Y | Y | Y | I | Historical control group |
| Bloch 2014(43) | Y | Y | Y | N | N/A | Y | Y | Y | Y | I |  |
| Gallego 2014(44) | Y | N/A | N/A | N | N/A | Y | N/A | Y | Y | I | Discrepancy between text and table in whether 13 or 14 patients included |
| Hassler 2014(45) | Y | N/A | N/A | N | N/A | Y | N/A | Y | Y | I |  |
| Ishikawa 2014(46) | Y | Y | Y | Y | N/A | Y | Y | Y | Y | I | Historical control group |
| Olin 2014(47) | Y | N/A | N/A | N | N/A | Y | N/A | Y | Y | I | Not powered to assess efficacy |
| Brown 2015(48) | Y | N/A | N/A | N | N/A | Y | N/A | Y | Y | I |  |
| Hunn 2015(49) | Y | Y | Y | N | N/A | Y | Y | Y | Y | I |  |
| Lassman 2015(50) | Y | Y | Y | N | N/A | Y | Y | Y | Y | I |  |
| Schijns 2015(52) | Y | U | U | Y | N/A | Y | Y | Y | Y | I | Historical control group |
| Schuster 2015(53) | Y | Y | Y | Y | N/A | Y | Y | Y | Y | I | Historical control group |
| Akasaki 2016(54) | Y | Y | Y | N | N/A | Y | Y | Y | Y | I | Slightly different OS and PFS figures in abstract and main text table |
| Batich 2017(55) | Y | Y | Y | Y | N/A | Y | Y | Y | Y | I | Historical control group |
| Dunn-Pirio 2017(56) | Y | N/A | N/A | N | N/A | Y | N/A | Y | Y | I |  |
| Inoges 2017(57) | Y | Y | Y | N | N/A | Y | Y | Y | Y | I |  |
| O'Rourke 2017(60) | Y | N/A | N/A | N | N/A | Y | N/A | Y | Y | I |  |
| Sepúlveda-Sánchez 2017(61) | Y | Y | Y | N | N/A | Y | Y | Y | Y | I |  |
| van den Bent 2017(62) | Y | N/A | N/A | N | N/A | Y | N/A | Y | Y | I |  |
| Hu 2018(65) | Y | N/A | N/A | N | N/A | Y | N/A | Y | Y | I |  |
| Ji 2018(66) | Y | U | U | Y | N/A | Y | Y | Y | Y | I | Historical control group |
| Pellegatta 2018(68) | Y | N/A | N/A | N | N/A | Y | N/A | Y | Y | I |  |
| Taylor 2018(69) | Y | N/A | N/A | N | N/A | Y | N/A | Y | Y | I |  |
| D'Alessandris 2019(71) | Y | U | U | N | N/A | Y | Y | Y | Y | I |  |
| Du 2019(72) | Y | Y | Y | N | N/A | Y | Y | Y | Y | I |  |
| Goff 2019(73) | Y | N/A | N/A | N | N/A | Y | N/A | Y | Y | I |  |
| Hilf 2019(74) | Y | N/A | N/A | N | N/A | Y | N/A | Y | Y | I |  |
| Keskin 2019(75) | Y | Y | Y | N | N/A | Y | Y | Y | Y | I |  |
| Lassman 2019(77) | Y | N/A | N/A | N | N/A | Y | N/A | Y | Y | I |  |
| Migliorini 2019(78) | Y | N/A | N/A | N | N/A | Y | N/A | Y | Y | I |  |
| Tien 2019(80) | Y | Y | Y | Y | N/A | Y | Y | Y | Y | I |  |
| Van Gool 2019(81) | Y | Y | Y | N | N/A | Y | Y | Y | Y | I |  |
| Wen 2019(83) | Y | Y | Y | N | N/A | Y | Y | Y | Y | I |  |
| Chi 2020(84) | Y | Y | Y | N | N/A | Y | Y | Y | Y | I |  |
| Frenel 2020(85) | Y | N/A | N/A | N | N/A | Y | N/A | Y | Y | I |  |
| Kessler 2020(86) | Y | N/A | N/A | N | N/A | Y | N/A | Y | Y | I | 30% of patients censored |
| Lombardi 2020(87) | Y | N/A | N/A | N | N/A | Y | N/A | Y | Y | I |  |
| Rudnick 2020(90) | Y | Y | Y | N | N/A | Y | Y | Y | Y | I |  |
| Sampson 2020(91) | Y | Y | Y | Y | N/A | Y | Y | Y | Y | I |  |
| Smith 2020(92) | Y | Y | Y | N | N/A | Y | Y | Y | Y | I |  |
| Wang 2020(94) | Y | N | Y | Y | N/A | Y | Y | Y | Y | I | CG median age 12 years older and contained 1 grade III patient |
| Bonneville-Levard 2021(95) | Y | N/A | N/A | N | N/A | Y | N/A | Y | Y | I |  |
| Cardona 2021(96) | Y | Y | Y | N | N/A | Y | Y | Y | Y | I |  |
| Padovan 2021(97) | Y | U | Y | N | N/A | Y | Y | Y | Y | I | Described as an observational study but appears interventional |
| Ciesielski 2022(99) | Y | U | Y | N | N/A | Y | Y | Y | Y | I |  |
| Hu 2022(100) | Y | N | Y | N | N/A | Y | Y | Y | Y | I | Some differences between groups including ethnicity and MGMT promoter methylation status. |
| Kasenda 2022(101) | Y | N/A | N/A | N | N/A | Y | N/A | Y | Y | I | Higher baseline KPS in group 2 |
| Van Gool 2022(102) | Y | Y | Y | N | N/A | Y | Y | Y | Y | I |  |

**Appendix E: Study Funders**

| Study | Funder |
| --- | --- |
| Bloom 1973(1) | - |
| Merchant 1998(2) | NIH and the Jeffress Memorial Trust |
| Lillehei 1991(3) | American Cancer Society, National Institute for Health, Hoffman-LaRoche Inc., William V. Gervasini Brain Tumour Fund, The Colorado Trust, The Cancer League of Colorado, Pauline Morrison Charitable Trust, St. Joseph Hospital Foundation, the Academic Enrichment and Biomedical Research Support Groups at the University of Colorado, the Colorado Oncology Foundation. |
| Hayes 1995(4) | National Cancer Institute and Department of Neurosurgery, New York University Medical Centre |
| Plautz 1998(5) | National Cancer Institute and Immunex Corporation |
| Schneider 2001(6) | - |
| Yu 2001(7) | NIH |
| Iwadate 2003(8) | - |
| Yamanaka 2003(9) | - |
| Steiner 2004(10) | - |
| Yu 2004(12) | - |
| Wheeler 2004(11) | Joseph Drown Foundation, Maxime Dunitz Neurosurgical Research Fund |
| Liau 2005(13) | NIH, Henry E. Singleton Brain Cancer Research Fund, George Rathmann Family Foundation, Sidney Kimmel Cancer Research Foundation, Jonsson Cancer Center Foundation. |
| Yajima 2005(14) | Ministry of Education, Science, Sports and Culture of Japan, Research Center of Innovative Cancer Therapy of the 21st Century COE Program for Medical Science, Ministry of Health and Welfare of Japan |
| Yamanaka 2005(15) | - |
| Fakhrai 2006(16) | NIH |
| Ishikawa 2007(17) | - |
| Okada 2007(18) | Roche |
| Izumoto 2008(19) | Japanese Ministry of Health, Labour and Welfare and the Japanese Foundation for Multidisciplinary Treatment of Cancer |
| Wheeler 2008(20) | Maxine Dunitz Neurosurgical Research Fund |
| Dillman 2009(21) | The Hoag Hospital Foundation |
| Neyns 2009(22) | Stichting tegen Kanker, Fonds Wetenschappelijk Onderzoek Vlaanderen, Willy Gepts Wetenschappelijk Fonds UZ Brussel. |
| Sampson 2009(23) | NIH, the American Brain Tumour Association, Accelerate Brain Cancer Cure, The Brain Tumour Society |
| Ardon 2010(24) | The Olivia Hendrickx Research Fund, the Herman Memorial Research Fund, the TBM program of the IWT—Flanders, the Belgian Foundation Against Cancer, the Belgian Red Cross, Baxter |
| Clavreul 2010(25) | Medtronic, National Health Department, Ligue Départementale de Lutte contre le Cancer. |
| Iwadate 2010(26) | - |
| Sampson 2010(27) | NIH, the American Brain Tumour Association, Accelerate Brain Cancer Cure, The Brain Tumour Society, Commonwealth Cancer Foundation, the Adam Sliger Foundation, Dr. Marnie Rose Foundation, Anthony Bullock III Foundation, Golfers Against Cancer |
| Fadul 2011(28) | - |
| Muragaki 2011(29) | - |
| Prins 2011(30) | NIH/NCI, the Philip R. and Kenneth A. Jonsson Foundations, the Neidorf Family Foundation, the Ben & Catherine Ivy Foundation, Northwest Biotherapeutics, the Carson Foundation, the UCLA Brain Tumor Translational Resource. |
| Sampson 2011(31) | NIH, NINDS, NCI, the American Brain Tumor Association, Accelerate Brain Cancer Cure, Brain Tumor Society, Goldhirsh Foundation, Commonwealth Cancer Foundation, the Adam Sliger Foundation, Dr Marnie Rose Foundation, and the Anthony Bullock III Foundation |
| Adair 2012(32) | NIH. O6-benzylguanine provided by the Cancer Therapeutics Evaluation Program of the National Cancer Institute. |
| Ardon 2012(33) | The Olivia Hendrickx Research Fund, the Herman Memorial Research Fund, the James E. Kearney Memorial Foundation, CAF Belgium, Baxter, ‘Stichting tegen Kanker’’, IWT (TBM projects), the Stem Cell Institute Leuven, the Emmanuel van der Schueren Fund, the International Union against Cancer, the Klinisch Onderzoeksfonds UZ Leuven, and the Fund for Scientific Research—Flanders (FWO-V). |
| Cho 2012(34) | Taiwan Department of Health Cancer Research Center of Excellence, Taiwan Department of Health Clinical Trial and Research Center of Excellence, National Science Council. |
| Valle 2012(35) | Spanish Health Ministry |
| Crane 2013(36) | Project 5 of the Special Program of Research Excellence Grant, the American Brain Tumor Association, National Brain Tumor Society, Accelerated Brain Cancer Cure |
| D'Alessandris 2013(37) | Roche Spa |
| Pellegatta 2013(38) | - |
| Phuphanich 2013(39) | Musella Foundation For Brain Tumor Research & Information, Inc. and ImmunoCellular Therapeutics Ltd. |
| Reardon 2013(40) | GlaxoSmithKline |
| Vik-Mo 2013(41) | South-Eastern Norway Regional Health Authority, the Norwegian Research Council through the Cancer Stem Cell Innovation Center and the National Stem Cell Center. |
| Adair 2014(42) | NIH |
| Bloch 2014(43) | The National Cancer Institute Special Program of Research Excellence, American Brain Tumor Association, National Brain Tumor Society and the Accelerated Brain Cancer Cure, Inc. |
| Gallego 2014(44) | - |
| Hassler 2014(45) | - |
| Ishikawa 2014(46) | Cell-Medicine, Inc. |
| Olin 2014(47) | PACT, the Randy Shaver Cancer Research and Community Fund, the Hedberg Family/Children’s Cancer Research Fund Chair in brain tumor research and the Cancer Therapeutics Initiative (CETI) Program |
| Brown 2015(48) | NIH and California Institute for Regenerative Medicine |
| Hunn 2015(49) | Cancer Society of New Zealand, New Zealand Health Research Council, the Royal Australasian College of Surgeons and the Surgical Research Trust. |
| Lassman 2015(50) | Radiation Therapy Oncology Group, National Cancer Institute and Bristol-Myers Squibb. |
| Mitchell 2015(51) | NIH, National Institute of Neurological Disorders and Stroke Specialized Program of Research Excellence in Brain Cancer and SRC on Primary and Metastatic Tumors of the CNS, National Brain Tumor Society, the American Brain Tumor Association, Accelerate Brain Cancer Cure Foundation, The Kinetics Foundation, Ben and Catherine Ivy Foundation, Duke University’s Clinical & Translational Science Awards, the National Institutes of Health National Center for Research Resources. |
| Schijns 2015(52) | Epitopoietic Research Corporation |
| Schuster 2015(53) | Celldex Therapeutics, Inc. |
| Akasaki 2016(54) | - |
| Batich 2017(55) | NIH, Small Business Technology Transfer with Annias Immunotherapeutics, Inc., Duke Comprehensive Cancer Core Grant |
| Dunn-Pirio 2017(56) | - |
| Inoges 2017(57) | Spanish Health Ministry |
| Kong 2017(58) | Green Cross Cell Corp |
| Muragaki 2017(59) | - |
| O'Rourke 2017(60) | NCI and Novartis |
| Sepúlveda-Sánchez 2017(61) | Pfizer |
| van den Bent 2017(62) | AbbVie |
| Weller 2017(63) | Celldex Therapeutics, Inc. |
| Buchroithner 2018(64) | Activartis Biotech GmbH |
| Hu 2018(65) | Beijing Pearl Biotechnology LLC, National Key Research and Development Plan, Natural Science Foundation of China/Research Grants Council Hong Kong, China Joint Research Scheme, National Natural Science Foundation of China, Capital Foundation of Medical Developments, Beijing Municipal Administration of Hospitals Clinical Medicine Development of Special Funding Support, Beijing Nova Program, Beijing Administration of Hospitals’ Youth Program, Collaborative Research Fund Hong Kong, Hong Kong Epigenomics Project, HKUST start-up and initiation grants, Ministry of Health & Welfare, Republic of Korea. |
| Ji 2018(66) | National Key Technology Research and Development Program of the Ministry of Science and Technology of China, Beijing Natural Science Foundation, Beijing Talents Fund, and Shenzhen Science and Technology Innovation Committee. |
| Liau 2018(67) | Northwest Biotherapeutics, Inc. |
| Pellegatta 2018(68) | Istituto Neurologico Besta |
| Taylor 2018(69) | Pfizer, National Cancer Institute and the Accelerated Brain Cancer Cure |
| Yao 2018(70) | The Natural Science Foundation of China Grants and the Science and Technology Commission of Shanghai Municipality Grants |
| Chi 2019(84) | Pfizer |
| D'Alessandris 2019(71) | - |
| Du 2019(72) | Biotech Pharmaceutical Co., Ltd. |
| Goff 2019(73) | The Center for Cancer Research of the National Cancer Institute. |
| Hilf 2019(74) | Immatics Biotechnologies GmbH |
| Keskin 2019(75) | Ben and Cather Ivy Foundation, Blavatnik Family Foundation and the Mathers Foundation |
| Lassman 2019(76) | AbbVie |
| Lassman 2019(77) | AbbVie |
| Migliorini 2019(78) | Gateway for Cancer Research, Rising Tide Foundation, Fondation Lionel Perrier, Association Frederic Fellay, Fondation Privée des Hôpitaux Universitaires de Genève, Fond’action, Association Marietta. |
| Narita 2019(79) | BrightPath Biotherapeutics Co, Ltd. |
| Tien 2019(80) | Ben and Catherine Ivy Foundation and NIH Cancer Center Support Grant |
| van Gool 2019(81) | - |
| Wen 2019(82) | Immunocellular Therapeutics. |
| Wen 2019(83) | Ivy Foundation Early Phase Clinical Trials Consortium, DFHCC/MIT Bridge Project and Novartis |
| Frenel 2020(85) | Institut National du Cancer, France |
| Kessler 2020(86) | Deutsche Forschungsgemeinschaft |
| Lombardi 2020(87) | Associazione Italiana Ricerca Sul Cancro and Univesity of Messina |
| Mishinov 2020(88) | Framework of Scientific Research Projects, Russia |
| Reardon 2020(89) | Celldex Therapeutics |
| Rudnick 2020(90) | - |
| Sampson 2020(91) | Medicenna Therapeutics and Cancer Prevention & Research Institute of Texas |
| Smith 2020(92) | Philanthropic funding and the National Health and Medical Research Council (Australia). |
| van den Bent 2020(93) | AbbVie |
| Wang 2020(94) | Beijing Tricision Biotherapeutics Inc and Guangzhou Trinomab Inc. |
| Bonneville-Levard 2021(95) | Institut National du Cancer, Bpifrance Financement abounded by European Community, Agence Nationale de la Recherche (ANR)-LabEx DEvweCAN , European Union, la Fondation ARC, Ligue de l’Ain contre le Cancer, and Centre Leon Bérard. |
| Cardona 2021(96) | Foundation for Clinical and Applied Cancer Research (Bogotá, Colombia) |
| Padovan 2021(97) | Depatux-M was provided by AbbVie |
| Werlenius 2021(98) | Cytovac A/S |
| Ciesielski 2022(99) | MimiVax |
| Hu 2022(100) | Cedars-Sinai Medical Center |
| Kasenda 2022(101) | The Goldschmidt-Jacobson Foundation and the Krebsliga Schweiz |
| van Gool 2022(102) | No funding |

**Appendix F: PRISMA Checklists**

**PRISMA 2020 Checklist**

| **Section and Topic** | **Item #** | **Checklist item** | **Location where item is reported** |
| --- | --- | --- | --- |
| **TITLE** | | |  |
| Title | 1 | Identify the report as a systematic review. | 1 |
| **ABSTRACT** | | |  |
| Abstract | 2 | See the PRISMA 2020 for Abstracts checklist. | 1 |
| **INTRODUCTION** | | |  |
| Rationale | 3 | Describe the rationale for the review in the context of existing knowledge. | 1-2 |
| Objectives | 4 | Provide an explicit statement of the objective(s) or question(s) the review addresses. | 2 |
| **METHODS** | | |  |
| Eligibility criteria | 5 | Specify the inclusion and exclusion criteria for the review and how studies were grouped for the syntheses. | 2 |
| Information sources | 6 | Specify all databases, registers, websites, organisations, reference lists and other sources searched or consulted to identify studies. Specify the date when each source was last searched or consulted. | 3 |
| Search strategy | 7 | Present the full search strategies for all databases, registers and websites, including any filters and limits used. | 3 |
| Selection process | 8 | Specify the methods used to decide whether a study met the inclusion criteria of the review, including how many reviewers screened each record and each report retrieved, whether they worked independently, and if applicable, details of automation tools used in the process. | 3 |
| Data collection process | 9 | Specify the methods used to collect data from reports, including how many reviewers collected data from each report, whether they worked independently, any processes for obtaining or confirming data from study investigators, and if applicable, details of automation tools used in the process. | 4 |
| Data items | 10a | List and define all outcomes for which data were sought. Specify whether all results that were compatible with each outcome domain in each study were sought (e.g. for all measures, time points, analyses), and if not, the methods used to decide which results to collect. | 4 |
|  | 10b | List and define all other variables for which data were sought (e.g. participant and intervention characteristics, funding sources). Describe any assumptions made about any missing or unclear information. | 4 |
| Study risk of bias assessment | 11 | Specify the methods used to assess risk of bias in the included studies, including details of the tool(s) used, how many reviewers assessed each study and whether they worked independently, and if applicable, details of automation tools used in the process. | 4 |
| Effect measures | 12 | Specify for each outcome the effect measure(s) (e.g. risk ratio, mean difference) used in the synthesis or presentation of results. | N/A |
| Synthesis methods | 13a | Describe the processes used to decide which studies were eligible for each synthesis (e.g. tabulating the study intervention characteristics and comparing against the planned groups for each synthesis (item #5)). | 4 |
|  | 13b | Describe any methods required to prepare the data for presentation or synthesis, such as handling of missing summary statistics, or data conversions. | 4 |
|  | 13c | Describe any methods used to tabulate or visually display results of individual studies and syntheses. | 4 |
|  | 13d | Describe any methods used to synthesize results and provide a rationale for the choice(s). If meta-analysis was performed, describe the model(s), method(s) to identify the presence and extent of statistical heterogeneity, and software package(s) used. | 4 |
|  | 13e | Describe any methods used to explore possible causes of heterogeneity among study results (e.g. subgroup analysis, meta-regression). | N/A |
|  | 13f | Describe any sensitivity analyses conducted to assess robustness of the synthesized results. | N/A |
| Reporting bias assessment | 14 | Describe any methods used to assess risk of bias due to missing results in a synthesis (arising from reporting biases). | 4 |
| Certainty assessment | 15 | Describe any methods used to assess certainty (or confidence) in the body of evidence for an outcome. | 4 |
| **RESULTS** | | |  |
| Study selection | 16a | Describe the results of the search and selection process, from the number of records identified in the search to the number of studies included in the review, ideally using a flow diagram. | 4, Figure 2 |
|  | 16b | Cite studies that might appear to meet the inclusion criteria, but which were excluded, and explain why they were excluded. | Appendix B |
| Study characteristics | 17 | Cite each included study and present its characteristics. | Supplementary Table 1 |
| Risk of bias in studies | 18 | Present assessments of risk of bias for each included study. | Appendix D |
| Results of individual studies | 19 | For all outcomes, present, for each study: (a) summary statistics for each group (where appropriate) and (b) an effect estimate and its precision (e.g. confidence/credible interval), ideally using structured tables or plots. | Supplementary Table 3 |
| Results of syntheses | 20a | For each synthesis, briefly summarise the characteristics and risk of bias among contributing studies. | 4-7 |
|  | 20b | Present results of all statistical syntheses conducted. If meta-analysis was done, present for each the summary estimate and its precision (e.g. confidence/credible interval) and measures of statistical heterogeneity. If comparing groups, describe the direction of the effect. | 4-7 |
|  | 20c | Present results of all investigations of possible causes of heterogeneity among study results. | N/A |
|  | 20d | Present results of all sensitivity analyses conducted to assess the robustness of the synthesized results. | N/A |
| Reporting biases | 21 | Present assessments of risk of bias due to missing results (arising from reporting biases) for each synthesis assessed. | 7 |
| Certainty of evidence | 22 | Present assessments of certainty (or confidence) in the body of evidence for each outcome assessed. | 7, Table 1 |
| **DISCUSSION** | | |  |
| Discussion | 23a | Provide a general interpretation of the results in the context of other evidence. | 7-10 |
|  | 23b | Discuss any limitations of the evidence included in the review. | 7-10 |
|  | 23c | Discuss any limitations of the review processes used. | 7-10 |
|  | 23d | Discuss implications of the results for practice, policy, and future research. | 7-10 |
| **OTHER INFORMATION** | | |  |
| Registration and protocol | 24a | Provide registration information for the review, including register name and registration number, or state that the review was not registered. | N/A |
|  | 24b | Indicate where the review protocol can be accessed, or state that a protocol was not prepared. | N/A |
|  | 24c | Describe and explain any amendments to information provided at registration or in the protocol. | N/A |
| Support | 25 | Describe sources of financial or non-financial support for the review, and the role of the funders or sponsors in the review. | 10 |
| Competing interests | 26 | Declare any competing interests of review authors. | N/A |
| Availability of data, code and other materials | 27 | Report which of the following are publicly available and where they can be found: template data collection forms; data extracted from included studies; data used for all analyses; analytic code; any other materials used in the review. | 10 |

**PRISMA 2020 for Abstracts Checklist**

| **Section and Topic** | **Item #** | **Checklist item** | **Reported (Yes/No)** |
| --- | --- | --- | --- |
| **TITLE** | | |  |
| Title | 1 | Identify the report as a systematic review. | Yes |
| **BACKGROUND** | | |  |
| Objectives | 2 | Provide an explicit statement of the main objective(s) or question(s) the review addresses. | Yes |
| **METHODS** | | |  |
| Eligibility criteria | 3 | Specify the inclusion and exclusion criteria for the review. | Yes |
| Information sources | 4 | Specify the information sources (e.g. databases, registers) used to identify studies and the date when each was last searched. | Yes |
| Risk of bias | 5 | Specify the methods used to assess risk of bias in the included studies. | Yes |
| Synthesis of results | 6 | Specify the methods used to present and synthesise results. | Yes |
| **RESULTS** | | |  |
| Included studies | 7 | Give the total number of included studies and participants and summarise relevant characteristics of studies. | Yes |
| Synthesis of results | 8 | Present results for main outcomes, preferably indicating the number of included studies and participants for each. If meta-analysis was done, report the summary estimate and confidence/credible interval. If comparing groups, indicate the direction of the effect (i.e. which group is favoured). | Yes |
| **DISCUSSION** | | |  |
| Limitations of evidence | 9 | Provide a brief summary of the limitations of the evidence included in the review (e.g. study risk of bias, inconsistency and imprecision). | Yes |
| Interpretation | 10 | Provide a general interpretation of the results and important implications. | Yes |
| **OTHER** | | |  |
| Funding | 11 | Specify the primary source of funding for the review. | N/A |
| Registration | 12 | Provide the register name and registration number. | N/A |

**PRISMA S Checklist**

| **Section/topic** | **#** | **Checklist item** | **Location(s) Reported** |
| --- | --- | --- | --- |
| **INFORMATION SOURCES AND METHODS** | | | |
| Database name | 1 | Name each individual database searched, stating the platform for each. | 3 |
| Multi-database searching | 2 | If databases were searched simultaneously on a single platform, state the name of the platform, listing all of the databases searched. | N/A |
| Study registries | 3 | List any study registries searched. | 3 |
| Online resources and browsing | 4 | Describe any online or print source purposefully searched or browsed (e.g., tables of contents, print conference proceedings, web sites), and how this was done. | 3 |
| Citation searching | 5 | Indicate whether cited references or citing references were examined, and describe any methods used for locating cited/citing references (e.g., browsing reference lists, using a citation index, setting up email alerts for references citing included studies). | 3 |
| Contacts | 6 | Indicate whether additional studies or data were sought by contacting authors, experts, manufacturers, or others. | 3 |
| Other methods | 7 | Describe any additional information sources or search methods used. | 3 |
| **SEARCH STRATEGIES** | | | |
| Full search strategies | 8 | Include the search strategies for each database and information source, copied and pasted exactly as run. | Appendix A |
| Limits and restrictions | 9 | Specify that no limits were used, or describe any limits or restrictions applied to a search (e.g., date or time period, language, study design) and provide justification for their use. | 3 |
| Search filters | 10 | Indicate whether published search filters were used (as originally designed or modified), and if so, cite the filter(s) used. | 3 |
| Prior work | 11 | Indicate when search strategies from other literature reviews were adapted or reused for a substantive part or all of the search, citing the previous review(s). | N/A |
| Updates | 12 | Report the methods used to update the search(es) (e.g., rerunning searches, email alerts). | N/A |
| Dates of searches | 13 | For each search strategy, provide the date when the last search occurred. | 3 |
| **PEER REVIEW** | | | |
| Peer review | 14 | Describe any search peer review process. | 10 |
| **MANAGING RECORDS** | | | |
| Total Records | 15 | Document the total number of records identified from each database and other information sources. | Figure 2 |
| Deduplication | 16 | Describe the processes and any software used to deduplicate records from multiple database searches and other information sources. | 3 |

**Appendix G: References used in Supplementary Materials**

1. Bloom HJ, Peckham MJ, Richardson AE, Alexander PA, Payne PM. Glioblastoma multiforme: a controlled trial to assess the value of specific active immunotherapy in patients treated by radical surgery and radiotherapy. Br J Cancer. 1973 Mar;27(3):253–67.

2. Merchant RE, Grant AJ, Merchant LH, Young HF. Adoptive immunotherapy for recurrent glioblastoma multiforme using lymphokine activated killer cells and recombinant interleukin-2. Cancer. 1988 Aug 15;62(4):665–71.

3. Lillehei KO, Mitchell DH, Johnson SD, McCleary EL, Kruse CA. Long-term follow-up of patients with recurrent malignant gliomas treated with adjuvant adoptive immunotherapy. Neurosurgery. 1991 Jan;28(1):16–23.

4. Hayes RL, Koslow M, Hiesiger EM, Hymes KB, Hochster HS, Moore EJ, et al. Improved long term survival after intracavitary interleukin-2 and lymphokine-activated killer cells for adults with recurrent malignant glioma. Cancer. 1995 Sep 1;76(5):840–52.

5. Plautz GE, Barnett GH, Miller DW, Cohen BH, Prayson RA, Krauss JC, et al. Systemic T cell adoptive immunotherapy of malignant gliomas. J Neurosurg. 1998 Jul;89(1):42–51.

6. Schneider T, Gerhards R, Kirches E, Firsching R. Preliminary results of active specific immunization with modified tumor cell vaccine in glioblastoma multiforme. J Neurooncol. 2001 May;53(1):39–46.

7. Yu JS, Wheeler CJ, Zeltzer PM, Ying H, Finger DN, Lee PK, et al. Vaccination of malignant glioma patients with peptide-pulsed dendritic cells elicits systemic cytotoxicity and intracranial T-cell infiltration. Cancer Res. 2001 Feb 1;61(3):842–7.

8. Iwadate Y, Fujimoto S, Namba H, Yamaura A. Promising survival for patients with glioblastoma multiforme treated with individualised chemotherapy based on in vitro drug sensitivity testing. Br J Cancer. 2003 Nov 17;89(10):1896–900.

9. Yamanaka R, Abe T, Yajima N, Tsuchiya N, Homma J, Kobayashi T, et al. Vaccination of recurrent glioma patients with tumour lysate-pulsed dendritic cells elicits immune responses: results of a clinical phase I/II trial. Br J Cancer. 2003 Oct 6;89(7):1172–9.

10. Steiner HH, Bonsanto MM, Beckhove P, Brysch M, Geletneky K, Ahmadi R, et al. Antitumor vaccination of patients with glioblastoma multiforme: a pilot study to assess feasibility, safety, and clinical benefit. J Clin Oncol. 2004 Nov 1;22(21):4272–81.

11. Wheeler CJ, Das A, Liu G, Yu JS, Black KL. Clinical responsiveness of glioblastoma multiforme to chemotherapy after vaccination. Clin Cancer Res. 2004 Aug 15;10(16):5316–26.

12. Yu JS, Liu G, Ying H, Yong WH, Black KL, Wheeler CJ. Vaccination with tumor lysate-pulsed dendritic cells elicits antigen-specific, cytotoxic T-cells in patients with malignant glioma. Cancer Res. 2004 Jul 15;64(14):4973–9.

13. Liau LM, Prins RM, Kiertscher SM, Odesa SK, Kremen TJ, Giovannone AJ, et al. Dendritic Cell Vaccination in Glioblastoma Patients Induces Systemic and Intracranial T-cell Responses Modulated by the Local Central Nervous System Tumor Microenvironment. Clin Cancer Res. 2005 Aug 1;11(15):5515–25.

14. Yajima N, Yamanaka R, Mine T, Tsuchiya N, Homma J, Sano M, et al. Immunologic evaluation of personalized peptide vaccination for patients with advanced malignant glioma. Clin Cancer Res. 2005 Aug 15;11(16):5900–11.

15. Yamanaka R, Homma J, Yajima N, Tsuchiya N, Sano M, Kobayashi T, et al. Clinical evaluation of dendritic cell vaccination for patients with recurrent glioma: results of a clinical phase I/II trial. Clin Cancer Res. 2005 Jun 1;11(11):4160–7.

16. Fakhrai H, Mantil JC, Liu L, Nicholson GL, Murphy-Satter CS, Ruppert J, et al. Phase I clinical trial of a TGF-beta antisense-modified tumor cell vaccine in patients with advanced glioma. Cancer Gene Ther. 2006 Dec;13(12):1052–60.

17. Ishikawa E, Tsuboi K, Yamamoto T, Muroi A, Takano S, Enomoto T, et al. Clinical trial of autologous formalin-fixed tumor vaccine for glioblastoma multiforme patients. Cancer Sci. 2007 Aug;98(8):1226–33.

18. Okada H, Lieberman FS, Walter KA, Lunsford LD, Kondziolka DS, Bejjani GK, et al. Autologous glioma cell vaccine admixed with interleukin-4 gene transfected fibroblasts in the treatment of patients with malignant gliomas. J Transl Med. 2007 Dec 19;5:67.

19. Izumoto S, Tsuboi A, Oka Y, Suzuki T, Hashiba T, Kagawa N, et al. Phase II clinical trial of Wilms tumor 1 peptide vaccination for patients with recurrent glioblastoma multiforme. J Neurosurg. 2008 May;108(5):963–71.

20. Wheeler CJ, Black KL, Liu G, Mazer M, Zhang X xue, Pepkowitz S, et al. Vaccination elicits correlated immune and clinical responses in glioblastoma multiforme patients. Cancer Res. 2008 Jul 15;68(14):5955–64.

21. Dillman RO, Duma CM, Ellis RA, Cornforth AN, Schiltz PM, Sharp SL, et al. Intralesional lymphokine-activated killer cells as adjuvant therapy for primary glioblastoma. J Immunother. 2009 Dec;32(9):914–9.

22. Neyns B, Sadones J, Joosens E, Bouttens F, Verbeke L, Baurain JF, et al. Stratified phase II trial of cetuximab in patients with recurrent high-grade glioma. Ann Oncol. 2009 Sep;20(9):1596–603.

23. Sampson JH, Archer GE, Mitchell DA, Heimberger AB, Herndon JE, Lally-Goss D, et al. An epidermal growth factor receptor variant III-targeted vaccine is safe and immunogenic in patients with glioblastoma multiforme. Mol Cancer Ther. 2009 Oct;8(10):2773–9.

24. Ardon H, Van Gool S, Lopes IS, Maes W, Sciot R, Wilms G, et al. Integration of autologous dendritic cell-based immunotherapy in the primary treatment for patients with newly diagnosed glioblastoma multiforme: a pilot study. J Neurooncol. 2010 Sep;99(2):261–72.

25. Clavreul A, Piard N, Tanguy JY, Gamelin E, Rousselet MC, Leynia P, et al. Autologous tumor cell vaccination plus infusion of GM-CSF by a programmable pump in the treatment of recurrent malignant gliomas. J Clin Neurosci. 2010 Jul;17(7):842–8.

26. Iwadate Y, Matsutani T, Hasegawa Y, Shinozaki N, Oide T, Tanizawa T, et al. Selection of chemotherapy for glioblastoma expressing O(6)-methylguanine-DNA methyltransferase. Exp Ther Med. 2010 Jan;1(1):53–7.

27. Sampson JH, Heimberger AB, Archer GE, Aldape KD, Friedman AH, Friedman HS, et al. Immunologic escape after prolonged progression-free survival with epidermal growth factor receptor variant III peptide vaccination in patients with newly diagnosed glioblastoma. J Clin Oncol. 2010 Nov 1;28(31):4722–9.

28. Fadul CE, Fisher JL, Hampton TH, Lallana EC, Li Z, Gui J, et al. Immune response in patients with newly diagnosed glioblastoma multiforme treated with intranodal autologous tumor lysate-dendritic cell vaccination after radiation chemotherapy. J Immunother. 2011 May;34(4):382–9.

29. Muragaki Y, Maruyama T, Iseki H, Tanaka M, Shinohara C, Takakura K, et al. Phase I/IIa trial of autologous formalin-fixed tumor vaccine concomitant with fractionated radiotherapy for newly diagnosed glioblastoma. Clinical article. J Neurosurg. 2011 Aug;115(2):248–55.

30. Prins RM, Soto H, Konkankit V, Odesa SK, Eskin A, Yong WH, et al. Gene expression profile correlates with T-cell infiltration and relative survival in glioblastoma patients vaccinated with dendritic cell immunotherapy. Clin Cancer Res. 2011 Mar 15;17(6):1603–15.

31. Sampson JH, Aldape KD, Archer GE, Coan A, Desjardins A, Friedman AH, et al. Greater chemotherapy-induced lymphopenia enhances tumor-specific immune responses that eliminate EGFRvIII-expressing tumor cells in patients with glioblastoma. Neuro Oncol. 2011 Mar;13(3):324–33.

32. Adair JE, Beard BC, Trobridge GD, Neff T, Rockhill JK, Silbergeld DL, et al. Extended survival of glioblastoma patients after chemoprotective HSC gene therapy. Sci Transl Med. 2012 May 9;4(133):133ra57.

33. Ardon H, Van Gool SW, Verschuere T, Maes W, Fieuws S, Sciot R, et al. Integration of autologous dendritic cell-based immunotherapy in the standard of care treatment for patients with newly diagnosed glioblastoma: results of the HGG-2006 phase I/II trial. Cancer Immunol Immunother. 2012 Nov;61(11):2033–44.

34. Cho DY, Yang WK, Lee HC, Hsu DM, Lin HL, Lin SZ, et al. Adjuvant immunotherapy with whole-cell lysate dendritic cells vaccine for glioblastoma multiforme: a phase II clinical trial. World Neurosurg. 2012 Jun;77(5–6):736–44.

35. Valle RD, de Cerio ALD, Inoges S, Tejada S, Pastor F, Villanueva H, et al. Dendritic cell vaccination in glioblastoma after fluorescence-guided resection. World J Clin Oncol. 2012 Nov 10;3(11):142–9.

36. Crane CA, Han SJ, Ahn B, Oehlke J, Kivett V, Fedoroff A, et al. Individual patient-specific immunity against high-grade glioma after vaccination with autologous tumor derived peptides bound to the 96 KD chaperone protein. Clin Cancer Res. 2013 Jan 1;19(1):205–14.

37. D’Alessandris QG, Montano N, Cenci T, Martini M, Lauretti L, Bianchi F, et al. Targeted therapy with bevacizumab and erlotinib tailored to the molecular profile of patients with recurrent glioblastoma. Preliminary experience. Acta Neurochir (Wien). 2013 Jan;155(1):33–40.

38. Pellegatta S, Eoli M, Frigerio S, Antozzi C, Bruzzone MG, Cantini G, et al. The natural killer cell response and tumor debulking are associated with prolonged survival in recurrent glioblastoma patients receiving dendritic cells loaded with autologous tumor lysates. Oncoimmunology. 2013 Mar 1;2(3):e23401.

39. Phuphanich S, Wheeler CJ, Rudnick JD, Mazer M, Wang H, Nuño MA, et al. Phase I trial of a multi-epitope-pulsed dendritic cell vaccine for patients with newly diagnosed glioblastoma. Cancer Immunol Immunother. 2013 Jan;62(1):125–35.

40. Reardon DA, Groves MD, Wen PY, Nabors L, Mikkelsen T, Rosenfeld S, et al. A phase I/II trial of pazopanib in combination with lapatinib in adult patients with relapsed malignant glioma. Clin Cancer Res. 2013 Feb 15;19(4):900–8.

41. Vik-Mo EO, Nyakas M, Mikkelsen BV, Moe MC, Due-Tønnesen P, Suso EMI, et al. Therapeutic vaccination against autologous cancer stem cells with mRNA-transfected dendritic cells in patients with glioblastoma. Cancer Immunol Immunother. 2013 Sep;62(9):1499–509.

42. Adair JE, Johnston SK, Mrugala MM, Beard BC, Guyman LA, Baldock AL, et al. Gene therapy enhances chemotherapy tolerance and efficacy in glioblastoma patients. J Clin Invest. 2014 Sep;124(9):4082–92.

43. Bloch O, Crane CA, Fuks Y, Kaur R, Aghi MK, Berger MS, et al. Heat-shock protein peptide complex-96 vaccination for recurrent glioblastoma: a phase II, single-arm trial. Neuro Oncol. 2014 Jan;16(2):274–9.

44. Gallego O, Cuatrecasas M, Benavides M, Segura PP, Berrocal A, Erill N, et al. Efficacy of erlotinib in patients with relapsed gliobastoma multiforme who expressed EGFRVIII and PTEN determined by immunohistochemistry. J Neurooncol. 2014 Jan;116(2):413–9.

45. Hassler MR, Vedadinejad M, Flechl B, Haberler C, Preusser M, Hainfellner JA, et al. Response to imatinib as a function of target kinase expression in recurrent glioblastoma. Springerplus. 2014;3:111.

46. Ishikawa E, Muragaki Y, Yamamoto T, Maruyama T, Tsuboi K, Ikuta S, et al. Phase I/IIa trial of fractionated radiotherapy, temozolomide, and autologous formalin-fixed tumor vaccine for newly diagnosed glioblastoma. J Neurosurg. 2014 Sep;121(3):543–53.

47. Olin MR, Low W, McKenna DH, Haines SJ, Dahlheimer T, Nascene D, et al. Vaccination with dendritic cells loaded with allogeneic brain tumor cells for recurrent malignant brain tumors induces a CD4(+)IL17(+) response. J Immunother Cancer. 2014;2:4.

48. Brown CE, Badie B, Barish ME, Weng L, Ostberg JR, Chang WC, et al. Bioactivity and Safety of IL13Rα2-Redirected Chimeric Antigen Receptor CD8+ T Cells in Patients with Recurrent Glioblastoma. Clin Cancer Res. 2015 Sep 15;21(18):4062–72.

49. Hunn MK, Bauer E, Wood CE, Gasser O, Dzhelali M, Ancelet LR, et al. Dendritic cell vaccination combined with temozolomide retreatment: results of a phase I trial in patients with recurrent glioblastoma multiforme. J Neurooncol. 2015 Jan;121(2):319–29.

50. Lassman AB, Pugh SL, Gilbert MR, Aldape KD, Geinoz S, Beumer JH, et al. Phase 2 trial of dasatinib in target-selected patients with recurrent glioblastoma (RTOG 0627). Neuro Oncol. 2015 Jul;17(7):992–8.

51. Mitchell DA, Batich KA, Gunn MD, Huang MN, Sanchez-Perez L, Nair SK, et al. Tetanus toxoid and CCL3 improve dendritic cell vaccines in mice and glioblastoma patients. Nature. 2015 Mar 19;519(7543):366–9.

52. Schijns VEJC, Pretto C, Devillers L, Pierre D, Hofman FM, Chen TC, et al. First clinical results of a personalized immunotherapeutic vaccine against recurrent, incompletely resected, treatment-resistant glioblastoma multiforme (GBM) tumors, based on combined allo- and auto-immune tumor reactivity. Vaccine. 2015 May 28;33(23):2690–6.

53. Schuster J, Lai RK, Recht LD, Reardon DA, Paleologos NA, Groves MD, et al. A phase II, multicenter trial of rindopepimut (CDX-110) in newly diagnosed glioblastoma: the ACT III study. Neuro Oncol. 2015 Jun;17(6):854–61.

54. Akasaki Y, Kikuchi T, Homma S, Koido S, Ohkusa T, Tasaki T, et al. Phase I/II trial of combination of temozolomide chemotherapy and immunotherapy with fusions of dendritic and glioma cells in patients with glioblastoma. Cancer Immunol Immunother. 2016 Dec;65(12):1499–509.

55. Batich KA, Reap EA, Archer GE, Sanchez-Perez L, Nair SK, Schmittling RJ, et al. Long-term Survival in Glioblastoma with Cytomegalovirus pp65-Targeted Vaccination. Clin Cancer Res. 2017 Apr 15;23(8):1898–909.

56. Dunn-Pirio A, Peters K, DesJardins A, Randazzo D, Friedman H, Healy P, et al. Tumor stem cell RNA-loaded dendritic cell vaccine for recurrent glioblastoma: a phase 1 trial (S41.004). Neurology. 2017 Apr 18;88(16 Supplement):S41.004.

57. Inoges S, Tejada S, de Cerio A, Perez-Larraya J, Espinos J, Idoate M, et al. A phase II trial of autologous dendritic cell vaccination and radiochemotherapy following fluorescence-guided surgery in newly diagnosed glioblastoma patients. Journal of Translational Medicine. 2017 May 12;15.

58. Kong DS, Nam DH, Kang SH, Lee JW, Chang JH, Kim JH, et al. Phase III randomized trial of autologous cytokine-induced killer cell immunotherapy for newly diagnosed glioblastoma in Korea. Oncotarget. 2017 Jan 24;8(4):7003–13.

59. Muragaki Y, Maruyama T, Ishikawa E, Nitta M, Ikuta S, Yamamoto T, et al. OS09.8 Randomized placebo-controlled trial of autologous formalin-fixed tumor vaccine for newly diagnosed glioblastoma. Neuro Oncol. 2017 May;19(Suppl 3):iii20.

60. O’Rourke DM, Nasrallah MP, Desai A, Melenhorst JJ, Mansfield K, Morrissette JJD, et al. A single dose of peripherally infused EGFRvIII-directed CAR T cells mediates antigen loss and induces adaptive resistance in patients with recurrent glioblastoma. Sci Transl Med. 2017 Jul 19;9(399).

61. Sepúlveda-Sánchez JM, Vaz MÁ, Balañá C, Gil-Gil M, Reynés G, Gallego Ó, et al. Phase II trial of dacomitinib, a pan-human EGFR tyrosine kinase inhibitor, in recurrent glioblastoma patients with EGFR amplification. Neuro Oncol. 2017 Oct 19;19(11):1522–31.

62. van den Bent M, Gan HK, Lassman AB, Kumthekar P, Merrell R, Butowski N, et al. Efficacy of depatuxizumab mafodotin (ABT-414) monotherapy in patients with EGFR-amplified, recurrent glioblastoma: results from a multi-center, international study. Cancer Chemother Pharmacol. 2017 Dec;80(6):1209–17.

63. Weller M, Butowski N, Tran DD, Recht LD, Lim M, Hirte H, et al. Rindopepimut with temozolomide for patients with newly diagnosed, EGFRvIII-expressing glioblastoma (ACT IV): a randomised, double-blind, international phase 3 trial. Lancet Oncol. 2017 Oct;18(10):1373–85.

64. Buchroithner J, Erhart F, Pichler J, Widhalm G, Preusser M, Stockhammer G, et al. Audencel Immunotherapy Based on Dendritic Cells Has No Effect on Overall and Progression-Free Survival in Newly Diagnosed Glioblastoma: A Phase II Randomized Trial. Cancers (Basel). 2018 Oct 5;10(10):E372.

65. Hu H, Mu Q, Bao Z, Chen Y, Liu Y, Chen J, et al. Mutational Landscape of Secondary Glioblastoma Guides MET-Targeted Trial in Brain Tumor. Cell. 2018 Nov 29;175(6):1665-1678.e18.

66. Ji N, Zhang Y, Liu Y, Xie J, Wang Y, Hao S, et al. Heat shock protein peptide complex-96 vaccination for newly diagnosed glioblastoma: a phase I, single-arm trial. JCI Insight. 2018 May 17;3(10):99145.

67. Liau LM, Ashkan K, Tran DD, Campian JL, Trusheim JE, Cobbs CS, et al. First results on survival from a large Phase 3 clinical trial of an autologous dendritic cell vaccine in newly diagnosed glioblastoma. Journal of Translational Medicine. 2018 May 29;16(1):142.

68. Pellegatta S, Eoli M, Cuccarini V, Anghileri E, Pollo B, Pessina S, et al. Survival gain in glioblastoma patients treated with dendritic cell immunotherapy is associated with increased NK but not CD8+ T cell activation in the presence of adjuvant temozolomide. Oncoimmunology. 2018;7(4):e1412901.

69. Taylor JW, Parikh M, Phillips JJ, James CD, Molinaro AM, Butowski NA, et al. Phase-2 trial of palbociclib in adult patients with recurrent RB1-positive glioblastoma. J Neurooncol. 2018 Nov;140(2):477–83.

70. Yao Y, Luo F, Tang C, Chen D, Qin Z, Hua W, et al. Molecular subgroups and B7-H4 expression levels predict responses to dendritic cell vaccines in glioblastoma: an exploratory randomized phase II clinical trial. Cancer Immunol Immunother. 2018 Nov;67(11):1777–88.

71. D’Alessandris GQ, Montano N, Martini M, Cenci T, Di Bonaventura R, Giordano M, et al. INNV-41. Prospective selection of recurrent glioblastoma patients for tailored therapies. Results of an institutional experience. Neuro Oncol. 2019 Nov;21(Suppl 6):vi138.

72. Du XJ, Li XM, Cai LB, Sun JC, Wang SY, Wang XC, et al. Efficacy and safety of nimotuzumab in addition to radiotherapy and temozolomide for cerebral glioblastoma: a phase II multicenter clinical trial. J Cancer. 2019;10(14):3214–23.

73. Goff SL, Morgan RA, Yang JC, Sherry RM, Robbins PF, Restifo NP, et al. Pilot Trial of Adoptive Transfer of Chimeric Antigen Receptor-transduced T Cells Targeting EGFRvIII in Patients With Glioblastoma. J Immunother. 2019 May;42(4):126–35.

74. Hilf N, Kuttruff-Coqui S, Frenzel K, Bukur V, Stevanović S, Gouttefangeas C, et al. Actively personalized vaccination trial for newly diagnosed glioblastoma. Nature. 2019 Jan;565(7738):240–5.

75. Keskin DB, Anandappa AJ, Sun J, Tirosh I, Mathewson ND, Li S, et al. Neoantigen vaccine generates intratumoral T cell responses in phase Ib glioblastoma trial. Nature. 2019 Jan;565(7738):234–9.

76. Lassman A, Pugh S, Wang T, Aldape K, Gan H, Preusser M, et al. ACTR-21. A randomized, double-blind, placebo-controlled phase 3 trial of depatuxizumab mafodotin (ABT-414) in epidermal growth factor receptor (EGFR) amplified (AMP) newly diagnosed glioblastoma (nGBM). Neuro Oncol. 2019 Nov;21(Suppl 6):vi17.

77. Lassman AB, van den Bent MJ, Gan HK, Reardon DA, Kumthekar P, Butowski N, et al. Safety and efficacy of depatuxizumab mafodotin + temozolomide in patients with EGFR-amplified, recurrent glioblastoma: results from an international phase I multicenter trial. Neuro Oncol. 2019 Jan 1;21(1):106–14.

78. Migliorini D, Dutoit V, Allard M, Grandjean Hallez N, Marinari E, Widmer V, et al. Phase I/II trial testing safety and immunogenicity of the multipeptide IMA950/poly-ICLC vaccine in newly diagnosed adult malignant astrocytoma patients. Neuro Oncol. 2019 Jul 11;21(7):923–33.

79. Narita Y, Arakawa Y, Yamasaki F, Nishikawa R, Aoki T, Kanamori M, et al. A randomized, double-blind, phase III trial of personalized peptide vaccination for recurrent glioblastoma. Neuro Oncol. 2019 Feb 19;21(3):348–59.

80. Tien AC, Li J, Bao X, Derogatis A, Kim S, Mehta S, et al. A Phase 0 Trial of Ribociclib in Recurrent Glioblastoma Patients Incorporating a Tumor Pharmacodynamic- and Pharmacokinetic-Guided Expansion Cohort. Clin Cancer Res. 2019 Oct 1;25(19):5777–86.

81. Van Gool S, Makalowski J, Stuecker W. ATIM-13.Multimodal immunotherapy with IO-VAC® for patients with GBM: a single institution experience. Neuro-Oncology. 2019 Nov 11;21(Supplement_6):vi4.

82. Wen PY, Reardon DA, Armstrong TS, Phuphanich S, Aiken RD, Landolfi JC, et al. A Randomized Double-Blind Placebo-Controlled Phase II Trial of Dendritic Cell Vaccine ICT-107 in Newly Diagnosed Patients with Glioblastoma. Clin Cancer Res. 2019 Oct 1;25(19):5799–807.

83. Wen PY, Touat M, Alexander BM, Mellinghoff IK, Ramkissoon S, McCluskey CS, et al. Buparlisib in Patients With Recurrent Glioblastoma Harboring Phosphatidylinositol 3-Kinase Pathway Activation: An Open-Label, Multicenter, Multi-Arm, Phase II Trial. J Clin Oncol. 2019 Mar 20;37(9):741–50.

84. Chi AS, Cahill DP, Reardon DA, Wen PY, Mikkelsen T, Peereboom DM, et al. Exploring Predictors of Response to Dacomitinib in EGFR-Amplified Recurrent Glioblastoma. JCO Precis Oncol. 2020;4:PO.19.00295.

85. Frenel JS, Cartron PF, Gourmelon C, Campion L, Aumont M, Augereau P, et al. 370MO FOLAGLI: A phase I study of folinic acid combined with temozolomide and radiotherapy to modulate MGMT gene promoter methylation in newly diagnosed MGMT non-methytated glioblastoma. Annals of Oncology. 2020 Sep 1;31:S400.

86. Kessler T, Berberich A, Casalini B, Drüschler K, Ostermann H, Dormann A, et al. Molecular profiling-based decision for targeted therapies in IDH wild-type glioblastoma. Neurooncol Adv. 2020 Dec;2(1):vdz060.

87. Lombardi G, Barresi V, Indraccolo S, Simbolo M, Fassan M, Mandruzzato S, et al. Pembrolizumab Activity in Recurrent High-Grade Gliomas with Partial or Complete Loss of Mismatch Repair Protein Expression: A Monocentric, Observational and Prospective Pilot Study. Cancers (Basel). 2020 Aug 14;12(8):E2283.

88. Mishinov SV, Budnik AY, Stupak VV, Leplina OY, Tyrinova TV, Ostanin AA, et al. Autologous and Pooled Tumor Lysates in Combined Immunotherapy of Patients with Glioblastoma. Sovrem Tekhnologii Med. 2020;12(2):34–41.

89. Reardon DA, Desjardins A, Vredenburgh JJ, O’Rourke DM, Tran DD, Fink KL, et al. Rindopepimut with Bevacizumab for Patients with Relapsed EGFRvIII-Expressing Glioblastoma (ReACT): Results of a Double-Blind Randomized Phase II Trial. Clin Cancer Res. 2020 Apr 1;26(7):1586–94.

90. Rudnick JD, Sarmiento JM, Uy B, Nuno M, Wheeler CJ, Mazer MJ, et al. A phase I trial of surgical resection with Gliadel Wafer placement followed by vaccination with dendritic cells pulsed with tumor lysate for patients with malignant glioma. J Clin Neurosci. 2020 Apr;74:187–93.

91. Sampson JH, Achrol A, Aghi MK, Bankiewicz K, Bexon M, Brem S, et al. MDNA55 survival in recurrent glioblastoma (rGBM) patients expressing the interleukin-4 receptor (IL4R) as compared to a matched synthetic control. JCO. 2020 May 20;38(15_suppl):2513–2513.

92. Smith C, Lineburg KE, Martins JP, Ambalathingal GR, Neller MA, Morrison B, et al. Autologous CMV-specific T cells are a safe adjuvant immunotherapy for primary glioblastoma multiforme. J Clin Invest. 2020 Nov 2;130(11):6041–53.

93. Van Den Bent M, Eoli M, Sepulveda JM, Smits M, Walenkamp A, Frenel JS, et al. INTELLANCE 2/EORTC 1410 randomized phase II study of Depatux-M alone and with temozolomide vs temozolomide or lomustine in recurrent EGFR amplified glioblastoma. Neuro Oncol. 2020 May 15;22(5):684–93.

94. Wang QT, Nie Y, Sun SN, Lin T, Han RJ, Jiang J, et al. Tumor-associated antigen-based personalized dendritic cell vaccine in solid tumor patients. Cancer Immunol Immunother. 2020 Jul;69(7):1375–87.

95. Bonneville-Levard A, Frappaz D, Tredan O, Lavergne E, Corset V, Agrapart V, et al. Molecular profile to guide personalized medicine in adult patients with primary brain tumors: results from the ProfiLER trial. Med Oncol. 2021 Nov 5;39(1):4.

96. Cardona AF, Jaramillo-Velásquez D, Ruiz-Patiño A, Polo C, Jiménez E, Hakim F, et al. Efficacy of osimertinib plus bevacizumab in glioblastoma patients with simultaneous EGFR amplification and EGFRvIII mutation. J Neurooncol. 2021 Sep;154(3):353–64.

97. Padovan M, Eoli M, Pellerino A, Rizzato S, Caserta C, Simonelli M, et al. Depatuxizumab Mafodotin (Depatux-M) Plus Temozolomide in Recurrent Glioblastoma Patients: Real-World Experience from a Multicenter Study of Italian Association of Neuro-Oncology (AINO). Cancers (Basel). 2021 Jun 3;13(11):2773.

98. Werlenius K, Stragliotto G, Strandeus M, Blomstrand M, Carén H, Jakola AS, et al. A randomized phase II trial of efficacy and safety of the immunotherapy ALECSAT as an adjunct to radiotherapy and temozolomide for newly diagnosed glioblastoma. Neurooncol Adv. 2021 Dec;3(1):vdab156.

99. Ciesielski MJ, Ahluwalia MS, Reardon DA, Abad AP, Curry WT, Wong ET, et al. Final data from the phase 2a single-arm trial of SurVaxM for newly diagnosed glioblastoma. JCO. 2022 Jun 1;40(16_suppl):2037–2037.

100. Hu JL, Omofoye OA, Rudnick JD, Kim S, Tighiouart M, Phuphanich S, et al. A Phase I Study of Autologous Dendritic Cell Vaccine Pulsed with Allogeneic Stem-like Cell Line Lysate in Patients with Newly Diagnosed or Recurrent Glioblastoma. Clin Cancer Res. 2022 Feb 15;28(4):689–96.

101. Kasenda B, König D, Manni M, Ritschard R, Duthaler U, Bartoszek E, et al. Targeting immunoliposomes to EGFR-positive glioblastoma. ESMO Open. 2022 Feb;7(1):100365.

102. Van Gool SW, Makalowski J, Bitar M, Van de Vliet P, Schirrmacher V, Stuecker W. Synergy between TMZ and individualized multimodal immunotherapy to improve overall survival of IDH1 wild-type MGMT promoter-unmethylated GBM patients. Genes Immun. 2022 Feb 16;

103. Quattrocchi KB, Miller CH, Cush S, Bernard SA, Dull ST, Smith M, et al. Pilot study of local autologous tumor infiltrating lymphocytes for the treatment of recurrent malignant gliomas. J Neurooncol. 1999;45(2):141–57.

104. De Vleeschouwer S, Fieuws S, Rutkowski S, Van Calenbergh F, Van Loon J, Goffin J, et al. Postoperative adjuvant dendritic cell-based immunotherapy in patients with relapsed glioblastoma multiforme. Clin Cancer Res. 2008 May 15;14(10):3098–104.

105. Sampson JH, Archer GE, Mitchell DA, Heimberger AB, Bigner DD. Tumor-specific immunotherapy targeting the EGFRvIII mutation in patients with malignant glioma. Semin Immunol. 2008 Oct;20(5):267–75.

106. Chang CN, Huang YC, Yang DM, Kikuta K, Wei KJ, Kubota T, et al. A phase I/II clinical trial investigating the adverse and therapeutic effects of a postoperative autologous dendritic cell tumor vaccine in patients with malignant glioma. J Clin Neurosci. 2011 Aug;18(8):1048–54.

107. Jie X, Hua L, Jiang W, Feng F, Feng G, Hua Z. Clinical application of a dendritic cell vaccine raised against heat-shocked glioblastoma. Cell Biochem Biophys. 2012 Jan;62(1):91–9.

108. Lv S, Teugels E, Sadones J, De Brakeleer S, Duerinck J, Du Four S, et al. Correlation of EGFR, IDH1 and PTEN status with the outcome of patients with recurrent glioblastoma treated in a phase II clinical trial with the EGFR-blocking monoclonal antibody cetuximab. Int J Oncol. 2012 Sep;41(3):1029–35.

109. Mitsuya K, Akiyama Y, Iizuka A, Miyata H, Deguchi S, Hayashi N, et al. Alpha-type-1 Polarized Dendritic Cell-based Vaccination in Newly Diagnosed High-grade Glioma: A Phase II Clinical Trial. Anticancer Res. 2020 Nov;40(11):6473–84.

110. Prins RM, Wang X, Soto H, Young E, Lisiero DN, Fong B, et al. Comparison of glioma-associated antigen peptide-loaded versus autologous tumor lysate-loaded dendritic cell vaccination in malignant glioma patients. J Immunother. 2013 Feb;36(2):152–7.

111. Müller K, Henke G, Pietschmann S, van Gool S, De Vleeschouwer S, von Bueren AO, et al. Re-irradiation or re-operation followed by dendritic cell vaccination? Comparison of two different salvage strategies for relapsed high-grade gliomas by means of a new prognostic model. J Neurooncol. 2015 Sep;124(2):325–32.

112. Reardon DA, Schuster J, Tran DD, Fink KL, Nabors LB, Li G, et al. ReACT: Overall survival from a randomized phase II study of rindopepimut (CDX-110) plus bevacizumab in relapsed glioblastoma. JCO. 2015 May 20;33(15_suppl):2009–2009.

113. Fenstermaker RA, Ciesielski MJ, Qiu J, Yang N, Frank CL, Lee KP, et al. Clinical study of a survivin long peptide vaccine (SurVaxM) in patients with recurrent malignant glioma. Cancer Immunol Immunother. 2016 Nov;65(11):1339–52.

114. Phuphanich S, Wheeler C, Rudnick J, Hu J, Mazer M, Nuño M, et al. OS2.5 Long term remission/survival over 8 years in patients with newly diagnosed glioblastoma (GBM) treated with ICT-107 dendritic cell-based immunotherapy (phase I). Neuro-Oncology. 2016 Oct 1;18(suppl_4):iv5.

115. Furuta T, Sabit H, Dong Y, Miyashita K, Kinoshita M, Uchiyama N, et al. Biological basis and clinical study of glycogen synthase kinase- 3β-targeted therapy by drug repositioning for glioblastoma. Oncotarget. 2017 Apr 4;8(14):22811–24.

116. Ahluwalia MS, Reardon DA, Abad AP, Curry WT, Wong ET, Belal A, et al. Phase II trial of SurVaxM combined with standard therapy in patients with newly diagnosed glioblastoma. JCO. 2018 May 20;36(15_suppl):2041–2041.

117. Antonios J, Everson R, Soto H, Khattab S, Bethel J, Sun M, et al. ATIM-39. Improved survival noted in glioblastoma patients treated with adjuvant TLR-3 agonist in setting of autologous lysate-pulsed DC vaccination. Neuro Oncol. 2018 Nov;20(Suppl 6):vi10.

118. Byron SA, Tran NL, Halperin RF, Phillips JJ, Kuhn JG, Groot JF de, et al. Prospective Feasibility Trial for Genomics-Informed Treatment in Recurrent and Progressive Glioblastoma. Clin Cancer Res. 2018 Jan 15;24(2):295–305.

119. Jan CI, Tsai WC, Harn HJ, Shyu WC, Liu MC, Lu HM, et al. Predictors of Response to Autologous Dendritic Cell Therapy in Glioblastoma Multiforme. Front Immunol. 2018;9:727.

120. Ranjan T, Howard CM, Valluri J, Yu A, Aziz K, Jho D, et al. Prospective analysis of cancer stem cell drug response assay for glioblastoma patients. JCO. 2018 May 20;36(15_suppl):2057–2057.

121. Boydell E, Marinari E, Migliorini D, Dietrich PY, Patrikidou A, Dutoit V. Exploratory Study of the Effect of IMA950/Poly-ICLC Vaccination on Response to Bevacizumab in Relapsing High-Grade Glioma Patients. Cancers (Basel). 2019 Apr 2;11(4):E464.

122. Caccese M, Simonelli M, Fassan M, Padovan M, Persico P, Bellu L, et al. Pembrolizumab (Pem) in recurrent high-grade glioma (HGG) patients with mismatch repair deficiency (MMRd): An observational study. Annals of Oncology. 2019 Oct 1;30:v155.

123. Garrett AM, Lastakchi S, McConville C. The Personalisation of Glioblastoma Treatment Using Whole Exome Sequencing: A Pilot Study. Genes (Basel) [Internet]. 2020 Feb 6 [cited 2021 Apr 3];11(2). Available from: https://www.ncbi.nlm.nih.gov/pmc/articles/PMC7074406/

124. Hoogstrate Y, Vallentgoed W, Kros JM, de Heer I, de Wit M, Eoli M, et al. EGFR mutations are associated with response to depatux-m in combination with temozolomide and result in a receptor that is hypersensitive to ligand. Neurooncol Adv. 2019 Dec 9;2(1):vdz051.

125. Ranjan T, Howard CM, Yu A, Xu L, Aziz K, Jho D, et al. Cancer Stem Cell Chemotherapeutics Assay for Prospective Treatment of Recurrent Glioblastoma and Progressive Anaplastic Glioma: A Single-Institution Case Series. Transl Oncol. 2020 Apr;13(4):100755.

126. van den Bent M, Azaro A, De Vos F, Sepulveda J, Yung WKA, Wen PY, et al. A Phase Ib/II, open-label, multicenter study of INC280 (capmatinib) alone and in combination with buparlisib (BKM120) in adult patients with recurrent glioblastoma. J Neurooncol. 2020 Jan;146(1):79–89.

127. Batich K, Mitchell D, Healy P, Herndon J, Broadwater G, Michael G, et al. CTIM-10. Reproducibility of clinical trials using CMV-targeted dendritic cell vaccines in patients with glioblastoma. Neuro-Oncology. 2021 Nov 2;23(Supplement_6):vi51.

128. Ishikawa E, Sugii N, Matsuda M, Kohzuki H, Tsurubuchi T, Akutsu H, et al. Maximum resection and immunotherapy improve glioblastoma patient survival: a retrospective single-institution prognostic analysis. BMC Neurol. 2021 Jul 19;21(1):282.

129. Lim-Fat MJ, Youssef GC, Touat M, Iorgulescu JB, Whorral S, Allen M, et al. Clinical utility of targeted next-generation sequencing assay in IDH-wildtype glioblastoma for therapy decision-making. Neuro Oncol. 2022 Jul 1;24(7):1140–9.

130. Sim HW, McDonald KL, Lwin Z, Barnes EH, Rosenthal M, Foote MC, et al. A randomized phase II trial of veliparib, radiotherapy, and temozolomide in patients with unmethylated MGMT glioblastoma: the VERTU study. Neuro Oncol. 2021 Oct 1;23(10):1736–49.

131. Baldini C, Younan N, Castanon Alvarez E, Ammari S, Alentorn A, Dumont S, et al. Genome-driven medicine for patients with recurrent glioma enrolled in early phase trials. Eur J Cancer. 2022 Mar;163:98–107.
